# Supplementary material for: Circulating natural killer cells are phenotypically and functionally altered in age-related macular degeneration
Source: Cell Rep Med. 2026 May 7;7(6):102792. doi: 10.1016/j.xcrm.2026.102792 (PMC13293971; doi:10.1016/j.xcrm.2026.102792)
Supplement: Document S1. Figures S1–S9 and Tables S1 and S2 [file mmc1.pdf]

Cell Reports Medicine, Volume *7*

Supplemental information

Circulating natural killer cells are phenotypically and functionally altered in age-related macular degeneration

Kiva Brennan, Ema Ozaki, Eleanor Noone, Sarah Palko, Kieran P. Byrne, Fiona Roche, Matt McElheron, Kieva Byrne, Luke Gibbons, Katie Robb, Said Aktas, Emma Connolly, Natalie Hudson, Matthew M. O'Riordan, Dara O'Boyle, Rachel Dalton, Aline Zoller, Erin Fahey, Karsten Hokamp, Derrick Feenstra, Nollaig Bourke, Matthew Campbell, David Finlay, Kelly Mulfaul, Robert F. Mullins, Rose Anne Kenny, Mark T. Cahill, and Sarah L. Doyle

A

Supplementary Table 1: Demographics of TILDA cohort with AMD severity

	AMD Severity						
	**No Disease**	**Early Mild**	**Early Moderate**	**Early Severe**	**Late AMD**	**Total**	**P Value**
n (%)	4,169 (95.6%)	78 (1.8%)	81 (1.9%)	27 (0.6%)	8 (0.2%)	4,363 (100.0%)	
Age, *mean(SD)*	60.5 (8.5)	60.4 (7.6)	65.1 (8.7)	65.3 (9.1)	67.0 (11.2)	60.6 (8.5)	<0.001
Sex, *n (%)*							
Male	1,841 (44.2%)	31 (39.7%)	34 (42.0%)	12 (44.4%)	4 (50.0%)	1,922 (44.1%)	0.929
Female	2,327 (55.8%)	47 (60.3%)	47 (58.0%)	15 (55.6%)	4 (50.0%)	2,440 (55.9%)	
Smoking, *n(%)*							
Never/Past	3,533 (84.8%)	65 (83.3%)	77 (95.1%)	24 (88.9%)	8 (100.0%)	3,707 (85.0%)	0.075
Current	635 (15.2%)	13 (16.7%)	4 (4.9%)	3 (11.1%)	0 (0.0%)	655 (15.0%)	
BMI, *mean(SD)*	28.5 (4.9)	28.9 (5.1)	28.1 (4.8)	27.5 (3.9)	27.7 (6.1)	28.5 (4.9)	0.909
Education, *n(%)*							
Primary/none	820 (19.7%)	14 (17.9%)	15 (18.5%)	5 (18.5%)	3 (37.5%)	857 (19.7%)	0.859
Secondary	1,791 (43.0%)	35 (44.9%)	30 (37.0%)	11 (40.7%)	2 (25.0%)	1,869 (42.9%)	
Third/higher	1,556 (37.3%)	29 (37.2%)	36 (44.4%)	11 (40.7%)	3 (37.5%)	1,635 (37.5%)	

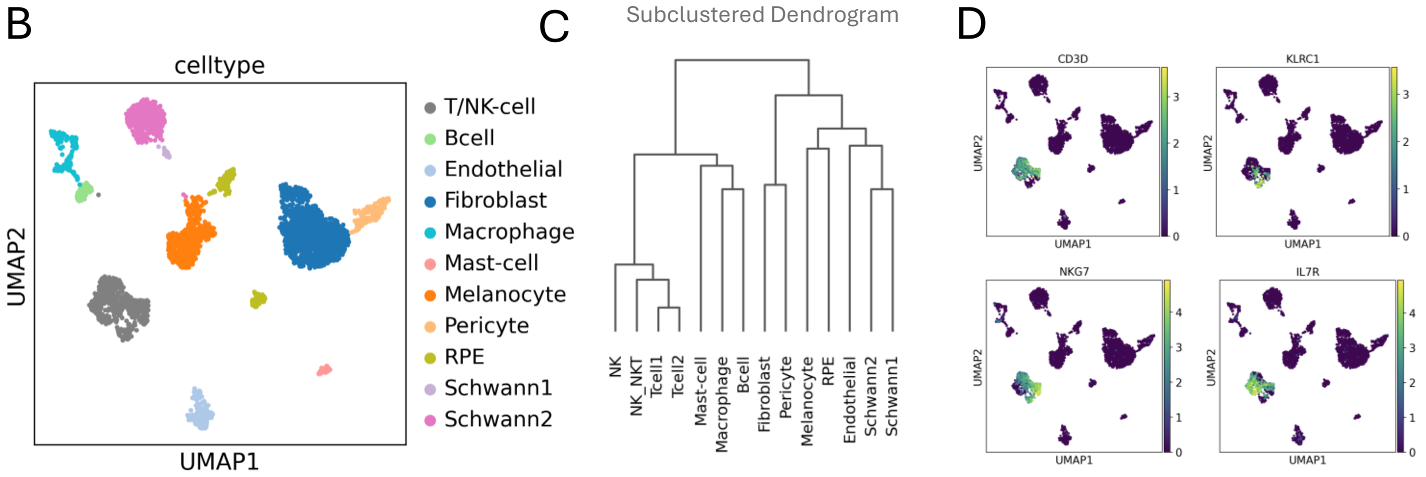

Supplementary Figure 1: Demographics of TILDA cohort, and further analysis of publicly available human donor eye dataset. Related to Figure 1.

(A) Demographics table of TILDA cohort, including disease severity. Following normality testing, Pearson's X^2 was used to compare categorical variables and Kruskal-Wallis was used to compare continuous variables between groups. **(B-D)** Reanalysis of Voigt et al. dataset [12] **(B)** UMAP plot of 3,744 cells derived from peripheral and macular ocular tissue from three human donors showing the presence of 11 cell types based on the expression of specific markers. **(C)** Dendrogram showing the relationship between annotated cell types. **(D)** UMAP plots showing expression distributions, in log scale, of four marker genes across annotated cell types.

A

Supplementary Table 2: Demographics of clinical cohort with AMD severity

	No Disease	Early AMD	Late AMD	Total	P Value
***n** (%)*	33 (52.4)	17 (27.98)	13 (20.6)	63 (100)	
***Age,** mean (SD)*	70.97 (8.64)	71.4 (9.4)	81.69 (9.89)	73.3(9.95)	0.0013
***Sex,** n (%)*					
Male	14 (42.4%)	6 (35.3%)	6 (46.2%)	26 (36.6%)	0.8201
Female	19 (57.6%)	11 (64.7%)	7 (53.8%)	37 (63.4%)	

B

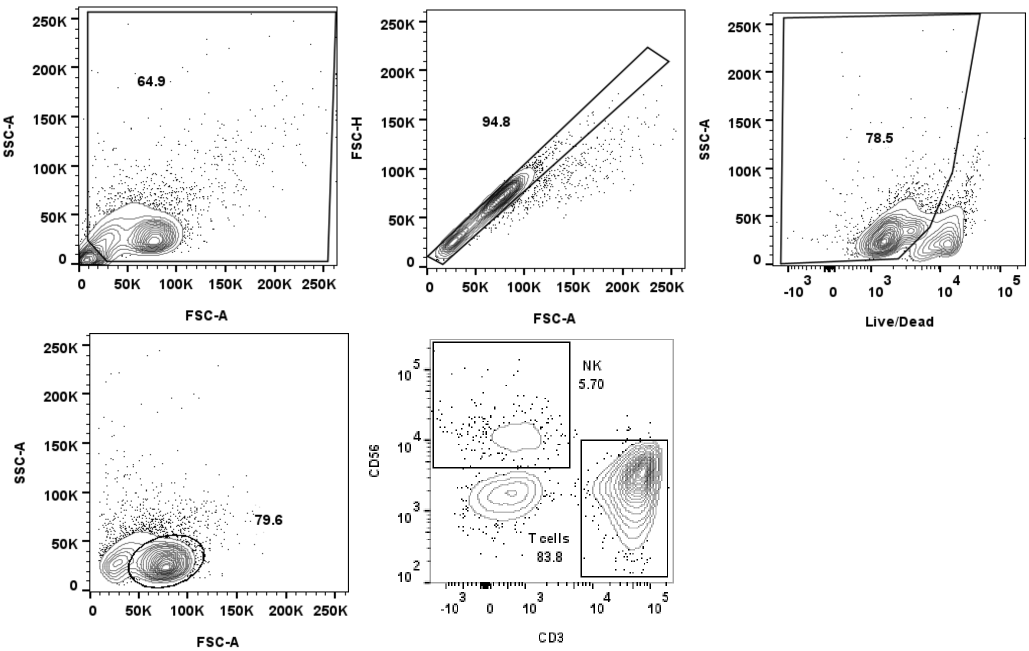

C

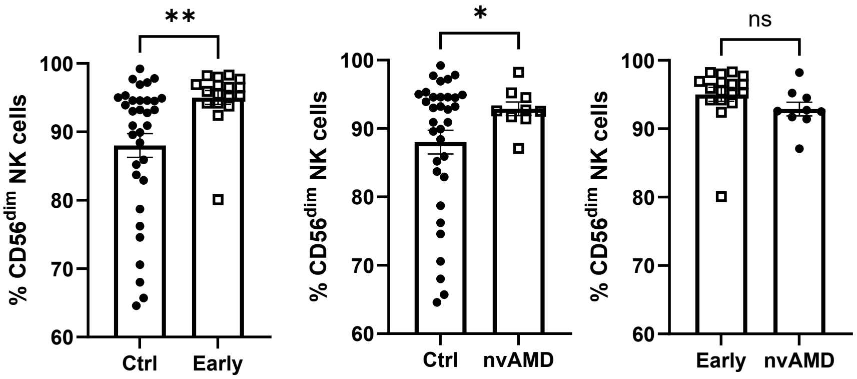

Supplementary Figure 2: Demographics and gating strategy for flow cytometric analysis of NK and T cell subsets in human PBMCs. Related to Figure 1.

(A) Demographics table of clinical sample cohort, including disease severity. Following normality testing, Pearson's X^2 was used to compare categorical variables and Kruskal-Wallis was used to compare continuous variables between groups. **(B)** Representative gating strategy for NK cells and T cells based on expression of CD56 and CD3 **(C)** CD56^{dim} NK cells expressed as a percentage of total NK cells divided according to AMD stage (n=26 Ctrl, n= 17 early AMD, n=9 nvAMD). Graphical data are presented as mean ±SEM. Following normality testing, unpaired t-tests or one-way ANOVA with Dunnett's multiple comparison tests were used to compare groups.

A

Antigen	Fluorophore	Clone	Cat no.	Supplier
CD45	BB515	30-F11	564590	BD
NK1.1	BB700	PK136	556503	BD
CD3	VioBlue	17A2	130-118-849	Miltenyi
CD4	AF700	GK1.5	100429	Biolegend
CD8	APCCy7	53-6.7	100713	Biolegend
Ki67	PECy7	SolA15	25-5698-80	Invitrogen
CD69	BV650	H1.2F3	104541	Biolegend
CD25	PE	PC61.5	12-0251-81	Invitrogen

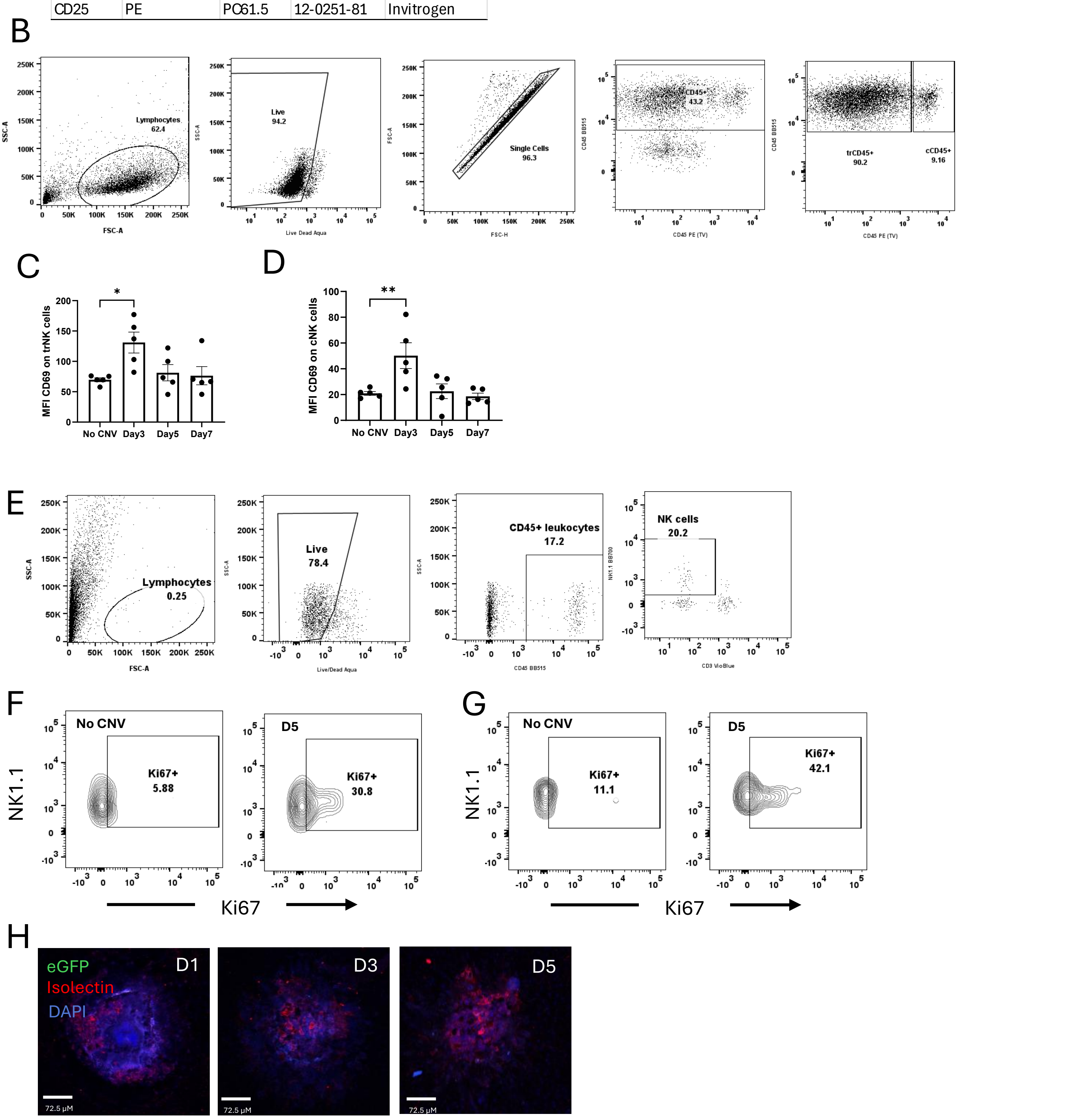

Supplementary Figure 3: Splenocyte & RPE/choroid flow cytometry staining and gating. Related to Figures 2 and 3.

(A) Flow cytometry antibodies, including fluorophore, clone, catalogue number and supplier used in Figures 2 & 3. **(B)** Gating strategy for identification of lymphocyte populations from spleen. **(C)** MFI of CD69 on tissue resident and **(D)** circulating splenic NK cells following liCNV. **(E)** Gating strategy for identification of lymphocyte populations from RPE/choroid. **(F & G)** Representative Ki67 gating for total RPE/choroid NK cells & tissue resident RPE/choroid NK cells. **(H)** Representative RPE/choroid flatmounts from NCR1-GFP^{-} mice at 1, 3 and 5 days post liCNV (2 eyes/timepoint, 2 liCNV/eye).

A

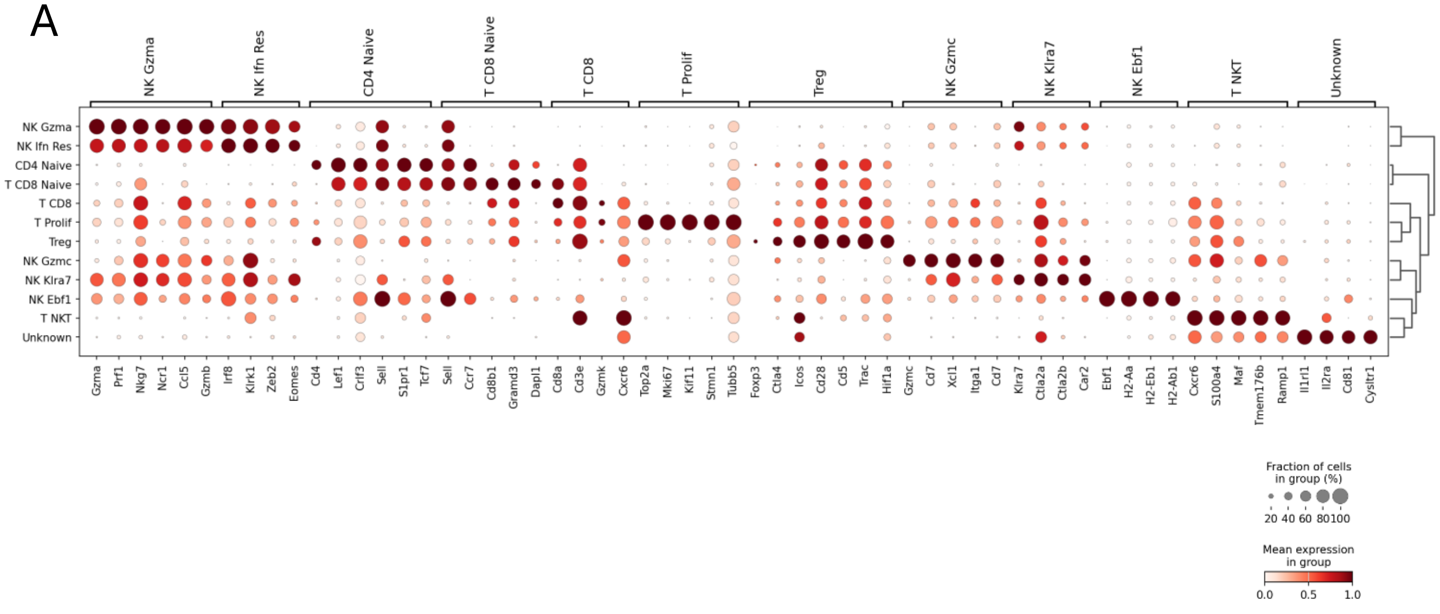

B

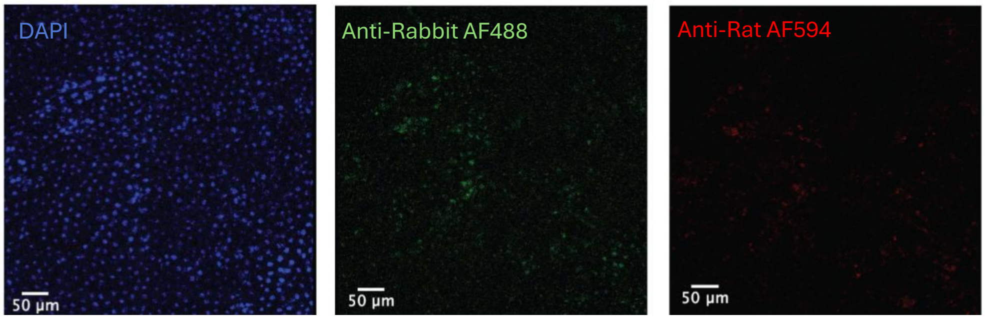

Supplementary Figure 4: scRNA sequencing analysis from healthy vs liCNV cohort and control staining for RPE/choroid flatmounts. Related to Figure 3.

(A) Dotplot showing the expression pattern of known marker genes across cell types from scRNA sequencing experiment comparing liCNV versus control RPE/choroid tissue. Dot colour indicates the average expression level within each donor. Dot size indicates the fraction of cells in each group that express a particular gene. **(B)** Staining controls for RPE/choroid flatmounts. Secondary only controls for DAPI, anti-rabbit AF488 and anti-rat AF594 on RPE/choroid flatmounts.

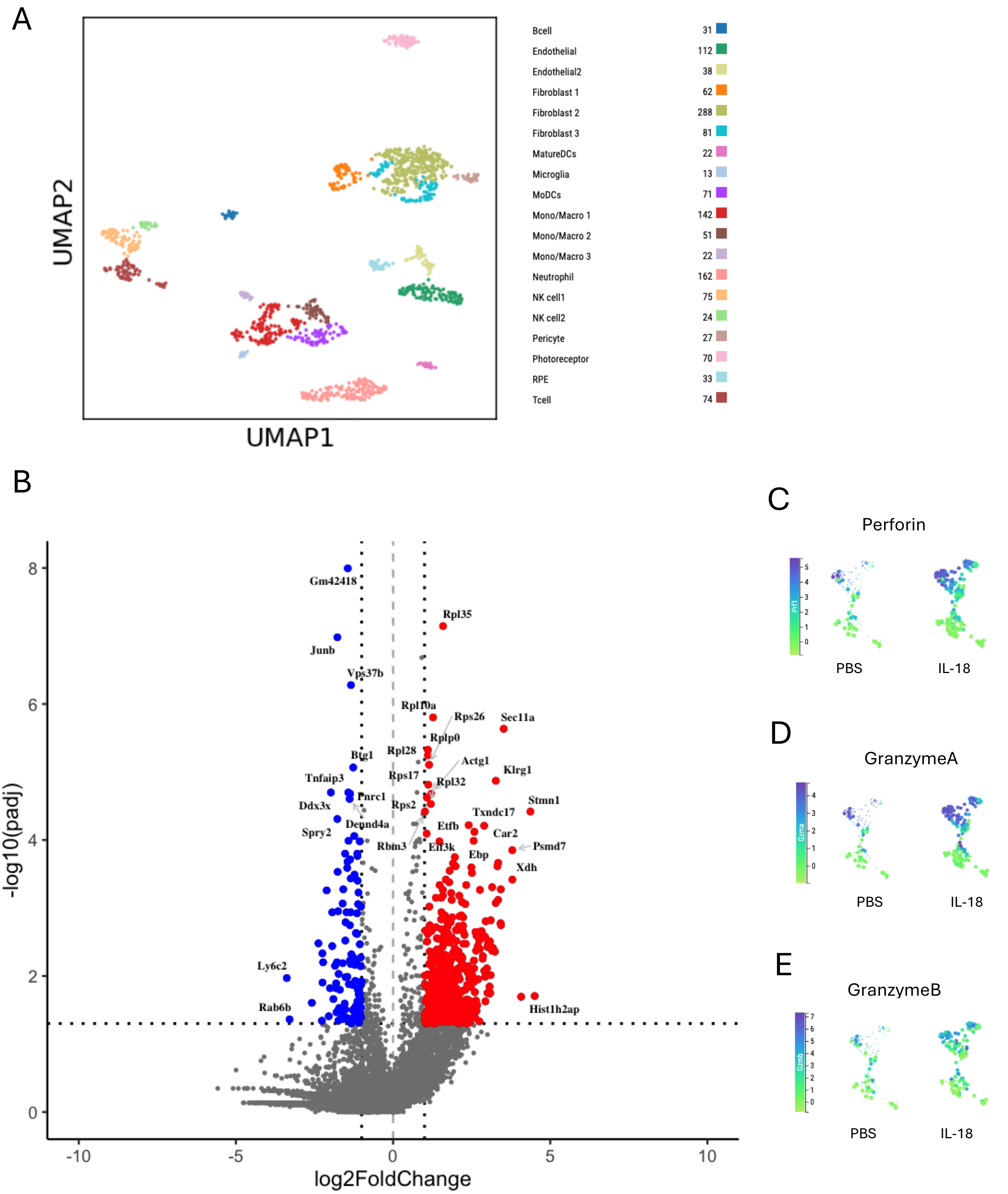

Supplementary Figure 5: Cell clustering analysis of scRNA sequencing data from RPE/choroid of PBS and IL-18. Related to Figure 4.

(A) UMAP of clusters (Scanpy analysis) of CNV tissue from vehicle and IL-18 treated mice (n=8 mice) **(B)** Volcano plot showing the genes that are differentially expressed in the NK cell group (NK1 & NK2) between IL-18 vs PBS treatment. **(C-E)** Expression of cytolytic genes *Perforin*, *GranzymeA* and *GranzymeB* in RPE/choroid of mice administered with a liCNV and treated with PBS or IL-18.

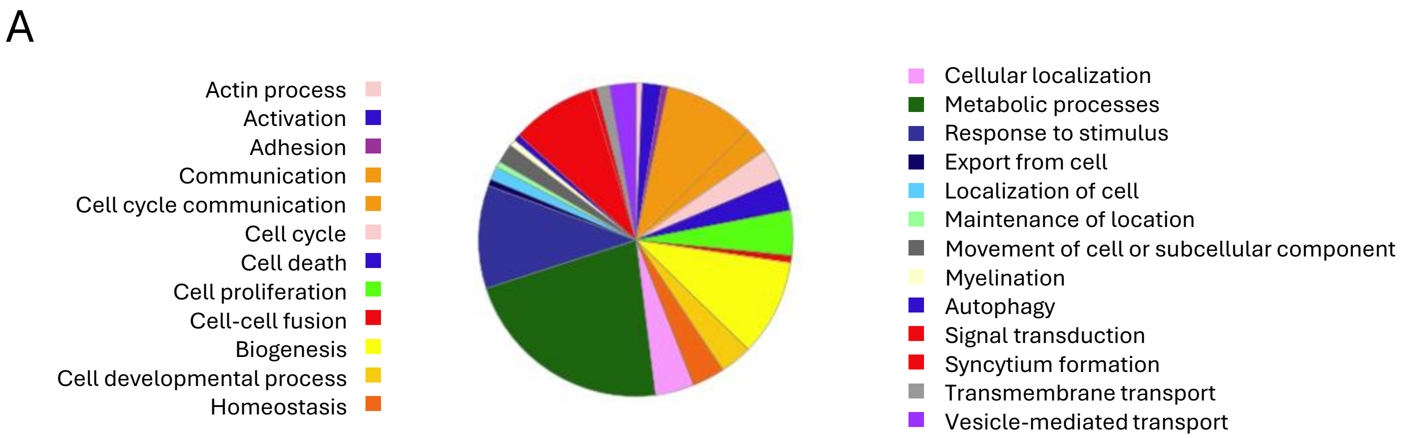

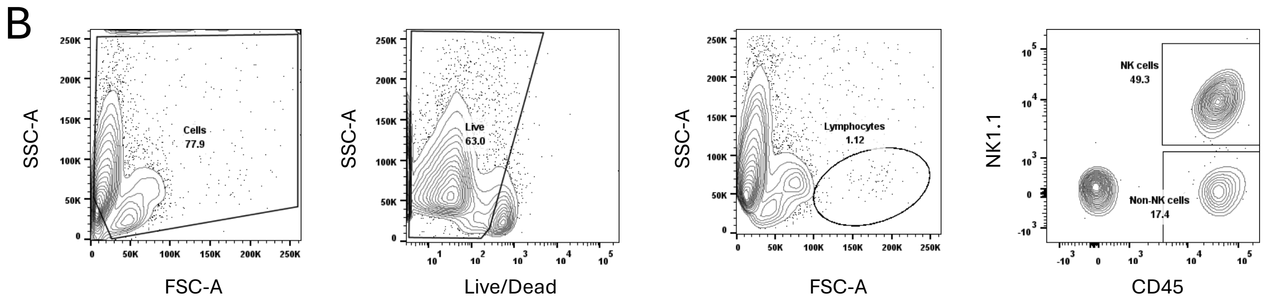

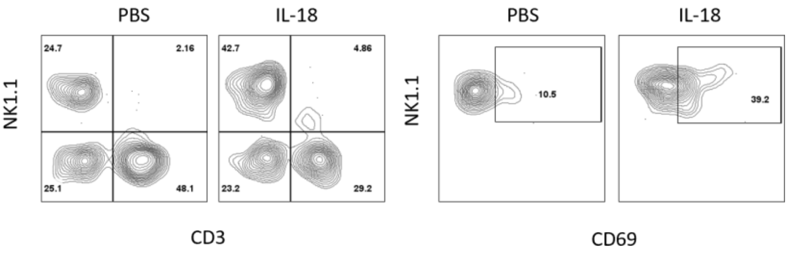

C

Antigen	Fluorophore	Clone	Cat no.	Supplier
IFNy	PECy7	XMG1.2	505825	Biolegend
CD3	FITC	17A2	100204	Biolegend
CD69	PECy5	H1.2F3	104509	Biolegend
NK1.1	e450	PK136	48-5941-82	Invitrogen

Supplementary Figure 6: Functional enrichment analysis of scRNA sequencing data from RPE/choroid of PBS and IL-18, and flow cytometry gating on RPE/choroid. Related to Figure 4.

(A) Full list of gene processes upregulated in NK2 vs NK1 cells in PBS vs IL-18 treated ocular tissue (n=8 eyes/group). **(B)** Flow cytometry gating strategy for NK cells from RPE/choroid **(C)** Flow cytometry antibodies, including fluorophore, clone, catalogue number and supplier.

A

Gene	Primer	Sequence (5'-3')
p21	For	GGCAGACCAGCATGACAGATTTC
	Rev	AACCTCTCATTTCAACCGCCT
Serpine1	For	GGTTCTGCCCAAGTTCTCCC
	Rev	CACCGTGCCACTCTCGTTCA
MicA	For	GAATCCGGCGTAGTCCTGAG
	Rev	TCCGGGGATAGAAGCTGGAA
MicB	For	CTGCCCAGACAAGACGTAGG
	Rev	CGCCTCTCCTTGGATTCCTC
ULBP2	For	TGTGCCTGAGGACATGGCGA
	Rev	CATTACTTCTCAATGGGAGACTGT
ULBP5	For	TGGACAACGGTTCATCCTGG
	Rev	ACCCCACGAGGAGGTCATT
ULBP6	For	TCCAGGATGACCTAGGGTGG
	Rev	AGGATGAAGCAGGGGAGGAT
UBC	For	ATTTGGGTCGCGGTTCTTG
	Rev	TGCCTTGACATTCTCGATGGT
RPL0	For	TGGCGCAGCCAATAGACAG
	Rev	CGCATCATGGTGTTCTTGCC

B

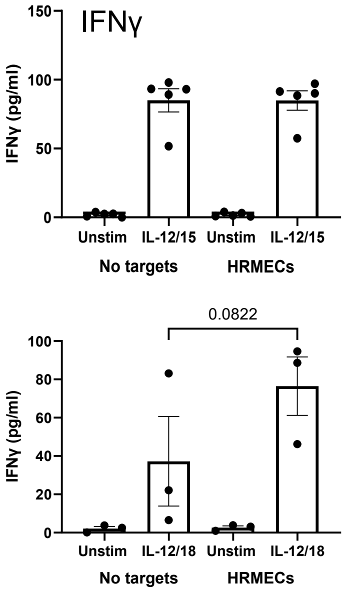

C

Antigen	Fluorophore	Clone	Cat no.	Supplier
CD56	PECy7	HCD56	318318	Biolegend
CD3	FITC	UCHT1	300406	Biolegend
CD107a	APCCy7	H4A3	328620	Biolegend
IFNy	PE	B27	506507	Biolegend

D

Disease Status	Age	Sex
Control	79	Male
Control	87	Male
Control	88	Male
CNV	79	Male
CNV	87	Male
CNV	88	Male

Supplementary Figure 7: Primer sequences for HRMEC investigations; IFNγ production from NK cells in a PBMC population in the presence of HRMECs. Related to Figure 5.

(A) Forward and reverse primer sequences for genes involved in hypoxia and senescence, and NKG2DL genes **(B)** Flow cytometry analysis of IFNγ production from PBMC NK cells in response to cytokine stimulation and incubation with HRMECs **(C)** Flow cytometry antibodies, including fluorophore, clone, catalogue number and supplier. **(D)** Characteristics of donor tissue used for Immunohistochemistry. Graphical data are presented as mean ±SEM. Following normality testing, unpaired t-tests or one-way ANOVA with Dunnett's multiple comparison tests were used to compare groups.

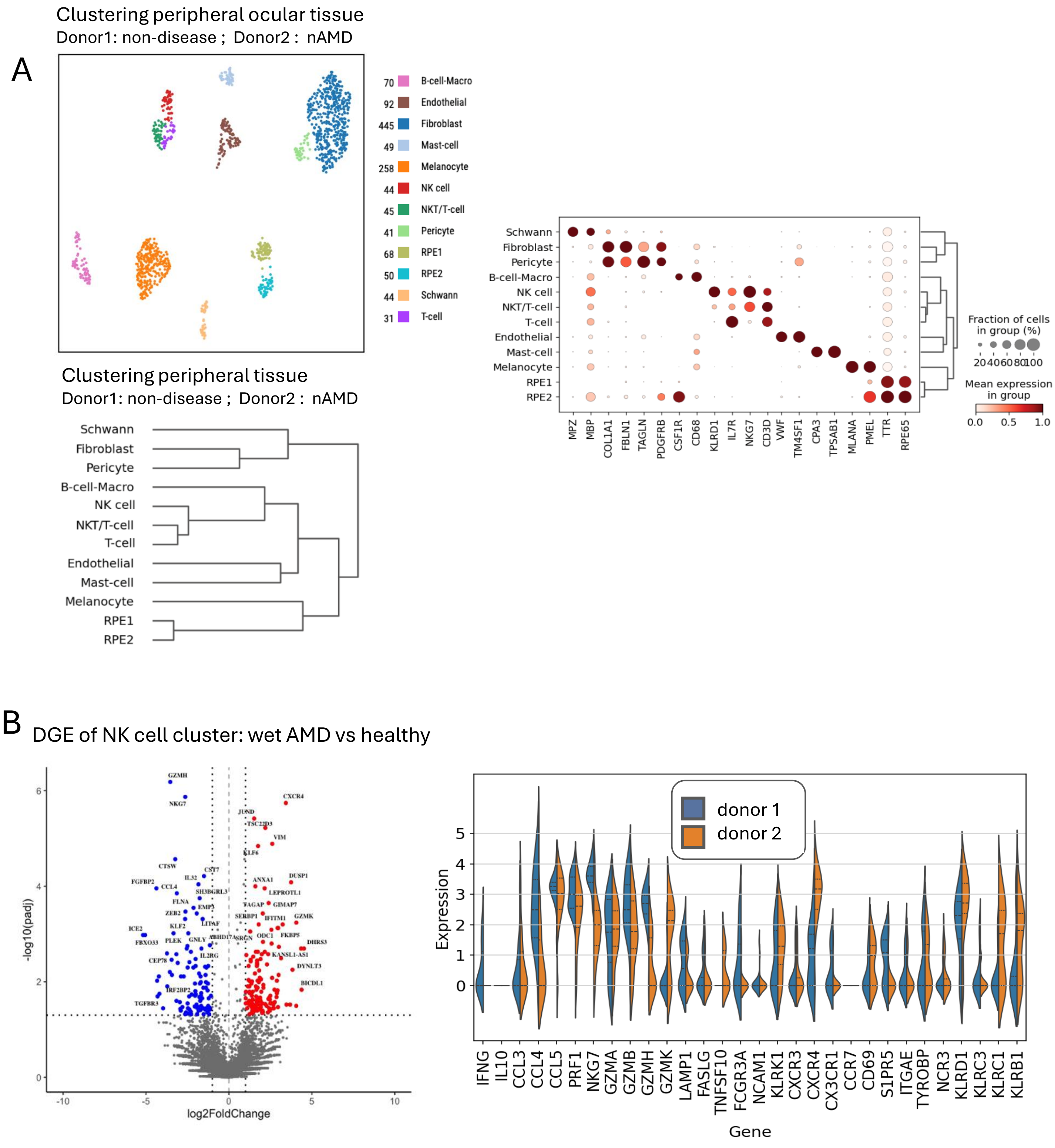

Supplementary Figure 8: Analysis of scRNA sequencing data from non-disease and nAMD donor ocular tissue. Related to Figure 6.

(A) UMAP plot, dendrogram and dot plot analysis of 1,237 cells derived from peripheral ocular tissue from two human donors (nAMD and non-disease) showing the presence of 12 cell types based on the expression of specific markers. **(B)** Volcano plot and violin plots of differentially expressed genes (DEGs) in RPE/choroid of nAMD donor vs healthy non-disease donor

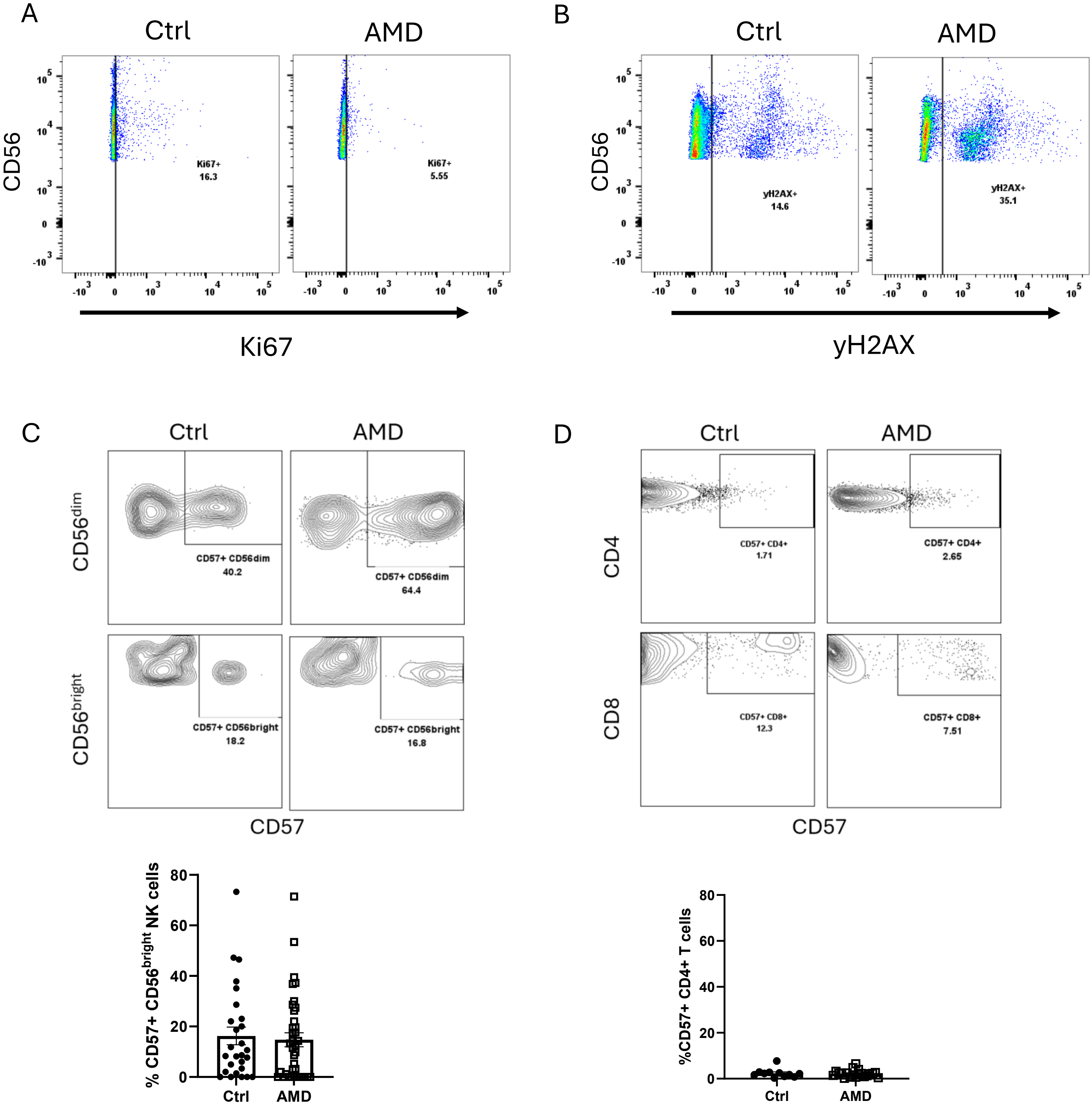

Supplementary Figure 9: Flow cytometric analysis of Ki67 and gH2AX on NK cells and CD57 expression on CD56^{bright} NK cells and CD4^{+} T cells. Related to Figure 6.

(A & B) Representative flow cytometry gating of Ki67 **(A)** and yH2AX **(B)** on CD3^{-}CD56^{+} NK cells in control or AMD donors. **(C)** Representative flow cytometry gating of CD57^{+} expression on CD56^{dim} NK cells (upper panel) and CD56^{bright} NK cells (lower panel)(n=29 Ctrl, n=37 AMD) **(D)** Representative flow cytometry gating of CD57^{+} cells on CD4^{+} (upper panel) and CD8^{+} (lower panel) T cells in control or AMD donors. CD57^{+} expression on CD4^{+} T cells (n=11 Ctrl, n=20 AMD). Graphical data are presented as mean ±SEM. *P < 0.05 **P < 0.01. Following normality testing, unpaired t-tests or one-way ANOVA with Dunnett's multiple comparison tests were used to compare groups.
