# Supplementary material for: Circulating natural killer cells are phenotypically and functionally altered in age-related macular degeneration
Source: Cell Rep Med. 2026 May 7;7(6):102792. doi: 10.1016/j.xcrm.2026.102792 (PMC13293971; doi:10.1016/j.xcrm.2026.102792)
Supplement: Document S2. Article plus supplemental information [file mmc2.pdf]

Cell Reports Medicine

Article

Circulating natural killer cells are phenotypically and functionally altered in age-related macular degeneration

Graphical abstract

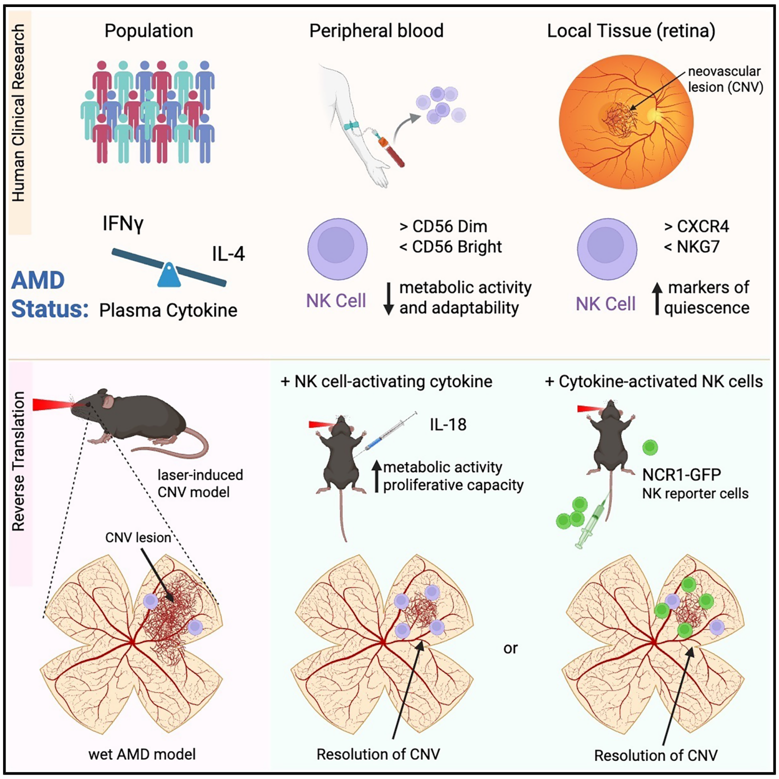

Highlights

- Dysregulation of lymphocytic cytokines and NK cell phenotype in individuals with AMD
- NK cells localize to neovessels in CNV lesions in human disease and murine model
- NK cells display decreased proliferative capacity and increased markers of exhaustion in AMD
- NK cell immunotherapy reduces neovascular lesions in mouse models of nAMD

Authors

Kiva Brennan, Ema Ozaki, Eleanor Noone, ..., Rose Anne Kenny, Mark T. Cahill, Sarah L. Doyle

Correspondence

sarah.doyle@tcd.ie

In brief

Age-related macular degeneration (AMD) is the leading cause of irreversible central blindness in the world, with neovascular complications. Using a "human-first" approach Brennan et al. identify a distinct, functionally altered NK cell phenotype in AMD and suggest that harnessing NK cell activity represents an immunotherapeutic approach for AMD.

Brennan et al., 2026, Cell Reports Medicine 7, 102792
June 16, 2026 © 2026 The Authors. Published by Elsevier Inc.
https://doi.org/10.1016/j.xcrm.2026.102792

CellPress

Cell Reports Medicine

CellPress
OPEN ACCESS

Article

Circulating natural killer cells are phenotypically and functionally altered in age-related macular degeneration

Kiva Brennan,[1,2,13] Ema Ozaki,[1,2,3,13] Eleanor Noone,[1,2] Sarah Palko,[1,2] Kieran P. Byrne,[1,2] Fiona Roche,[4] Matt McElheron,[5] Kieva Byrne,[1,2] Luke Gibbons,[1,2] Katie Robb,[1,2,6] Said Aktas,[7] Emma Connolly,[5] Natalie Hudson,[4] Matthew M. O'Riordan,[4,6] Dara O'Boyle,[1,2] Rachel Dalton,[1,2] Aline Zoller,[1,2] Erin Fahey,[1,2] Karsten Hokamp,[4] Derrick Feenstra,[3] Nollaig Bourke,[5] Matthew Campbell,[4] David Finlay,[8,9] Kelly Mulfaul,[10,11] Robert F. Mullins,[11] Rose Anne Kenny,[5,12] Mark T. Cahill,[6] and Sarah L. Doyle[1,2,14,*]

[1]Department of Clinical Medicine, School of Medicine, Trinity College Dublin, Dublin, Ireland
[2]Trinity College Institute of Neuroscience (TCIN), Trinity College Dublin, Dublin, Ireland
[3]Roche Pharma Research and Early Development, Roche Innovation Center Basel, F. Hoffmann-La Roche Ltd, Basel, Switzerland
[4]Smurfit Institute of Genetics, School of Genetics and Microbiology, Trinity College Dublin, Dublin, Ireland
[5]Department of Medical Gerontology, School of Medicine, Trinity College Dublin, Dublin, Ireland
[6]Progressive Vision Research, Sandyford, Dublin, Ireland
[7]Pharma Research and Early Development, Roche Innovation Center Zurich, Roche Glycart AG., Schlieren, Switzerland
[8]School of Biochemistry and Immunology, Trinity College Dublin, Dublin, Ireland
[9]Trinity Biomedical Sciences Institute, Trinity College Dublin, Dublin, Ireland
[10]Department of Neuroscience and Pharmacology, University of Iowa, Iowa City, IA, USA
[11]Institute for Vision Research, University of Iowa, Iowa City, IA, USA
[12]Mercer's Institute for Successful Ageing (MISA) St. James's Hospital, Dublin, Ireland
[13]These authors contributed equally
[14]Lead contact
*Correspondence: sarah.doyle@tcd.ie
https://doi.org/10.1016/j.xcrm.2026.102792

SUMMARY

Age-related macular degeneration (AMD) is the leading cause of irreversible central blindness and can result in pathological neovascularization. Using a "human-first" approach, we identify immunotherapy as a disease modifier in models of neovascular AMD (nAMD). Plasma cytokine analysis in a large population cohort reveals an imbalance of lymphocytic cytokines associated with severity of AMD, leading to discovery of a skewed peripheral natural killer (NK) cell phenotype in individuals with AMD. Peripheral NK cells are rapidly activated in nAMD models, and single-cell RNA sequencing demonstrates expansion of activated cytolytic NK cells within neovascular lesions during resolution. NK cells localize to neovessels in human AMD donor eyes; however, they exhibit markers of terminal differentiation and quiescence. Adoptive transfer of pre-activated NK cells reduces neovascularization and restores barrier integrity. Our data identify a distinct, functionally altered NK cell phenotype in nAMD and suggests harnessing NK cells represents an immunotherapeutic alternative for the treatment of nAMD.

INTRODUCTION

Age-related macular degeneration (AMD) is a progressive disease affecting central vision; late-stage disease can present with pathological neovascularization (nAMD) and/or geographic atrophy (GA) of retinal pigment epithelium (RPE), as well as atrophy of photoreceptors and choriocapillaris.[1] nAMD is the most common cause of vision loss in people over the age of 50 years and is characterized by growth of abnormal new blood vessels originating most frequently from the choroid, termed choroidal neovascularization (CNV).[2] These vessels grow within the sub-RPE or subretinal space leaking exudative material, disrupting the architecture of the retina, resulting in blindness. While current standard-of-care anti-VEGF-based therapies are effective in preventing vision loss in many patients,[3] emerging long-term studies indicate that approximately half of patients develop treatment resistance within 2 years due to onset of subretinal fibrosis and atrophy of the RPE.[4,5] Additionally, approximately a third of nAMD patients are refractory to anti-VEGF treatment from disease onset.[6,7]

Treatment is limited by an incomplete understanding of the mechanisms that trigger pathology. Identification of new biological processes therefore represents a critical clinical need. This study aimed to identify relevant immune-related processes involved in the pathology of human disease through analysis of peripheral plasma cytokines in a large-scale population cohort

© 2026 The Authors. Published by Elsevier Inc.
This is an open access article under the CC BY license (http://creativecommons.org/licenses/by/4.0/).

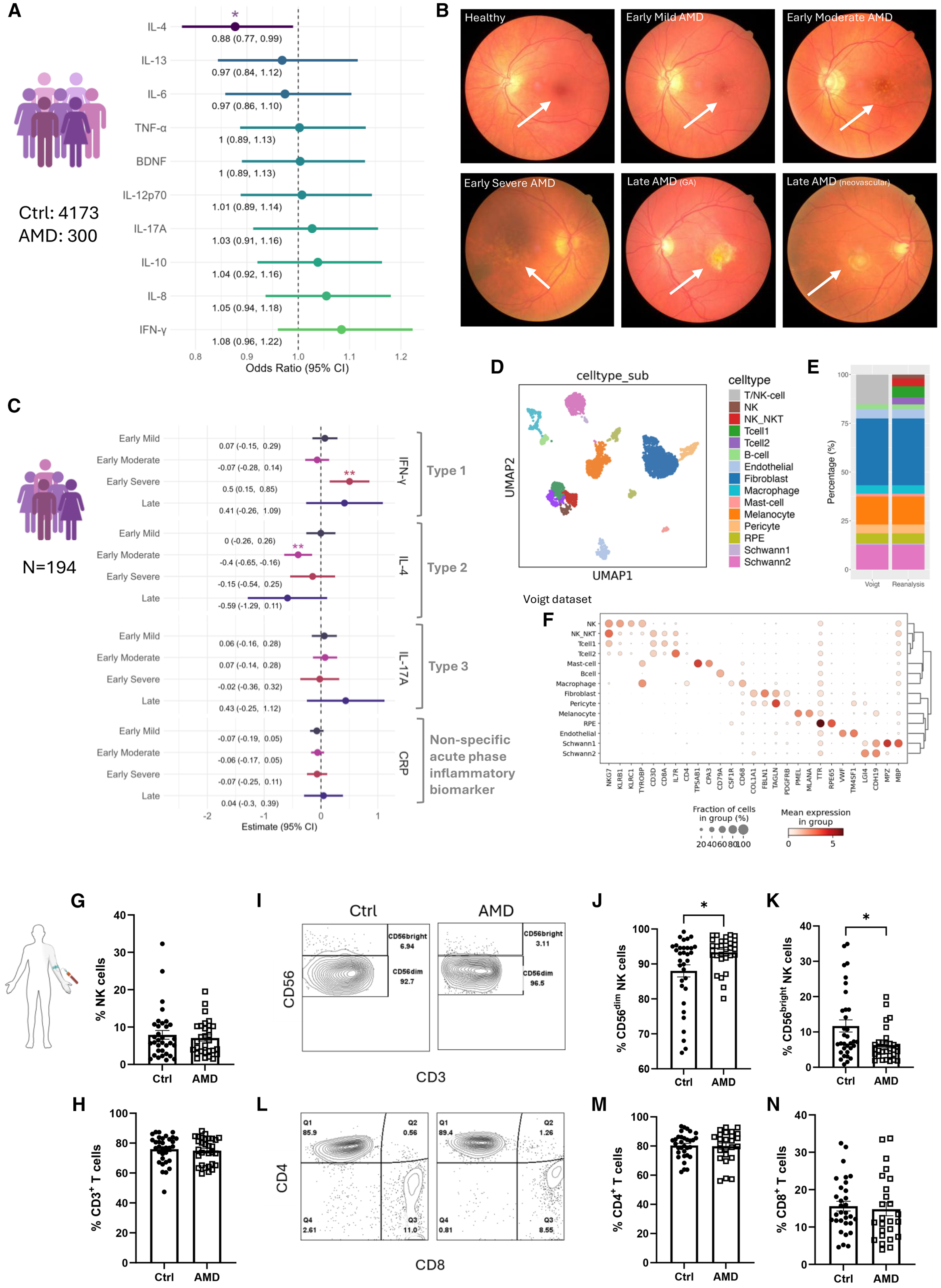

(legend on next page)

and reverse translation into mouse models of retinal neovascular disease for molecular interrogation. We found a clear dichotomy between mutually antagonistic type 1 and type 2 signature cytokines interferon-γ (IFN-γ) and interleukin (IL)-4 as disease progressed. IL-4 is produced primarily by $CD4^+$ T lymphocytes and IFN-γ by natural killer (NK) cells, an innate lymphocyte population of cells, known for their cytotoxic capabilities. This led us to investigate lymphoid cells at a granular level in a case-control cohort, where we found that circulating $CD56^{dim}$ NK cells are overrepresented in the peripheral blood of AMD donors. The healthy retina is an immune privileged tissue practically devoid of infiltrating immune cells. However, immune cells do infiltrate the subretinal space in retinal disease, and there is a growing literature reporting retinal degeneration coinciding with the appearance of myeloid cells in retinal tissue,[8,9] with myeloid cell infiltration consistently associated with detrimental effects.[10] Reports on lymphoid cells and in particular innate lymphoid cells (ILCs) in the retina remain scarce; however, recent evidence suggests that NK cells are involved in slowing the progression of neovascularization in mice through promotion of neutrophil extracellular trap formation, with NK cell depletion studies in mice exacerbating lesion size in a mouse model of neovascularization.[11]

Here, we propose a mechanism whereby NK cells may exert a protective effect on nAMD directly through their cytolytic activity. However, we also observe a diminished proliferative capacity and evidence of NK cell exhaustion in individuals with AMD, implying that cytolytic-mediated clearance of neovascular lesions may be impaired enabling progression of the disease. Immunotherapeutic replenishment of NK cells, with *ex vivo* activated NK cells, resulted in resolution of neovascular lesions in mouse models of disease and may represent a cell-based immunotherapeutic alternative for the treatment of retinal neovascular disease.

RESULTS

Variation of type 1 and type 2 signature cytokines is associated with severity of disease in AMD

A range of plasma cytokines from individuals recruited into The Irish Longitudinal study on Ageing (TILDA) was examined in relation to their AMD status. We observed a variation in the peripheral cytokine signature in AMD, characterized by a significant decrease in levels of the type 2 cytokine IL-4, primarily produced by Th2 cells and ILC2s, and a trend toward increased levels of the type 1 cytokine IFN-γ, which is predominantly produced by Th1 cells, NK cells, and ILC1s (Figure 1A). IFN-γ and IL-4 potently regulate each other's expression; therefore, this observed dichotomy in cytokine signatures strongly indicates that AMD status associates with diminished type 2 immunity and enhanced type 1 immunity in the periphery. IL-17 is the signature cytokine for type 3 immunity, produced by Th17 cells and ILC3s, but this does not appear to associate with AMD status.

In the TILDA cohort, early AMD was classified into three stages: "Early Mild," where >10 hard macular drusen <63 μm can be identified; "Early Moderate," where at least one soft drusen >125 μm is present; and "Early Severe," where soft drusen and hyperpigmentation are identified. These classifications correspond to "Normal Aging," "Early AMD," and "Intermediate AMD," respectively, in the Beckman Classification system. Late AMD can be either Late Geographic Atrophy AMD (GA), identified by the presence of atrophic lesions, or Late Neovascular AMD (nAMD), with proliferation of blood vessels causing macular edema and disciform scar. Representative fundus images of the AMD grades from the TILDA cohort are shown in Figure 1B. To determine if the imbalance in type 1 vs. type 2 immunity remained or was further associated with severity of disease, we assessed levels of IFN-γ, IL-4, and IL-17A in relation to disease severity. Decreased IL-4 was significantly associated with early moderate disease (Figure 1C). The trend toward increased IFN-γ became significant after disease onset in early severe disease. Very little variation was observed for IL-17A over the different stages of AMD though it was somewhat increased in late AMD. Notably, CRP, an acute phase inflammatory marker, remained unchanged throughout the course of disease, emphasizing the specificity of cytokine dichotomy we observed (Figure 1C).

IL-4 is produced primarily by $CD4^+$ T lymphocytes and IFN-γ primarily by NK cells. To assess these populations in the RPE/choroid of human AMD, we reanalyzed a publicly accessible integrated retina, RPE, and choroid dataset from human donor eye tissue.[12–15] We found the lymphocytic cell cluster in the RPE/choroid that was previously reported but not further analyzed by Voigt et al.[12] (Figure S1B). Following sub-clustering, we found these cells could be further divided into several distinct populations, including two T cell clusters, an NK cell cluster and NK_NKT cluster (Figures 1D and S1C). These lymphocyte clusters accounted for ~15% of the cells present (Figure 1E). Cluster

Figure 1. Imbalance of peripheral lymphocytic type 1 and type 2 signature cytokines associated with incidence and severity of AMD

(A) Association of lymphocytic cytokines with AMD occurrence in TILDA (n = 4,173 Ctrl; n = 300 AMD); see Figure S1A.

(B) Representative fundus images illustrating progressive AMD stages from 194 graded participants.

(C) Association of IFN-γ (type 1), IL-4 (type 2), IL-17 (type 3) cytokines, and CRP with different stages of AMD.

(D and E) Reanalysis of Voigt et al. scRNA-seq dataset[12]; see Figures S1B–S1D. (D) UMAP plot of 3,744 cells from peripheral and macular ocular tissue of three human donors. (E) Stacked bar chart showing the percentage cell composition of each cell type from original Voigt et al. analysis and current reanalysis. Color-coded as in Figure 1D.

(F) Dot plot of known marker gene expression across cell types. Dot color indicates average expression. Dot size indicates fraction of cells in each group expressing a particular gene.

(G and H) $CD56^+CD3^-$ NK cells and $CD56^-CD3^+$ T cells expressed as a percentage of total lymphocytes (n = 33 ctrl; n = 30 AMD); see Figures S2A and S2B.

(I–K) (I) Representative gating of $CD56^{bright}$ and $CD56^{dim}$ NK cells in control and AMD donors. (J) $CD56^{dim}$ and (K) $CD56^{bright}$ NK cells as percentage of total NK cells (n = 33 Ctrl; n = 30 AMD); see also Figure S2C.

(L) Representative gating of $CD4^+$ and $CD8^+$ T cells. (M) $CD4^+$ and (N) $CD8^+$ T cells expressed as a percentage of total $CD3^+$ T cells (n = 31 Ctrl; n = 25 AMD). Data are mean ± SEM. *$p < 0.05$, **$p < 0.01$. Mann-Whitney or unpaired t tests after normality testing.

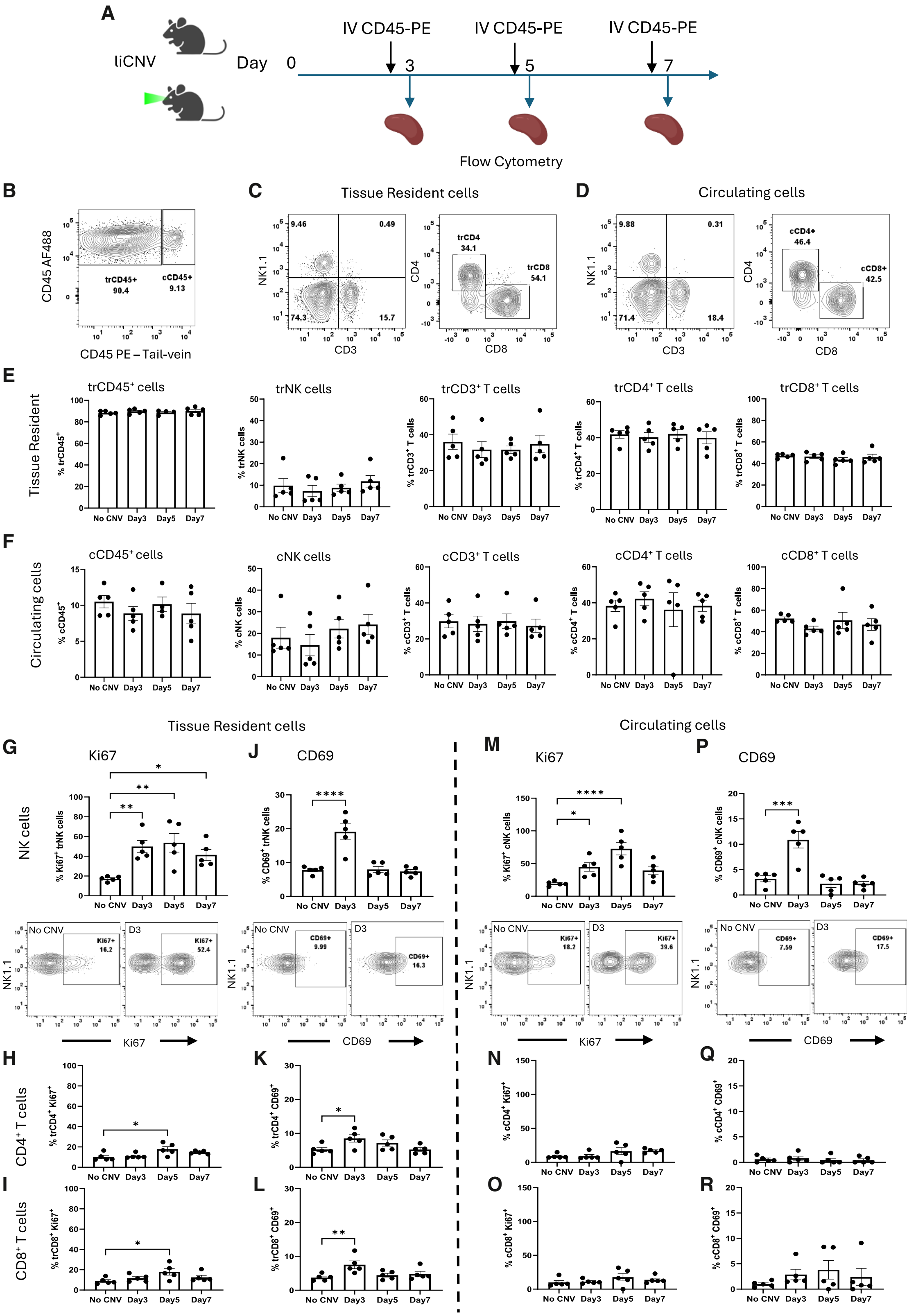

(legend on next page)

CellPress
OPEN ACCESS

identity was determined by expression of specific markers of T cells and NK cells (*CD3D*, *IL7R*, *NKG7*, and *KLRC1*) (Figures 1F and S1D).

Distinct populations of NK cells are found in the periphery of people with AMD

Due to the presence of T cell and NK cell clusters in the RPE/choroid of human donor eye tissue, we were interested in characterizing lymphocytes in the periphery of individuals with and without AMD. We performed flow cytometry analysis on peripheral blood mononuclear cells (PBMCs) isolated from individuals with AMD, along with age-matched, non-disease controls (Figure S2A), gating on $CD56^{+}CD3^{-}$ NK cells and $CD56^{-}CD3^{+}$ T cells (Figure S2B). We found no difference in the numbers of NK cells (Figure 1G) or T cells (Figure 1H) as a percentage of total lymphocytes. However, following further analysis of these populations, we found differences in the percentages of distinct populations of NK cells. $CD56^{bright}$ and $CD56^{dim}$ NK cells are phenotypically different, with $CD56^{bright}$ NK cells considered more immune-modulatory and more efficient producers of cytokines, while $CD56^{dim}$ NK cells are more cytotoxic.[16] Circulating $CD56^{dim}$ NK cells are significantly overrepresented in peripheral blood of individuals with AMD compared to non-AMD age-matched controls ($p = 0.038$) (Figures 1I and 1J), with a commensurate decrease in $CD56^{bright}$ NK cells ($p = 0.0174$) (Figures 1I and K). When frequency of $CD56^{dim}$ NK cells was examined in context of disease stage, no significant difference was apparent between NK subpopulations in early or late-stage AMD (Figure S2C). Strikingly, we observed no differences in $CD4^{+}$ T helper cells or $CD8^{+}$ cytotoxic T lymphocytes (Figures 1L–1N), indicating a potential role specifically for NK cells in response to AMD.

Peripheral NK lymphocytes are activated by focal tissue injury to the retina

Our data implied that peripheral NK cells respond to retinal pathology in human AMD, while $CD4^{+}$ and $CD8^{+}$ T cells did not. To model this in mice, we used the laser-induced choroidal neovascularization (liCNV) model, in which laser injury drives choroidal vessels through Bruch's membrane into the subretinal space. Using this acute injury model, we assessed peripheral immune activation by analyzing spleens from mice at days 3, 5, and 7 after laser injury and comparing them with uninjured controls (Figure 2A). Intravenous (i.v.) phycoerythrin (PE)-conjugated anti-CD45 was administered via tail vein injection prior to sacrificing mice to discriminate between tissue-resident and circulating lymphocytes (Figures 2B–2D; S3B). The number of tissue-resident splenic $NK1.1^{+}$ NK cells and $CD3^{+}$, $CD4^{+}$, or $CD8^{+}$ T cells did not change significantly; however, there did appear to be a trend toward increased NK cell percentages post-laser injury (Figure 2E). Circulating cells followed a similar pattern with a trend toward increased circulating blood-borne NK cells present in the spleen 5 and 7 days post-laser, with no change in T cell subsets (Figure 2F). However, when we examined the proliferation and activation of NK cells and T cells in the spleen, we found that tissue-resident NK cells expressed significantly higher levels of proliferation marker Ki67 post laser administration, with a peak in proliferation at day 5 post-laser ($p = 0.0024$) (Figure 2G). $CD4^{+}$ and $CD8^{+}$ T cells showed increased proliferation at day 5 post-laser injury but to a much lesser extent than NK cells (Figures 2H and 2I). Tissue-resident splenic NK cells showed a sharp rise in CD69 expression, a costimulatory molecule that increases mTOR signaling and promotes migration and proliferation of NK cells, at day 3 post-laser injury, returning to baseline by days 5 and 7 (Figures 2J and S3C). This activation was also observed in $CD4^{+}$ and $CD8^{+}$ T cells (Figures 2K and 2L) but to a far lesser extent than NK cells. When circulating lymphocytes were examined, NK cells displayed increased Ki67 at days 3 and 5 (Figure 2M), while $CD4^{+}$ and $CD8^{+}$ T cells showed no proliferative change (Figures 2N and 2O). Circulating NK cells expressed significantly higher levels of CD69 at day 3 post-laser ($p = 0.0001$) (Figures 2P; S3D), with no change in T cells (Figures 2Q and 2R). Overall, retinal injury triggered a strong peripheral NK cell response, with minimal activation of $CD4^{+}$ or $CD8^{+}$ T cells.

Expansion of active NK cells in RPE/choroid tissue in response to retinal injury

NK cells have established roles in anti-tumor and anti-viral immunity but also play an important role in vascular remodeling

Figure 2. Peripheral splenic and circulating NK lymphocytes are activated by focal tissue injury to the retina

(A) Schematic of CNV induction in C57Bl/6J mice (8- to 10-week-old, 5/group); 3, 5, or 7 days post-CNV administration mice received an i.v. injection of anti-CD45PE to distinguish tissue-resident vs. circulating splenic lymphocytes.

(B) Representative gating for tissue-resident (tr) $CD45AF488^{+}CD45PE^{-}$ and circulating (c) $CD45AF488^{+}CD45PE^{+}$ lymphocytes; see also Figures S3A and S3B.

(C) Gating strategy for trNK and T cells.

(D) Gating strategy for circulating lymphocytes.

(E) $trCD45^{+}$ cells as a percentage of total $CD45^{+}$ cells, subdivided into trNK ($NK1.1^{+}CD3^{-}$), $trCD3^{+}$ T($NK1.1^{-}CD3^{+}$), $trCD4^{+}$ ($NK1.1^{-}CD3^{+}CD4^{+}CD8^{-}$), or $trCD8^{+}$ T cells ($NK1.1^{-}CD3^{+}CD4^{-}CD8^{+}$).

(F) $cCD45^{+}$ cells similarly subdivided into cNK, $cCD3^{+}$, $cCD4^{+}$, or $cCD8^{+}$ T cells.

(G) Ki67 expression on trNK cells, with representative gating.

(H and I) Ki67 expression on $trCD4^{+}$ and $trCD8^{+}$ T cells.

(J) CD69 expression on trNK cells, with representative gating; see also Figure S3C.

(K and L) CD69 expression on $trCD4^{+}$ and $trCD8^{+}$ T cells.

(M) Ki67 expression on cNK cells, with representative gating.

(N and O) Ki67 expression on $cCD4^{+}$ and $cCD8^{+}$ T cells.

(P) CD69 expression on cNK cells, with representative gating; see also Figure S3D.

(Q and R) CD69 expression on $cCD4^{+}$ and $cCD8^{+}$ T cells. Data presented as mean ± SEM. $n = 5$ mice per group. $^{*}p < 0.05$, $^{**}p < 0.01$, $^{***}p < 0.005$, $^{****}p < 0.001$. Following normality testing, one-way ANOVA with Dunnett's multiple comparison test was used to compare groups.

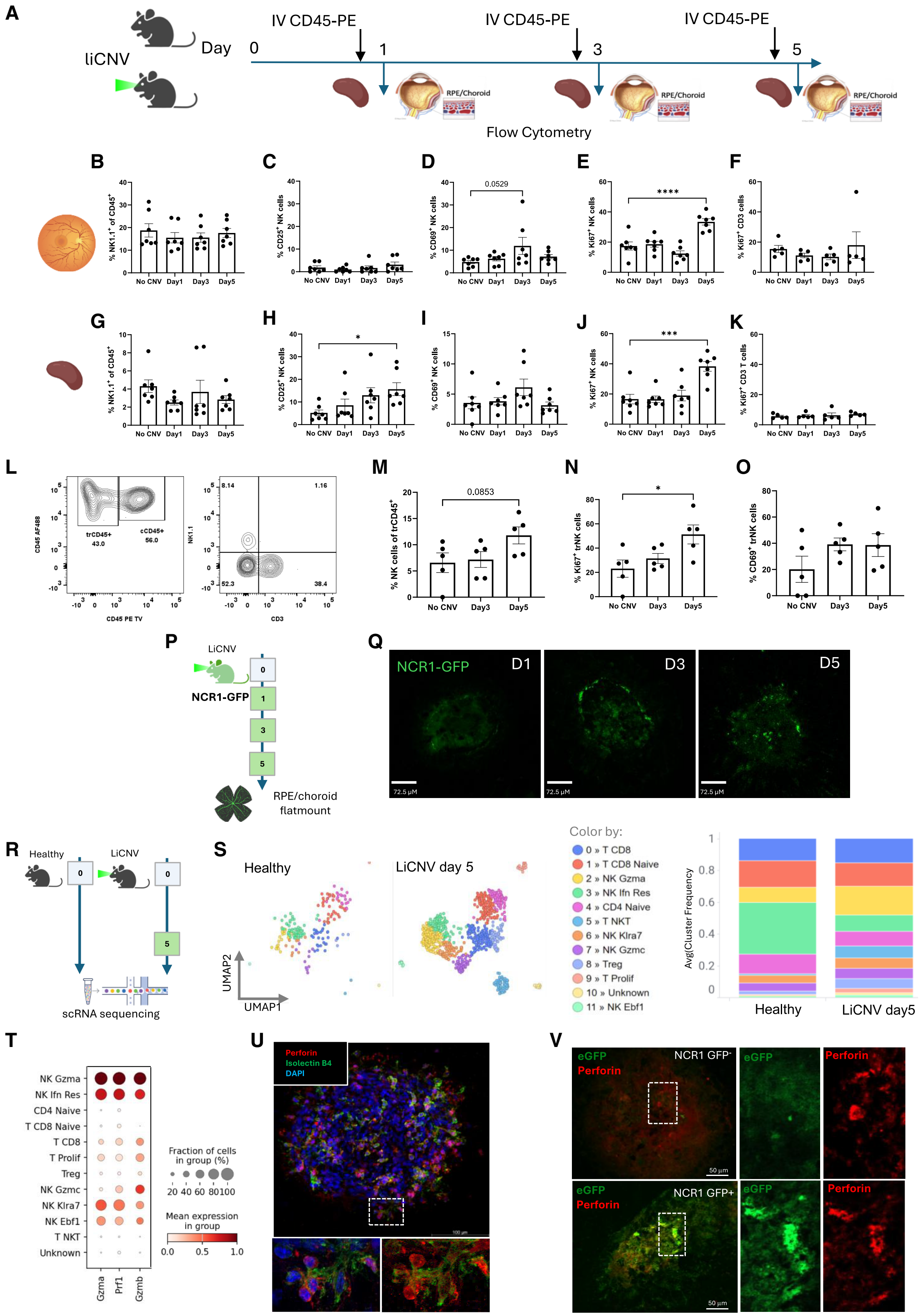

(legend on next page)

during early pregnancy.[17] Given the importance of vascular remodeling during AMD, we hypothesized that NK cells may be found at sites of vascular disease in AMD and models of nAMD. Using the liCNV model, we examined NK cell presence at choroidal neovascular lesions (Figure 3A). The numbers of NK1.1$^+$ NK cells as a percentage of CD45$^+$ lymphocytes did not change in the RPE/choroid following liCNV (Figures 3B and S3E), and we found no upregulation of CD25, a marker of proliferative potential[18] in the RPE/choroid tissue (Figure 3C). However, there was a trend toward increased CD69 expression post-liCNV (Figure 3D), with NK cells expressing significantly higher levels of Ki67 in the RPE/choroid at day 5 post-CNV (Figures 3E; S3F). This was directly contrasted by CD3$^+$ T cells where no increase in Ki67 expression was observed (Figure 3F).

In parallel, we phenotyped NK1.1$^+$ NK cells in the periphery following injury to the retina (Figure 3G). Splenic NK cells demonstrated increased activation 5 days post-CNV with significantly increased CD25 compared to animals with no liCNV (Figure 3H), a trend toward increased CD69 expression (Figure 3I) and a significant increase in Ki67 expression at day 5 post-CNV (Figure 3J). In contrast, splenic CD3$^+$ T cells showed no increase in Ki67 following retinal injury (Figure 3K). Using tail vein injection of anti-CD45 PE, we discriminated tissue-resident (tr) from circulating lymphocytes in the RPE/choroid tissue of C57Bl/6J mice who had received liCNV. We observed a clear signature of trNK cell proliferation with significantly higher levels of Ki67 in trNK cells post-liCNV compared to controls, with trends toward increasing trNK cell numbers and increased expression of CD69 in trNK cells (Figures 3L–3O and S3G). To better determine the appearance of NK cells in the RPE/choroid, we performed liCNV on NCR1-GFP$^+$ mice[19] and imaged RPE/choroid flatmounts at day 1, 3, and 5 post-injury (Figure 3P). GFP$^+$ NK cells infiltrated the lesion at days 3 and 5 post-liCNV (Figure 3Q). Control experiments were performed on NCR1-GFP$^-$ mice, and representative RPE/choroid flatmounts are shown in Figure S3H.

Increased presence of cytolytic NK cells at lesion site following retinal injury

The consistent proliferative profile of NK cells responding to retinal injury and appearance of GFP$^+$ NK cells at the liCNV strongly imply NK cells are a key factor in the immune response to retinal pathology, at least in the context of neovascular disease. NK cells mediate their regulatory functions through multiple mechanisms, including cytolytic killing of target cells through granzyme and perforin and modulation of the immune environment through secretion of IFN-γ. To identify dominant NK cell phenotypes in liCNV RPE/choroid tissue, we utilized a single-cell RNA sequencing (scRNA-seq) dataset comparing healthy and day 5 post-liCNV samples (Figure 3R). We performed subcluster analysis of the T/NK cell cluster (Figures 3S; S4A) and identified 12 distinct lymphocyte clusters. Following liCNV injury, there was a marked expansion in *GranzymeA*-expressing NK cells (NK Gzma) along with a commensurate contraction of the IFN subset of NK cells (NK IFN res). Most T cell populations remained stable between healthy versus liCNV mice, apart from a notable increase in Treg cells (Figure 3S). Gene expression analysis showed high levels of *Perforin* specifically in NK cell clusters, but not in T cell clusters, in this tissue isolate (Figure 3T), and immunofluorescent histochemistry of matching RPE/choroid flatmounts 5 -days post-liCNV confirmed strong perforin protein staining at the CNV site, indicating the presence of cytotoxic NK cells (Figures 3U and S4B). High-magnification micrographs highlight perforin$^+$ cells at sites of isolectin B4$^+$ (IB4) neovascular sprouts in C57Bl/6J mice following liCNV. Immunofluorescent histochemistry of RPE/choroid flatmounts from NCR1-GFP$^-$ and NCR1-GFP$^+$ confirmed perforin$^+$ NK cells at the neovascular lesion site (Figure 3V).

Figure 3. Expansion of active, cytolytic NK cells at lesion site in response to retinal injury
(A) Schematic of CNV induction in C57Bl/6J mice (8- to 10-week-old, 5–7 per group).
(B) NK1.1$^+$ NK cells expressed as a percentage of CD45$^+$ lymphocytes in the RPE/choroid.
(C–E) (C) CD25, (D) CD69, and (E) Ki67 on RPE/choroid NK1.1$^+$ NK cells; see also Figure S3F.
(F) Ki67 expression on CD3$^+$ T cells within the RPE/choroid.
(G) NK1.1$^+$ NK cells expressed as a percentage of CD45$^+$ splenic lymphocytes.
(H–J) CD25, CD69, and Ki67 expression on splenic NK1.1$^+$ NK cells.
(K) Ki67 expression on CD3$^+$ T cells within the spleen.
(L) Gating strategy for circulating lymphocytic cells and NK1.1$^+$ NK cells in the RPE/choroid (n = 5).
(M) NK1.1$^+$ NK cells as a percentage of trCD45$^+$ lymphocytes in the RPE/choroid.
(N and O) Ki67 and CD69 expression on RPE/choroid trNK cells; see also Figure S3G.
(P) Schematic of CNV induction and analysis in NCR1-GFP$^+$ mice (8- to 10-week-old, n = 4–6 eyes/time point, 2–3 CNV/eye).
(Q) Representative immunofluorescent staining of RPE/choroid flatmount of liCNV in NCR1-GFP$^+$ mouse (n = 4–6 eyes/time point, 2–3 CNV/eye); see also Figure S3H. Scale bar: 72.5 μm.
(R) Schematic of scRNA-seq analysis on RPE/choroid of control or mice 5 days following laser-induced CNV administration (C57Bl/6J 12-16-week-old males, n = 8 eyes/group).
(S) UMAP and clustering analysis of cells in the RPE/choroid of healthy vs. day 5 post-liCNV from C57BL/6J mice (n = 8 eyes, ≥3 lesions/eye for liCNV eyes).
(T) Dot plot showing expression pattern of *GranzymeA*, *Perforin*, and *GranzymeB* across cell types. Dot color indicates average expression level within each group. Dot size indicates fraction of cells/group that express a particular gene; see also Figure S4A.
(U) Representative immunofluorescent staining for perforin and isolectin in RPE/choroid flatmount of liCNV (n = 6 eyes, 2–3 liCNV/eye). Scale bar: 100 μm.
(V) Representative immunofluorescent staining for perforin in RPE/choroid flatmount of liCNV in NCR1-GFP$^+$ mice (n = 4 eyes, 2–3 liCNVs/eye); see also Figure S4B. Scale bar: 50 μm. Graphical data presented as mean ± SEM; n = 5–7 animals/group. *p < 0.05, ***p < 0.005, ****p < 0.001. Following normality testing, one-way ANOVA with Dunnett's multiple comparison test was used to compare groups.

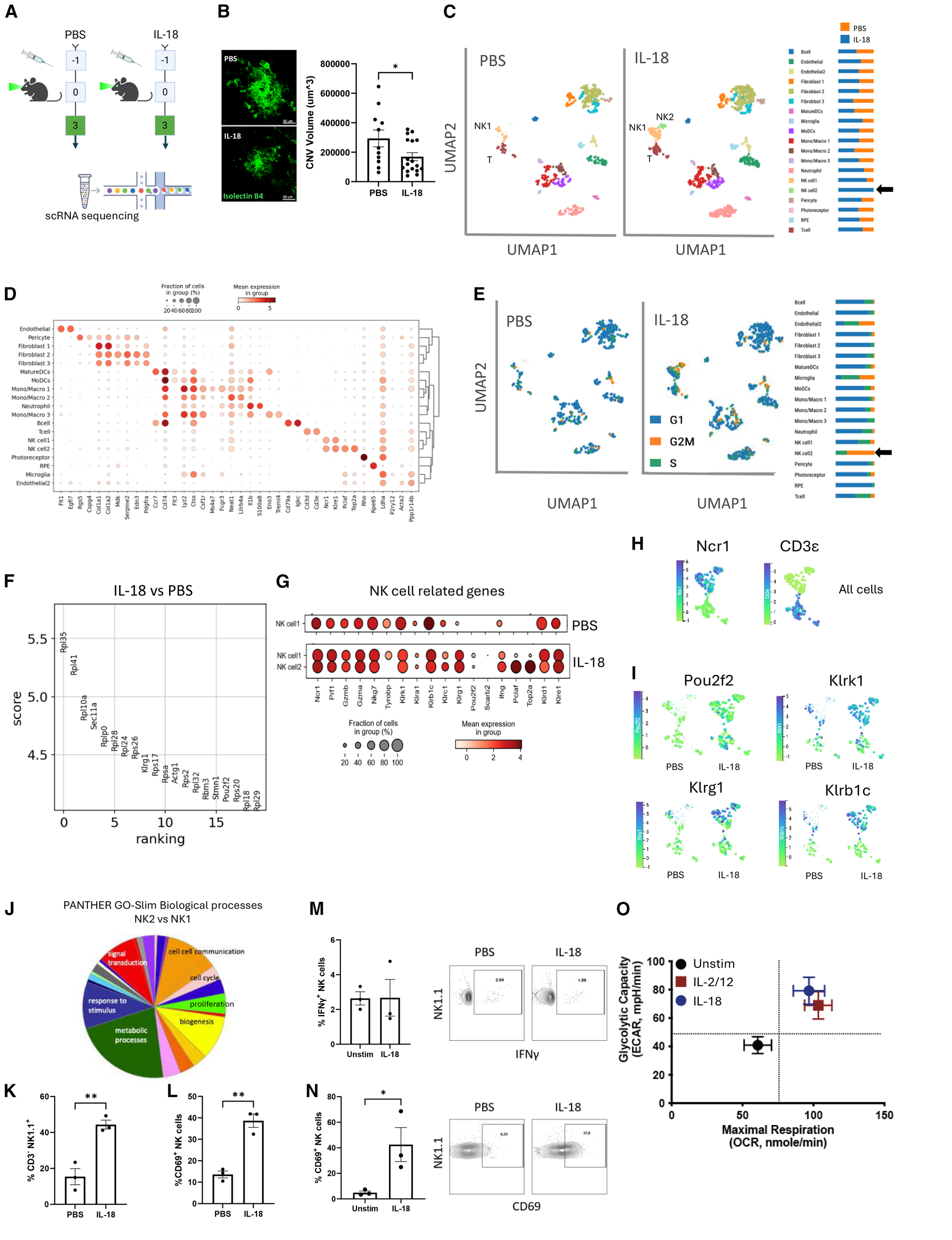

(legend on next page)

NK cell proliferation, biogenesis, and metabolic activity are heightened during active resolution of choroidal neovascular lesions

Our data pointed to NK cells as highly responsive to retinal neovascular pathology. Previous work from our group has shown that rIL-18 mediates the resolution of CNVs in mice and non-human primates.[20–22] Interestingly, NK cells respond strongly to several cytokines including IL-18, indicating that activated NK cells may mediate IL-18-driven resolution of neovascularization. To examine this, we administered intraperitoneal PBS or IL-18 in conjunction with administration of an liCNV (Figure 4A) and carried out scRNA-seq on RPE/choroid tissue surrounding the neovascular lesions (Figure S5A). Resolution of CNV volume in rIL-18-treated mice was visualized by IB4 labeling of endothelial cells (ECs) on RPE/choroid flatmounts 7 days post-liCNV (Figure 4B). We identified an increase in number of NK cells in resolving CNV compared to active CNV, i.e., with IL-18 treatment vs. PBS treatment, from our scRNA-seq dataset. One population, "NK1," was present in both models but increased with IL-18 treatment (6.9% of cells) compared to PBS (2.8% of cells), whereas a second population, termed NK2, was present only in the resolving model (2.7% of all cells) (Figure 4C). Gene expression analysis showed that along with expression of classical NK markers *Ncr1* and *Klre1*, the NK2 subset was enriched for genes essential for processes involved in cell proliferation (Figure 4D). When cell-cycle status was analyzed, the NK2 subset found in CNV lesions from IL-18-treated mice was found predominantly in the G2M and S phase of the cell cycle (Figure 4E) compared to PBS-treated mice. Ranking analysis of the top 20 differentially expressed genes (DEGs) in NK cells in liCNV tissue from IL-18- versus PBS-treated mice showed upregulation of several genes for ribosomal proteins, critical in mediating protein synthesis (Figure 4F). In fact, we observed upregulation of several genes in the NK2 cell cluster indicative of a highly active, proliferative NK cell phenotype including *Klrg1*, *Pclaf*, and *Top2a* (Figures 4G; S5B). Further analysis identified specific genes associated with increased NK cell metabolic and cytolytic activity. NK and T cells could be distinguished by their expression of *Ncr1* and *CD3ε* within the T/NK cell clusters from PBS- and IL-18-treated mice (Figure 4H). *Pou2f2*, involved in glycolytic reprogramming of lymphocytes,[23] was increased in neovascular lesions of IL-18-treated mice, along with *Klrg1*, an immune checkpoint receptor, indicative of a metabolically active environment. NK-cell-activating receptors *Klrk1* and *Klrb1c* involved in increased cytolytic activity were also significantly upregulated in lesional tissue of IL-18-treated mice compared to PBS controls (Figure 4I). These NK cells also showed increased gene expression of *Perforin*, *GranzymeA*, and G*ranzymeB* (Figures S5C–S5E), essential effector molecules in NK cytolysis of target cells. Enhanced NK cell activity in resolving lesions is further supported by PANTHER GO-Slim analysis comparing DEGs between NK2 vs. NK1 cells, demonstrating overrepresentation of specific gene sets involved in metabolic processes and biogenesis in the NK2 population (Figure 4J) (full list of gene processes in Figure S6A).

The increased presence of NK cells in neovascular tissue in response to IL-18 in the scRNA-seq dataset was confirmed by flow cytometry (Figure S6B). NK1.1$^+$ cells made up over 40% of the CD45$^+$ leukocyte population in CNV tissue isolated from mice treated with IL-18, double that present in vehicle control (Figure 4K). Furthermore, these NK cells found at resolving lesions showed enhanced expression of activation marker CD69, indicative of heightened metabolic activity (Figure 4L).[24–26]

NK cells are commonly treated with IL-18 in combination with IL-12 or IL-15, because IL-18 alone does not drive IFN-γ secretion from NK cells. We confirmed that *in vitro* IL-18 treatment of isolated naive murine splenocytes did not drive IFN-γ secretion (Figure 4M) but did increase CD69 expression (Figure 4N). Our scRNA-seq and flow cytometry data indicated IL-18 rewires NK cells for population expansion, priming their metabolic activity. We confirmed this through Seahorse XF Cell Energy Phenotype Stress Test analysis. NK cells isolated from mouse splenocytes treated with IL-18 alone were metabolically rewired to take on a highly energetic phenotype, similar to IL-2/IL-12, increasing both glycolytic and mitochondrial metabolism (Figure 4O).

Figure 4. NK cell proliferation, biogenesis, and metabolic respiration are heightened during active resolution of neovascular lesions

(A) scRNA-seq experimental design for 12- to 16-week-old wild-type male mice treated intraperitoneally (i.p.) with PBS or IL-18 and subjected to laser-induced CNV (n = 4 mice/group, ≥3 lesions/eye). Scale bar: 50 μm.

(B) Isolectin B4 analysis of CNV size in RPE/choroid flatmounts (n = 11 PBS; n = 17 IL-18 [lesions]).

(C) UMAP (Scanpy) of CNV tissue from PBS- and IL-18-treated mice (n = 8 eyes/condition). Bar chart shows treatment-specific cell-type proportions; see also Figure S5A.

(D) Dot plot of known marker genes across cell types. Dot size indicates proportion of cells in cluster expressing a gene; color indicates relative level of expression.

(E) UMAP showing predicted cell-cycle phase with summary bar chart of phase distribution per cell type.

(F) Top 20 DEGs in NK cells (IL-18 vs. PBS), ranked by Wilcoxon significance; see also Figure S5B.

(G) Dot plot of NK-related genes in NK1 and NK2 populations under PBS or IL-18 treatment.

(H) *Ncr1* and *CD3ε* expression in cells from RPE/choroid of mice (n = 8 eyes).

(I) *Pou2f2*, *Klrg1, Klrk1*, and *Klrb1c* gene expression in PBS- versus IL-18-treated mice.

(J) Functional enrichment analysis of genes differentially upregulated in NK2 vs. NK1 cells in IL-18-treated samples only; see also Figure S6A.

(K and L) Flow cytometry analysis of CNV tissue from 8- to 10-week-old C57Bl/6J mice treated with PBS or IL-18 (n = 3 mice/group); see Figure S6B for gating.

(M and N) Flow cytometry of CD69 and IFN-γ expression in splenocytes from naive C57Bl/6J mice (8–10 week old) treated *ex vivo* with PBS or rIL-18 (three mice per group); see Figure S6C.

(O) Seahorse analysis of energetic phenotype of NK cells isolated from C57Bl/6J mouse splenocytes unstimulated vs. IL-2/IL-12 pair vs. IL-18 alone. Bar charts presented as mean ± SEM. *$p < 0.05$, **$p < 0.01$. Following normality testing, unpaired t tests or one-way ANOVA with Dunnett's multiple comparison tests were used to compare groups.

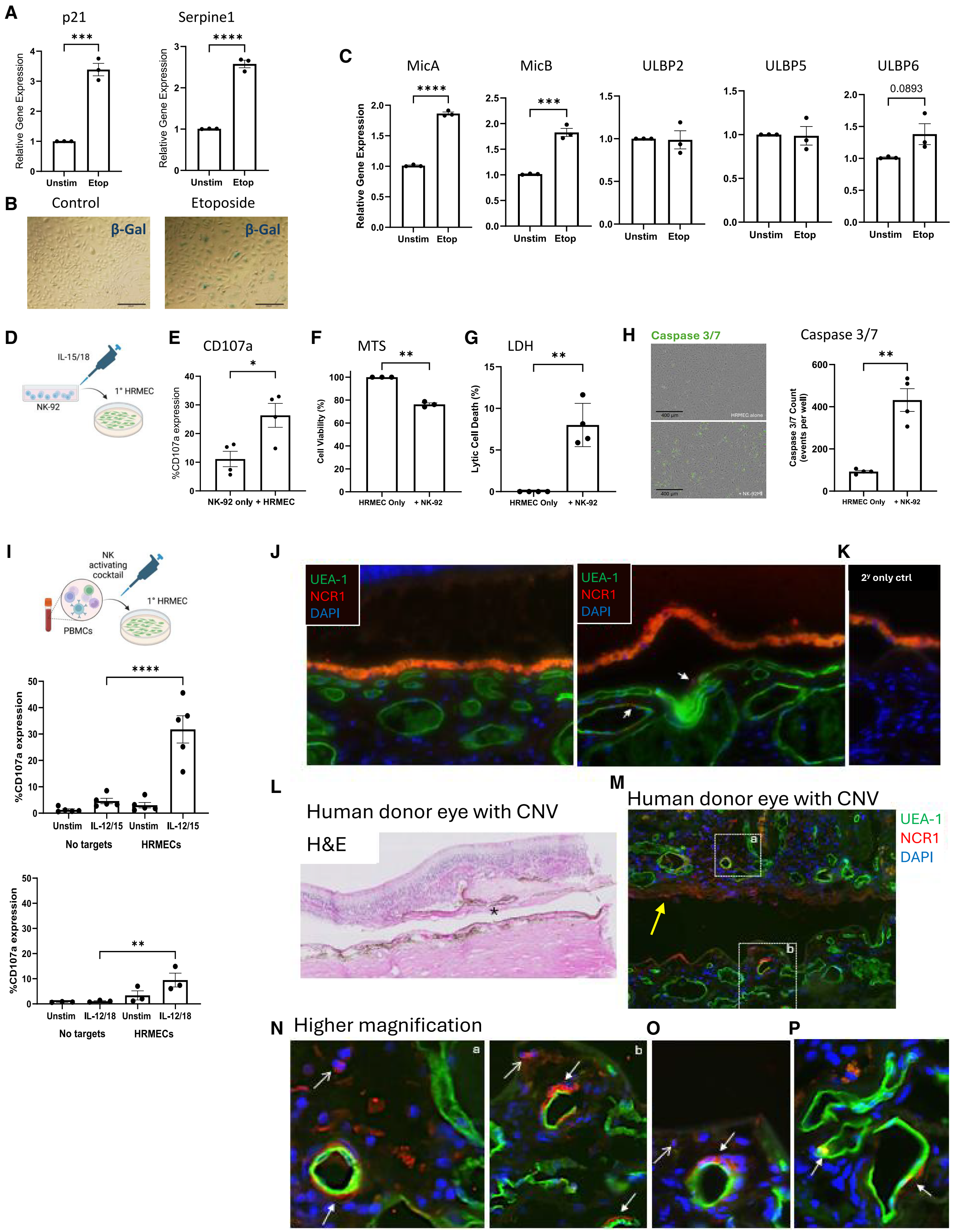

(legend on next page)

Endothelial cells can act as targets for activated NK cells

NK cell activation is regulated by a balance of signals from host cells using various inhibitory and activatory receptors. One-way NK cytolytic activity is induced through upregulation of NKG2D ligands (NKG2DL) on target cells. A variety of stressors such as oxidative stress, cancer, viral infection, or senescence can increase the expression of NKG2DL (MICA/B and ULBP1-6), which, when recognized by NKG2D on NK cells, stimulate directed cytolytic activity toward the stressed or damaged target cell.[27–31] Given the proximal localization of perforin$^+$ and IB4$^+$ cells in murine CNV tissue (Figure 3U), coupled with the previously reported ability of NK cells to target porcine ECs,[32] we investigated whether primary human retinal microvascular endothelial cells (HRMECs) could act as targets for NK cells in the RPE/choroid. Real-time qPCR was used to investigate levels of NKG2DL mRNA in HRMECs following treatment with senescence-inducing agent etoposide. Senescence in HRMECs was confirmed by measuring increased p21 and Serpine1 expression and accumulation of the hydrolase enzyme beta-galactosidase (β-gal) (Figures 5A and 5B). In parallel, MICA/B and ULBP6 levels were increased in etoposide-treated HRMECs, indicating senescent HRMECs as potential target cells for NK cell killing (Figure 5C). Together, these data support a mechanism whereby retinal ECs have potential to act as target cells for NK cell cytolysis in nAMD.

NK cells can sometimes compensate for low NKG2DL expression through cytokine activation, so we tested whether cytokine-activated NK cells could kill ECs. Because IL-12, IL-15, and IL-18 each shape NK cell function differently,[33,34] and IL-15/-18 together drive proliferation and cytotoxicity with increased Prf1, Lamp1, and key NK receptors,[33,35,36] we selected IL-15/18 to generate highly cytotoxic, proliferative NK cells. We found activated NK cells can target primary HRMECs using a human NK92MI cell line, stimulated with IL-15/-18. CD107a, a marker of NK degranulation was upregulated on NK92MI cells in response to co-culture with HRMECs (Figures 5D and 5E). In parallel, we performed several viability assays on HRMECs following NK92MI incubation. HRMECs incubated with NK92MI cells had decreased cell viability (Figure 5F) and increased lytic cell death (Figure 5G). HRMECs incubated with NK92MI cells showed increased expression of caspase-3/-7, an apoptotic marker (Figure 5H).

To further characterize the ability of NK cells to target ECs, PBMCs from healthy donors were treated with IL-12/-15 or IL-12/-18 NK-activating cytokine cocktails and co-cultured with HRMECs. Activation with IL-12/-15 or IL-12/-18 was sufficient to induce CD107a expression on NK cells when incubated with HRMECs (Figure 5I), while addition of HRMECs had little effect on cytokine-induced IFN-γ production by NK cells (Figure S7B).

NK cells are found localized in tissue adjacent to neovessels in CNV lesions in human disease

Our evidence of lymphocyte activity in AMD, coupled with our observation of activated NK cells in a murine model of CNV, suggested NK cells would be found at neovascular lesions in human AMD donor eyes. We undertook natural cytotoxicity triggering receptor 1 (NCR1) labeling in human donor eye tissue from healthy individuals ($n = 3$) and individuals with CNV secondary to AMD ($n = 3$), Figure S7D. We observed occasional, scarce NCR1-labeled NK cells in the extravascular tissue in the choroid in human donor eye tissue from two of three healthy donors (Figure 5J left and middle panels, closed white arrows), with no NCR1-labeled cells detected in the subretinal space (control staining in Figure 5K). In contrast, we observed NCR1$^+$ NK cells in the neovascular lesion in human donor eyes with CNV (Figures 5L and M). NCR1$^+$ cells were found in the subretinal fibrovascular matrix (Figure 5M, yellow arrow) and the extravascular tissue (Figure 5N, high magnification, open white arrows). Clustering of NCR1$^+$ NK cells around UEA-I-labeled blood vessels was striking (Figure 5N, high magnification, closed arrows) and was observed in both subretinal space and choroid (Figure 5N A and B, respectively). NCR1$^+$ NK cells surrounding UEA-I-labeled blood vessels in CNV tissue from additional AMD donors were also observed (Figures 5O and 5P).

High NK cell cytotoxicity effector function but decreased proliferative capacity in AMD

Our data pointed to a reparative role for NK cells in CNV, potentially through the cytolytic clearance of proliferating ECs. Therefore, we wondered if NK cell cytolytic capacity was dysfunctional in human nAMD. PBMCs isolated from nAMD donors or

Figure 5. NK cells are found localized adjacent to neovessels in nAMD

(A) HRMEC cells were treated for 24 h with 100 μM etoposide and analyzed for expression of markers of cellular senescence ($n = 3$).

(B) Representative image of β-galactosidase expression in control versus etoposide-treated HRMECs ($n = 3$). Scale bar: 200 μm.

(C) NKG2DL expression on HRMECs treated with etoposide ($n = 3$); see primer list in Figure S7A.

(D–H) NK92MI cells were treated for 18 h with IL-15/18 and incubated with or without HRMECs for 4 h, scale bar: 400 μm, (E) expression of CD107a measured by flow cytometry ($n = 4$), (F) cell viability measured by MTS assay ($n = 3$), (G) lytic cell death measured by LDH assay ($n = 4$), and (H) target cell apoptosis measured by caspase-3/-7 assay ($n = 4$).

(I) PBMCs were isolated from healthy donors, treated with NK-activating cytokines (IL-12/15 or IL-15/18) for 18 h, and incubated with or without HRMECs for 4 h. Expression of CD107a was measured by flow cytometry ($n = 3$–5); see also Figure S7B.

(J and K) Representative and control immunostaining of NCR1 (red) and UEA-1 (green) in cross-sections from non-disease donor controls ($n = 3$); see donor information in Figure S7D.

(L) H&E staining in human donor eye with CNV. Orange autofluorescence due to RPE lipofuscin. *denotes area of CNV.

(M–P) Representative immunostaining of NCR1 (red) and UEA-1 (green) in cross-sections of patients with nAMD ($n = 3$); higher magnifications of lesional tissue of one donor shown in Na&b. High-magnification images of two further donors shown in (O) and (M). Closed white arrows denote areas of NCR1$^+$ staining at or adjacent to blood vessels. Open white arrows denote areas of NCR1$^+$ staining in choroidal tissue. Yellow arrows denote areas of NCR1$^+$ staining at the neovascular membrane. Graphical data presented as mean ± SEM. *$p < 0.05$ **$p < 0.01$, ***$p < 0.005$, ****$p < 0.001$. Following normality testing, unpaired t tests or one-way ANOVA with Dunnett's multiple comparison tests were used to compare groups.

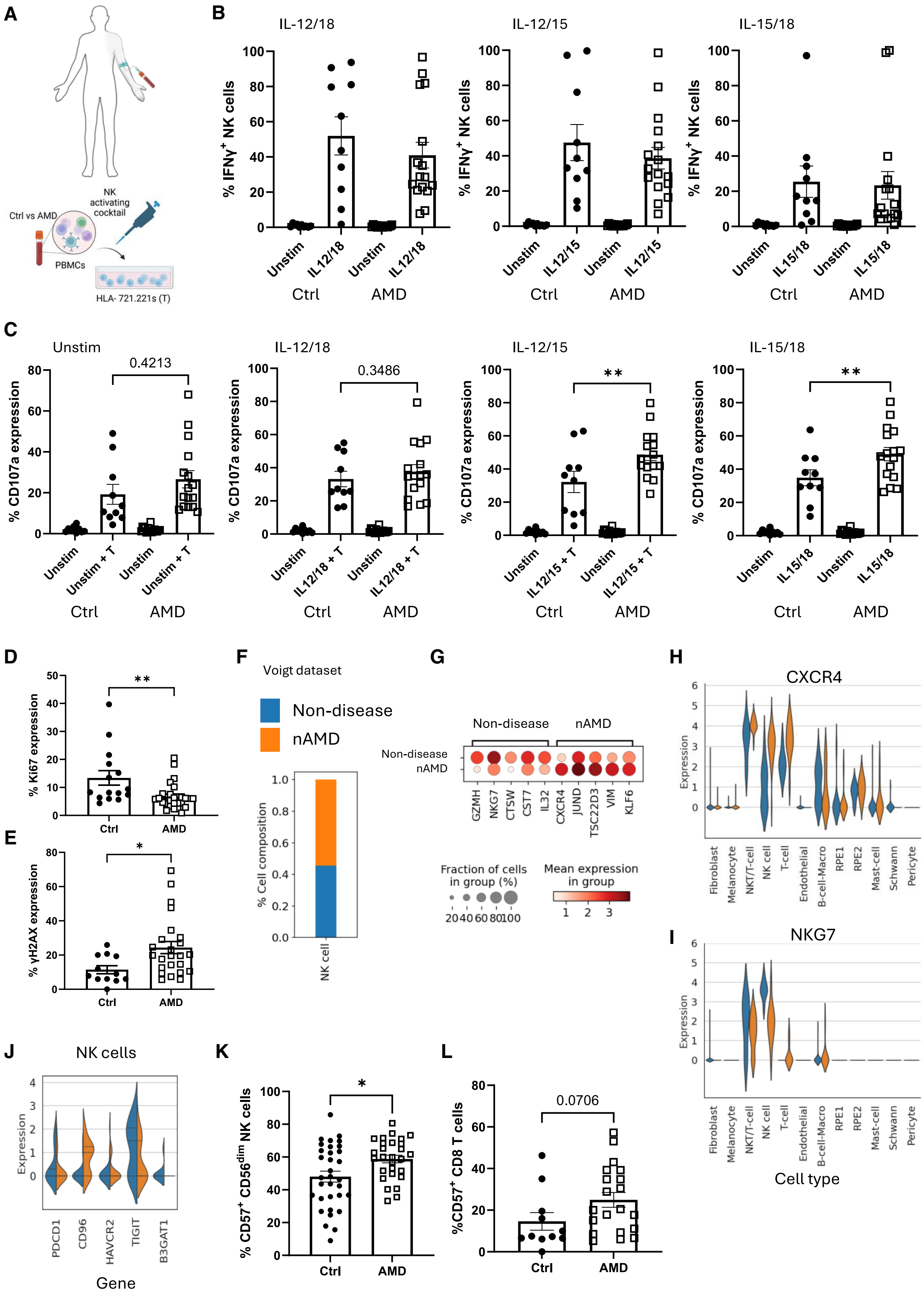

(legend on next page)

age-matched healthy donors were incubated with NK-cell-activating cytokine cocktails prior to incubation with HLA-negative target cells (721.221s) (Figure 6A). Following stimulation, peripheral NK cells from nAMD donors made equivalent levels of IFN-γ to age-matched controls (Figure 6B). However, CD107a expression was increased on NK cells when IL-12/-15 or IL-12/-18-treated PBMCs were incubated with target cells (Figure 6C).

One key feature in our mouse model of resolving liCNV was the presence of metabolically active, proliferating NK cells. Therefore, we investigated the proliferative capacity of NK cells in peripheral blood of nAMD donors and found significantly lower expression of Ki67 on NK cells in nAMD patients (Figure 6D). A major reason for cells withdrawing from the cell cycle in age-related disease is due to DNA damage. We found that NK cells from nAMD donors have significantly higher levels of γH2Ax, a marker of DNA damage, than NK cells from healthy age-matched donors (Figure 6E). Together, these data indicate that peripheral NK cells retain cytolytic potential in AMD but fail to proliferate at the injury site, potentially contributing to the failure to resolve neovascular lesions. To investigate whether these observed changes in peripheral NK cells from human nAMD donors were present closer to the site of disease, we examined publicly available scRNA-seq data acquired from human retinal tissue.[12] We performed clustering analysis of a donor with nAMD and a donor with no disease (Figure S8A) and found similar numbers of NK cells as a percentage of the total cell composition between tissues of non-diseased and nAMD donors (Figure 6F), matching our data from peripheral blood of donors with AMD (Figure 1G), likely due to the site of tissue collection—peripheral to the lesion site. When we analyzed the top five DEGs between non-diseased and nAMD donors, we observed striking differences in NK cell activation markers *NKG7* and *CXCR4* (Figures 6G and S8B). To determine specificity, we examined *CXCR4* and *NKG7* expression across all cell clusters and found expression was well matched across most cell types, with the greatest differential in the NK cell cluster (Figures 6H and 6I). Because CXCR4 signaling has been linked to NK cell quiescence in tumors,[37] we assessed markers of quiescence or functional exhaustion in NK cells in nAMD versus healthy retinal tissue. Among several immune checkpoint receptors examined, CD96 emerged as a highly differentially expressed gene in nAMD ($\log_2$FC = 3.14; p-adj = 0.003), which, together with increased CXCR4 expression, is indicative of an exhausted NK cell phenotype in AMD donor tissue (Figure 6J). CD57, a marker of maturity associated with highly cytotoxic but poorly proliferative $CD16^+CD56^{dim}$ NK cells[38] and with homing to inflamed peripheral tissues,[39] was also elevated in peripheral blood of nAMD patients versus age-matched healthy controls (p = 0.0110) (Figure 6K). No differences in CD57 expression were detected on $CD56^{bright}$ NK cells or $CD4^+$ (Figures S9A and S9B) or $CD8^+$ T cells, though there was an expected trend toward increased age-related exhaustion in $CD8^+$ T cells (p = 0.0706) (Figure 6L). The presence of more $CD56^{dim}CD57^+$ NK cells in AMD donors indicates a smaller pool of NK cells with proliferative capacity in the periphery in AMD.

Adoptive transfer of activated NK cells reduces pathological neovascularization and repairs barrier integrity

In human disease, circulating NK cells show reduced proliferative capacity, while tissue-infiltrating NK cells display inhibitory signals in the retina. In mice, peripheral NK cells respond to retinal injury with a proliferative, metabolically active phenotype that supports lesion resolution. IL-15 has been used to enhance the proliferation and activation of NK cells and to overcome CXCR4-induced NK cell quiescence in non-ocular disease. We hypothesized that systemic introduction of cytokine-activated NK cells would home to sites of retinal injury. To test this, GFP^- or GFP^+ NK cells were isolated from NCR1-GFP mice, activated with IL-18/IL-15/IL-12, and injected intraperitoneally into C57Bl/6J mice on the day of laser-induced injury. By day 3 post-liCNV, GFP^+NK cells were detectable in the RPE/choroid at the lesion site (Figures 7A and 7B, lower panel).

To assess if adoptive transfer of cytokine-activated NK cells could enhance resolution of CNV lesions, NK cells from C57Bl/6J splenocytes were isolated, activated with IL-18/IL-15/IL-12, and injected intraperitoneally into C57Bl/6J mice on the same day as laser-induced injury (Figure 7C). Providing activated NK cells into the periphery clearly enhanced resolution of liCNV, significantly decreasing volume size of CNVs (p = 0.0129) (Figure 7D). We repeated this adoptive NK cell transfer in a second mouse model of spontaneous retinal neovascularization, JR5558-model, using cytokine-activated allogenic NK cells (Figure 7E). IL-12 primarily induces the secretion of

Figure 6. Metabolic quiescence is observed in NK cells in nAMD

(A) PBMCs were isolated from age-matched control donors (n = 10) or donors with nAMD (n = 16), treated with NK-activating cytokine cocktails for 18 h, and subsequently incubated with or without HLA^- 721.221 target cells for 4 h.

(B and C) IFN-γ and CD107a expression on NK cells was measured by flow cytometry.

(D and E) Flow cytometric analysis of Ki67 expression (D) or γH2Ax on NK cells (E) from control ($n \geq 12$) versus AMD donor ($n \geq 24$) PBMCs; see also Figures S9A and S9B.

(F–J) Reanalysis of scRNA-seq dataset from Voigt et al.[12]; see also Figure S8. (F) Stacked bar chart of NK cell group from scRNA-seq of 1,237 cells derived from peripheral ocular tissue from two human donors (non-disease and nAMD) showing the percentage cell composition comprised of each donor (non-disease: 45%; nAMD: 55%). (G) Dot plot showing the top 10 DEGs between non-disease and nAMD donor in NK cell cluster. Dot color indicates average expression level within each donor, and size indicates fraction of cells in each group expressing a particular gene. (H and I) Violin plots representing expression of CXCR4 and NKG7 across cell types and between donors (blue = non-disease, orange = nAMD). (J) Violin plot representing markers of exhaustion or terminal differentiation in NK cells between donors.

(K) CD57 expression on $CD56^{dim}$ NK cells (n = 26 Ctrl, n = 37 AMD) and (L) $CD8^+$ T cells (n = 11 Ctrl, n = 20 AMD); see also Figures S9C and S9D. Bar charts presented as mean ± SEM. *$p < 0.05$, **$p < 0.01$. Following normality testing, unpaired t tests or one-way ANOVA with Dunnett's multiple comparison tests were used to compare groups.

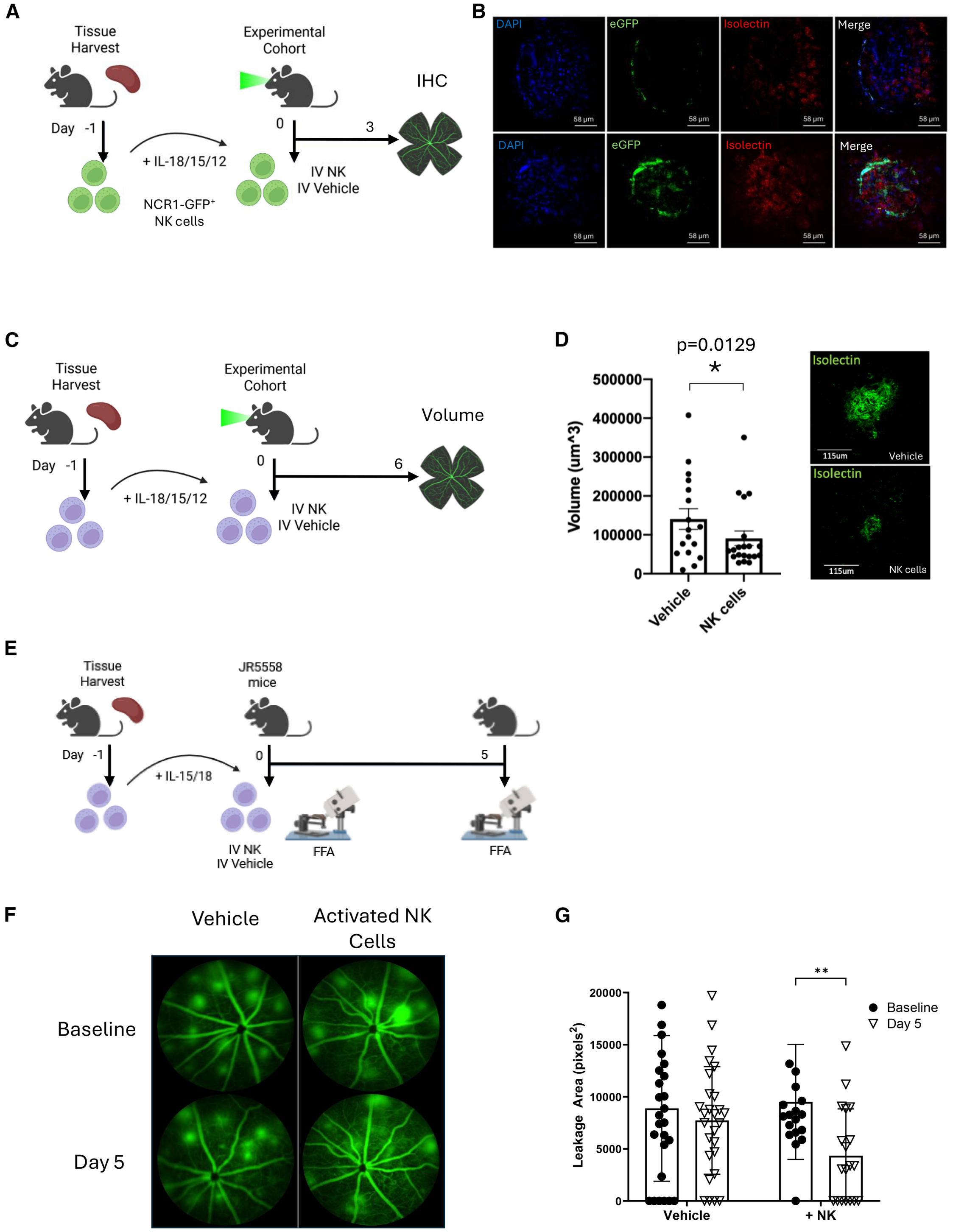

Figure 7. NK cell immunotherapy reduces neovascular lesion volume and repairs neovessel barrier integrity in models of retinal neovascularization

(A) Schematic representation of therapeutic administration of isolated, activated NCR1-GFP^{-} or NCR1-GFP^{+} NK cells in liCNV model in C57Bl/6J mice.

(B) RPE/choroid flatmounts from wild-type mice receiving adoptive transfer of IL-12/-15/-18-activated NCR1-GFP^{-} NK cells (top panel) or NCR1-GFP^{+} NK cells were stained for isolectin B4 (red) and DAPI (blue) at 3 days post-liCNV (n = 4 eyes, 3–4 lesions/eye). Scale bar: 58 µm.

(C and D) Schematic representation of therapeutic administration of isolated, activated NK cells in the liCNV model. (D) RPE/choroid flatmounts were stained for isolectin B4 (green) and CNV volume was analyzed by confocal microscopy and IMARIS. n = 5 mice/group, 3 CNVs/eye. Scale bar: 115 µm.

(legend continued on next page)

IFN-γ following activation of NK cells.[33] Given our data indicating the importance of NK cell cytotoxicity over IFN-γ secretion,[40] we tested the ability of IL-15/IL-18-activated NK cells, in the absence of IL-12, in this spontaneous model of neovascularization. Using fundus fluorescein angiography to visualize the permeability of the neovascular lesions *in vivo*, we found that providing cytokine-activated NK cells into the periphery could enhance vessel integrity (Figure 7F), significantly reducing vascular permeability (Figure 7G). This illustrates that cytokine-activated NK cells migrate from the periphery to the site of retinal injury and that immunotherapy in the form of adoptive transfer of cytokine-activated NK cells drives resolution of pathological neovascularization.

DISCUSSION

The number of people with AMD has reached 196 million, with an estimated 1 in 10 people over 55 years already showing early signs of the condition.[41] Seventy percent of those who progress to late-stage disease will develop nAMD due to uncontrolled growth of blood vessels beneath the retina.[2] Underlying mechanisms of disease remain elusive. In this study, we identified an imbalance in type 1 vs. type 2 immune signatures, in an unbiased, large human population cohort study, with AMD status associating with diminished type 2 immunity and enhanced type 1 immunity. Enhanced type 1 immune function, as evidenced by a trend toward increased IFN-γ was observed when all AMD grades were combined and became significant after disease onset in early severe disease. While diminished type 2 immunity, as evidenced by decreased IL-4 level, was observed in early moderate disease, implying that IL-4 variation precedes IFN-γ variation and potentially that IL-4 loss enables IFN-γ induction. Our first key finding in this study is that cytokine variation only occurs in the intermediate stages of disease, indicating that the cytokine imbalance is likely the result of a response to disease rather than being an initiator of disease. This also explains why we see no differences in IFN-γ production following stimulation of isolated NK cells from nAMD versus age-matched control study participants, despite observing increased IFN-γ in the plasma of AMD participants. Together, these data suggest the capacity of NK cells to make IFN-γ is not causative of retinal pathology but an adaptive response to pathology.

Changes in NK cell subsets are a feature of normal aging, with an increase in total NK cells and overrepresentation of the more mature CD56^{dim} cells in the periphery.[42,43] However, little is known about the phenotype and function of NK cells in AMD. A major finding of this study is that NK cell populations isolated from peripheral blood are significantly different between people with AMD and an age-matched cohort with no AMD. In striking contrast, T cell populations showed no significant differences between AMD and non-AMD cohorts. Although the overall numbers of NK cells within the lymphocyte population are similar between AMD and non-disease cohorts, there is an enrichment in CD56^{dim} NK cells in the peripheral blood of AMD patients compared with non-disease cohort. Similar data reported for other non-ocular diseases indicate a shift in CD56 expression coincides with alterations in NK cell function and may indicate that people with AMD have NK cells less capable of adapting to cellular stress.[44]

While nAMD is a chronic disease, we can recapitulate many aspects of human neovascular disease using an acute liCNV model of retinal damage in mice. Using liCNV, as a model for reverse-translation of this phenotype, we confirmed that localized retinal injury leads to a systemic activation of splenic NK cells, supporting our hypothesis that peripheral NK cells respond directly to retinal pathology.

Recently, Dong et al. also observed a restricting role for NK cells in resolution of liCNV, finding that NK cells promote neutrophil extracellular traps to mediate their effect.[11] Neutrophil extracellular traps had previously been reported to contribute to vascular remodeling in the retina during oxygen induced retinopathy.[45] An important advance of our study is the finding that NK cells at the site of liCNV disease, in the RPE/choroid, showed significant increases in proliferative, metabolic, and cytolytic activity. The addition of NK-cell-activating cytokine, rIL-18, further increased energetic capacity and biogenesis specifically in NK cells and occurred in parallel with a significant expansion of a cytolytic NK cell population. Together, these data suggest a unique mechanistic role for NK cells in responding to retinal damage through proliferation and cell-directed cytolytic mechanisms. Indeed, we found that NK cells can target ECs *in vitro* and that NK cells are found adjacent to endothelial capillaries in neovessels in human AMD donor retinal tissue. The appearance of perforin^{+} rings facing ECs in the liCNV lesion further supports a role for direct cell cytotoxicity in choroidal neovascularization. Therefore, we suggest an alternative method of NK cell restriction of pathogenesis through the cytolytic targeting of ECs at newly forming vessels.

While this is unexpected, it is not without precedent. NK cells play a critical regulatory role at the fetal-maternal interface during the first trimester of pregnancy, constituting 50%–90% of total lymphocytes in the uterus, and are responsible for remodeling the vasculature and establishing an immune modulatory environment for survival of the fetus.[17] Subsets of NK cells also play a regulatory role in the liver, where they maintain homeostasis and enable tissue regeneration after injury.[46] Additionally, NK cells are described to play a role in limiting tissue damage through immune editing, i.e., cytotoxic killing of activated immune cells.[47,48] Thus, NK cells act in a regulatory manner in a diverse range of contexts, and examining NK cell regulation of immune cells in nAMD will be of interest for further studies.

(E) Schematic representation of adoptive transfer of isolated, activated C57Bl/6J NK cells in a JR5558 model of spontaneous retinal vascularization (n = 3–4 mice/group).
(E) Fundus fluorescein angiography (FFA) was performed prior to (top), and 5 days following (bottom), NK cell administration.
(F) Quantification of leakage area from retinal neovascular lesions. Graphical data presented as mean ± SEM; statistical analysis by unpaired t test or two-way ANOVA with Fisher's LSD test where $^{*}p \leq 0.05$, $^{**}p < 0.01$.

To our knowledge, there are no reports on NK cell activity in the peripheral blood of individuals with AMD. Previously, it has been reported that older but otherwise healthy human donor NK cells were less cytotoxic than younger healthy donor NK cells, with a suggestion that this may contribute to incidence and progression of AMD.[11] However, our study addresses NK cell cytolytic activity and cytokine production in a human AMD disease cohort compared with healthy age-matched donors in fresh blood, and in fact contrary to the reported expectation, we find that the cytolytic capacity of NK cells in AMD is not only intact but also primed for enhanced activity.

The presence of circulating NK cells primed for cytolytic activity in AMD donors, and yet the progression of human disease raises the question of their real-world relevance in resolving disease. A major finding of significance in this study is that peripheral NK cells in the AMD cohort, despite being primed for cytolysis, present with a terminally differentiated phenotype, with increased γH2AX and CD57 expression when compared to age-matched healthy controls. The acquisition of CD57 expression on CD56^{dim} NK cells is associated with a gradual decline in proliferative capacity, and indeed we observed a striking decreased proliferative capacity in the AMD cohort compared to age-matched healthy controls. Given the importance of metabolically energetic, proliferating NK cells during active resolution of liCNV in our mouse model, this attenuated proliferative capacity of peripheral NK cells in nAMD likely underscores their reduced capacity to resolve neovascular lesions in nAMD when recruited to the site of disease.

Additionally, in parallel with the terminal phenotype observed in peripheral NK cells, we also found *CXCR4* and *CD96* were upregulated on NK cells in ocular tissue of nAMD compared to NK cells in a healthy donor eye. CXCR4 upregulation on NK cells has previously been associated with promotion of NK cell quiescence in liver cancer, while reprogrammed CXCR4^{+} decidual NK cells display a less activated, less cytotoxic phenotype but enhanced immunomodulatory potential during early pregnancy.[49] Evidence of specific upregulation of this marker of lower cytolytic capacity and enhanced quiescence was observed in the RPE/choroid of an nAMD donor compared to a non-disease donor, in parallel with increased expression of *CD96*, which has been correlated with human NK cell exhaustion and decreased expression of *NKG7*, an essential factor for cytotoxic degranulation.[50] These findings further suggest suppression of cytolytic and metabolic activity in NK cells at the site of disease in AMD. Taken together, these data imply that in isolated *ex vivo* conditions NK cells from AMD donors have enhanced cytolytic capability; however, upon recruitment to the lesion site, they may not proliferate to the extent needed to effect resolution. Furthermore, once in the disease micro-environment, the recruited NK cells may reprogram into a quiescent immune-tolerized phenotype, further inhibiting functionality.

We reasoned that introducing a bolus of activated NK cells may have therapeutic potential to resolve neovascularization. Both the liCNV model and the JR5558 model are acute models of neovascularization that ultimately resolve and are therefore amenable to illustrate whether NK cell therapy could be beneficial for accelerating or enhancing the resolution of the neovascular lesions. A final major advance of our study is the clear demonstration that NK cell immunotherapy in the form of adoptive transfer of either autologous or allogenic cytokine-activated NK cells significantly reduced neovascular lesion size and pathological leakage from the diseased vessels. These data strongly support the hypothesis that NK cells are resolving cells in the context of pathological neovascularization and that NK cell immunotherapy represents a transformative therapeutic strategy for the prevention and treatment of nAMD.

Overall, our study provides compelling evidence that NK cells are critical effectors in the systemic and local immune response to retinal injury and neovascular pathology in AMD and demonstrate their potential as therapeutic agents. By identifying a specific type 1/type 2 immune imbalance and uncovering a unique NK cell phenotype characterized by terminal differentiation and impaired proliferative potential in AMD, we redefine the role of innate lymphocytes in retinal immunopathology. Functionally, we show that retinal injury triggers systemic NK cell activation and that these cells exert direct cytotoxic effects on neovascular endothelium, a mechanism conserved across human disease and murine models. These findings not only expand our understanding of NK cell function in the eye but also offer new insights into vascular immunoregulation, with broader implications for the fields of retinal disease, vascular biology, and immunotherapy.

Limitations of the study

While this study identifies NK cells as an immunotherapeutic alternative for the treatment of neovascular AMD, it is not powered to identify sex as a modifier of response to treatment. Given recent evidence of the impact of sex on neovascular lesions,[51] it would be of interest to investigate the activation and resolving capacity of NK cells in males versus females in both liCNV mouse model and human disease.

Further characterization of the NK cells using recently reported subpopulation[52] would also improve our understanding of the nature of NK cell involvement in resolving liCNV. Expansion of functional human NK cell data to larger populations will allow for stratification of functional and phenotypic data to investigate if stage of AMD impacts NK cell activity.

RESOURCE AVAILABILITY

Lead contact

Requests for further information or resources should be directed to lead contact, Sarah L. Doyle (sarah.doyle@tcd.ie).

Materials availability

This study did not generate new unique reagents.

Data and code availability

- All data relevant to study are included in article or uploaded as supplemental information.
- scRNA-seq datasets have been deposited at the Gene Expression Omnibus and are publicly available as of the date of publication. Accession numbers are listed in the key resources table.
- This paper does not report original code. Any additional information required to reanalyze data reported in this work is available from lead contact upon request.

ACKNOWLEDGMENTS

We wish to thank all participants who donated blood for this project, the donors and their families for the healthy and AMD eye tissue, and TILDA participants for their valuable contribution to the study. We would like to thank the TILDA research team, study nurses, and administrators. The authors wish to acknowledge technical assistance from Dr. Barry Moran, Dr. Alfonso Blanco, and Ms Alison Murphy. Experimental schematics were created in BioRender by S.L.D. (2025/2026).

Supported by Science Foundation Ireland (SFI-15/CDA/3497 and SFI-18/TIDA/6067) to S.L.D.; Science Foundation Ireland (SFI/21SPP/3732) to S.L.D. and M.C.; Irish Research Council (IRCLA/2017/295) to S.L.D.; Health Research Board (HRB) Ireland with Fighting Blindness (HRB/MRCG-2018-08) to S.L.D.; European Research Council (ERC NK InSight – 101087802) to S.L.D.; Royal Victoria Eye and Ear Hospital (RVEEH) to S.L.D. and M.T.C.; F. Hoffmann-La Roche Immunology, Infectious Disease and Ophthalmology Incubator program, and BrightFocus Foundation to K.M.; and NIH (EY-024605) to R.F.M. TILDA was supported by Irish Government Department of Health, Atlantic Philanthropies, Irish Life, and HRB awards (TILDA-2017-1 and TILDA-2023-001). Funders had no involvement in study design, collection, analysis and interpretation of data, or authorship of the submitted work.

AUTHOR CONTRIBUTIONS

K.B., E.O., E.N., M.McE., L.G., S.P., K.P.B., K. Byrne, E.C., D.O'B., R.D., A.Z., E.F., and K.M. carried out experiments, processed and analyzed data. F.R., S.A., K.H., N.H., M.M.O'R., and K.R. processed and analyzed data. D.F., N.B., M.C., D.F., K.M., R.F.M., R.A.K., M.T.C., S.L.D. provided resources and analyzed data. All authors contributed to the final manuscript. S.L.D. conceived and directed the study.

DECLARATION OF INTERESTS

Trinity College Dublin has IP filed on data reported here.

STAR★METHODS

Detailed methods are provided in the online version of this paper and include the following:

- KEY RESOURCES TABLE
- EXPERIMENTAL MODEL AND STUDY PARTICIPANT DETAILS
  - Human cohorts
  - Primary cells
  - Cell lines
- METHOD DETAILS
  - Retinal photography and AMD grading – TILDA cohort
  - Retinal photograph and AMD grading – Phenotyping cohort
  - TILDA biomarker assessment
  - Human tissue preparation
  - Immunohistochemistry human tissue
  - Cell counting
  - Cell harvesting
  - Human lymphocyte characterisation & NK cell phenotyping - Flow cytometry
  - liCNV model
  - Immunohistochemistry mouse tissue
  - Digestion of RPE/choroid/sclera, retina & spleen
  - Single cell RNA-sequencing
  - Analysis of scRNA-seq data
  - OCR and ECAR measurement
  - Stimulation of cells
  - CD107a & IFN-γ assay (human)
  - Mouse lymphocyte characterisation and phenotyping
  - IFN-γ assay (mouse)
  - RNA extraction
  - cDNA preparation
  - Real time reverse-transcription polymerase chain reaction (RT-qPCR)
  - Caspase 3/7 incucyte assay
  - MTS/PMS assay
  - Lactate dehydrogenase (LDH) assay
  - Adoptive transfer models
  - Cryosectioning
  - RPE flatmount dissection and staining
  - Immunofluorescent staining of cryosections
  - Confocal microscopy
- QUANTIFICATION AND STATISTICAL ANALYSES

SUPPLEMENTAL INFORMATION

Supplemental information can be found online at https://doi.org/10.1016/j.xcrm.2026.102792.

Received: August 21, 2025
Revised: January 29, 2026
Accepted: April 10, 2026
Published: May 7, 2026

REFERENCES

1. Colijn, J.M., Buitendijk, G.H.S., Prokofyeva, E., Alves, D., Cachulo, M.L., Khawaja, A.P., Cougnard-Gregoire, A., Merle, B.M.J., Korb, C., Erke, M.G., et al. (2017). Prevalence of Age-Related Macular Degeneration in Europe: The Past and the Future. Ophthalmology *124*, 1753–1763. https://doi.org/10.1016/j.ophtha.2017.05.035.
2. Jager, R.D., Mieler, W.F., and Miller, J.W. (2008). Age-related macular degeneration. N. Engl. J. Med. *358*, 2606–2617. https://doi.org/10.1056/NEJMra0801537.
3. Campbell, M., and Doyle, S.L. (2019). Current perspectives on established and novel therapies for pathological neovascularization in retinal disease. Biochem. Pharmacol. *164*, 321–325. https://doi.org/10.1016/j.bcp.2019.04.029.
4. Daniel, E., Ying, G.S., Kim, B.J., Toth, C.A., Ferris, F., 3rd, Martin, D.F., Grunwald, J.E., Jaffe, G.J., Dunaief, J.L., Pan, W., et al. (2019). Five-Year Follow-up of Nonfibrotic Scars in the Comparison of Age-Related Macular Degeneration Treatments Trials. Ophthalmology *126*, 743–751. https://doi.org/10.1016/j.ophtha.2018.11.020.
5. Rofagha, S., Bhisitkul, R.B., Boyer, D.S., Sadda, S.R., and Zhang, K.; SEVEN-UP Study Group (2013). Seven-year outcomes in ranibizumab-treated patients in ANCHOR, MARINA, and HORIZON: a multicenter cohort study (SEVEN-UP). Ophthalmology *120*, 2292–2299. https://doi.org/10.1016/j.ophtha.2013.03.046.
6. Wallsh, J.O., and Gallemore, R.P. (2021). Anti-VEGF-Resistant Retinal Diseases: A Review of the Latest Treatment Options. Cells *10*, 1049. https://doi.org/10.3390/cells10051049.
7. Yang, S., Zhao, J., and Sun, X. (2016). Resistance to anti-VEGF therapy in neovascular age-related macular degeneration: a comprehensive review. Drug Des. Devel. Ther. *10*, 1857–1867. https://doi.org/10.2147/DDDT.S97653.
8. Guillonneau, X., Eandi, C.M., Paques, M., Sahel, J.A., Sapieha, P., and Sennlaub, F. (2017). On phagocytes and macular degeneration. Prog. Retin. Eye Res. *61*, 98–128. https://doi.org/10.1016/j.preteyeres.2017.06.002.
9. Yu, C., Roubeix, C., Sennlaub, F., and Saban, D.R. (2020). Microglia versus Monocytes: Distinct Roles in Degenerative Diseases of the Retina. Trends Neurosci. *43*, 433–449. https://doi.org/10.1016/j.tins.2020.03.012.
10. Wong, J.H.C., Ma, J.Y.W., Jobling, A.I., Brandli, A., Greferath, U., Fletcher, E.L., and Vessey, K.A. (2022). Exploring the pathogenesis of age-related macular degeneration: A review of the interplay between retinal pigment epithelium dysfunction and the innate immune system. Front. Neurosci. *16*, 1009599. https://doi.org/10.3389/fnins.2022.1009599.

11. Dong, X., Song, Y., Liu, Y., Kou, X., Yang, T., Shi, S.X., He, K., Li, Y., Li, Z., Yao, X., et al. (2024). Natural killer cells promote neutrophil extracellular traps and restrain macular degeneration in mice. Sci. Transl. Med. *16*, eadi6626. https://doi.org/10.1126/scitranslmed.adi6626.

12. Voigt, A.P., Mulfaul, K., Mullin, N.K., Flamme-Wiese, M.J., Giacalone, J.C., Stone, E.M., Tucker, B.A., Scheetz, T.E., and Mullins, R.F. (2019). Single-cell transcriptomics of the human retinal pigment epithelium and choroid in health and macular degeneration. Proc. Natl. Acad. Sci. USA *116*, 24100–24107. https://doi.org/10.1073/pnas.1914143116.

13. Voigt, A.P., Binkley, E., Flamme-Wiese, M.J., Zeng, S., DeLuca, A.P., Scheetz, T.E., Tucker, B.A., Mullins, R.F., and Stone, E.M. (2020). Single-Cell RNA Sequencing in Human Retinal Degeneration Reveals Distinct Glial Cell Populations. Cells *9*, 438. https://doi.org/10.3390/cells9020438.

14. Voigt, A.P., Whitmore, S.S., Lessing, N.D., DeLuca, A.P., Tucker, B.A., Stone, E.M., Mullins, R.F., and Scheetz, T.E. (2020). Spectacle: An interactive resource for ocular single-cell RNA sequencing data analysis. Exp. Eye Res. *200*, 108204. https://doi.org/10.1016/j.exer.2020.108204.

15. Voigt, A.P., Whitmore, S.S., Mulfaul, K., Chirco, K.R., Giacalone, J.C., Flamme-Wiese, M.J., Stockman, A., Stone, E.M., Tucker, B.A., Scheetz, T.E., and Mullins, R.F. (2020). Bulk and single-cell gene expression analyses reveal aging human choriocapillaris has pro-inflammatory phenotype. Microvasc. Res. *131*, 104031. https://doi.org/10.1016/j.mvr.2020.104031.

16. Lodoen, M.B., and Lanier, L.L. (2006). Natural killer cells as an initial defense against pathogens. Curr. Opin. Immunol. *18*, 391–398. https://doi.org/10.1016/j.coi.2006.05.002.

17. Vento-Tormo, R., Efremova, M., Botting, R.A., Turco, M.Y., Vento-Tormo, M., Meyer, K.B., Park, J.E., Stephenson, E., Polański, K., Goncalves, A., et al. (2018). Single-cell reconstruction of the early maternal-fetal interface in humans. Nature *563*, 347–353. https://doi.org/10.1038/s41586-018-0698-6.

18. Clausen, J., Vergeiner, B., Enk, M., Petzer, A.L., Gastl, G., and Gunsilius, E. (2003). Functional significance of the activation-associated receptors CD25 and CD69 on human NK-cells and NK-like T-cells. Immunobiology *207*, 85–93. https://doi.org/10.1078/0171-2985-00219.

19. Eckelhart, E., Warsch, W., Zebedin, E., Simma, O., Stoiber, D., Kolbe, T., Rülicke, T., Mueller, M., Casanova, E., and Sexl, V. (2011). A novel Ncr1-Cre mouse reveals the essential role of STAT5 for NK-cell survival and development. Blood *117*, 1565–1573. https://doi.org/10.1182/blood-2010-06-291633.

20. Doyle, S.L., Adamson, P., López, F.J., Humphries, P., and Campbell, M. (2014). Reply to IL-18 is not therapeutic for neovascular age-related macular degeneration. Nat. Med. *20*, 1376–1377. https://doi.org/10.1038/nm.3741.

21. Doyle, S.L., López, F.J., Celkova, L., Brennan, K., Mulfaul, K., Ozaki, E., Kenna, P.F., Kurali, E., Hudson, N., Doggett, T., et al. (2015). IL-18 Immunotherapy for Neovascular AMD: Tolerability and Efficacy in Nonhuman Primates. Investig. Ophthalmol. Vis. Sci. *56*, 5424–5430. https://doi.org/10.1167/iovs.15-17264.

22. Doyle, S.L., Ozaki, E., Brennan, K., Humphries, M.M., Mulfaul, K., Keaney, J., Kenna, P.F., Maminishkis, A., Kiang, A.S., Saunders, S.P., et al. (2014). IL-18 attenuates experimental choroidal neovascularization as a potential therapy for wet age-related macular degeneration. Sci. Transl. Med. *6*, 230ra44. https://doi.org/10.1126/scitranslmed.3007616.

23. Yang, R., Wang, M., Zhang, G., Li, Y., Wang, L., and Cui, H. (2021). POU2F2 regulates glycolytic reprogramming and glioblastoma progression via PDPK1-dependent activation of PI3K/AKT/mTOR pathway. Cell Death Dis. *12*, 433. https://doi.org/10.1038/s41419-021-03719-3.

24. Borrego, F., Robertson, M.J., Ritz, J., Peña, J., and Solana, R. (1999). CD69 is a stimulatory receptor for natural killer cell and its cytotoxic effect is blocked by CD94 inhibitory receptor. Immunology *97*, 159–165. https://doi.org/10.1046/j.1365-2567.1999.00738.x.

25. Cibrián, D., and Sánchez-Madrid, F. (2017). CD69: from activation marker to metabolic gatekeeper. Eur. J. Immunol. *47*, 946–953. https://doi.org/10.1002/eji.201646837.

26. Marquardt, N., Kekäläinen, E., Chen, P., Lourda, M., Wilson, J.N., Scharenberg, M., Bergman, P., Al-Ameri, M., Hård, J., Mold, J.E., et al. (2019). Unique transcriptional and protein-expression signature in human lung tissue-resident NK cells. Nat. Commun. *10*, 3841. https://doi.org/10.1038/s41467-019-11632-9.

27. Borchers, M.T., Harris, N.L., Wesselkamper, S.C., Vitucci, M., and Cosman, D. (2006). NKG2D ligands are expressed on stressed human airway epithelial cells. Am. J. Physiol. Lung Cell. Mol. Physiol. *291*, L222–L231. https://doi.org/10.1152/ajplung.00327.2005.

28. Gasser, S., Orsulic, S., Brown, E.J., and Raulet, D.H. (2005). The DNA damage pathway regulates innate immune system ligands of the NKG2D receptor. Nature *436*, 1186–1190. https://doi.org/10.1038/nature03884.

29. Lin, D., Lavender, H., Soilleux, E.J., and O'Callaghan, C.A. (2012). NF-kappaB regulates MICA gene transcription in endothelial cell through a genetically inhibitable control site. J. Biol. Chem. *287*, 4299–4310. https://doi.org/10.1074/jbc.M111.282152.

30. Makita, K., Otsuka, N., Tomaru, U., Taniguchi, K., and Kasahara, M. (2023). NKG2D Ligand Expression Induced by Oxidative Stress Mitigates Cutaneous Ischemia-Reperfusion Injury. J. Histochem. Cytochem. *71*, 61–72. https://doi.org/10.1369/00221554221147582.

31. McCarthy, M.T., Moncayo, G., Hiron, T.K., Jakobsen, N.A., Valli, A., Soga, T., Adam, J., and O'Callaghan, C.A. (2018). Purine nucleotide metabolism regulates expression of the human immune ligand MICA. J. Biol. Chem. *293*, 3913–3924. https://doi.org/10.1074/jbc.M117.809459.

32. Tran, T., Galdina, V., Urquidi, O., Reis Galvão, D., Rieben, R., Adachi, T.B.M., Puga Yung, G.L., and Seebach, J.D. (2024). Assessment of NK cytotoxicity and interactions with porcine endothelial cells by live-cell imaging in 2D static and 3D microfluidic systems. Sci. Rep. *14*, 24199. https://doi.org/10.1038/s41598-024-75217-3.

33. Oka, N., Markova, T., Tsuzuki, K., Li, W., El-Darawish, Y., Pencheva-Demireva, M., Yamanishi, K., Yamanishi, H., Sakagami, M., Tanaka, Y., and Okamura, H. (2020). IL-12 regulates the expansion, phenotype, and function of murine NK cells activated by IL-15 and IL-18. Cancer Immunol. Immunother. *69*, 1699–1712. https://doi.org/10.1007/s00262-020-02553-4.

34. Chaix, J., Tessmer, M.S., Hoebe, K., Fuséri, N., Ryffel, B., Dalod, M., Alexopoulou, L., Beutler, B., Brossay, L., Vivier, E., and Walzer, T. (2008). Cutting edge: Priming of NK cells by IL-18. J. Immunol. *181*, 1627–1631. https://doi.org/10.4049/jimmunol.181.3.1627.

35. Fallone, L., Pouxvielh, K., Arbez, L., Rousseaux, N., Picq, L., Drouillard, A., Mathieu, A.L., Nombel, A., Benezech, S., Bourdonnay, E., et al. (2025). Interleukins 15 and 18 synergistically prime the antitumor function of natural killer cells through noncanonical activation of mTORC1. Sci. Signal. *18*, eadq8778. https://doi.org/10.1126/scisignal.adq8778.

36. Wiedemann, G.M., Grassmann, S., Lau, C.M., Rapp, M., Villarino, A.V., Friedrich, C., Gasteiger, G., O'Shea, J.J., and Sun, J.C. (2020). Divergent Role for STAT5 in the Adaptive Responses of Natural Killer Cells. Cell Rep. *33*, 108498. https://doi.org/10.1016/j.celrep.2020.108498.

37. Correia, A.L., Guimaraes, J.C., Auf der Maur, P., De Silva, D., Trefny, M.P., Okamoto, R., Bruno, S., Schmidt, A., Mertz, K., Volkmann, K., et al. (2021). Hepatic stellate cells suppress NK cell-sustained breast cancer dormancy. Nature *594*, 566–571. https://doi.org/10.1038/s41586-021-03614-z.

38. Nielsen, C.M., White, M.J., Goodier, M.R., and Riley, E.M. (2013). Functional Significance of CD57 Expression on Human NK Cells and Relevance to Disease. Front. Immunol. *4*, 422. https://doi.org/10.3389/fimmu.2013.00422.

39. Kared, H., Martelli, S., Ng, T.P., Pender, S.L.F., and Larbi, A. (2016). CD57 in human natural killer cells and T-lymphocytes. Cancer Immunol. Immunother. *65*, 441–452. https://doi.org/10.1007/s00262-016-1803-z.

40. Wang, P., Li, X., Wang, J., Gao, D., Li, Y., Li, H., Chu, Y., Zhang, Z., Liu, H., Jiang, G., et al. (2017). Re-designing Interleukin-12 to enhance its safety and potential as an anti-tumor immunotherapeutic agent. Nat. Commun. *8*, 1395. https://doi.org/10.1038/s41467-017-01385-8.

41. Wong, W.L., Su, X., Li, X., Cheung, C.M.G., Klein, R., Cheng, C.Y., and Wong, T.Y. (2014). Global prevalence of age-related macular degeneration and disease burden projection for 2020 and 2040: a systematic review and meta-analysis. Lancet. Glob. Health *2*, e106–e116. https://doi.org/10.1016/S2214-109X(13)70145-1.

42. Hazeldine, J., Hampson, P., and Lord, J.M. (2012). Reduced release and binding of perforin at the immunological synapse underlies the age-related decline in natural killer cell cytotoxicity. Aging Cell *11*, 751–759. https://doi.org/10.1111/j.1474-9726.2012.00839.x.

43. Le Garff-Tavernier, M., Béziat, V., Decocq, J., Siguret, V., Gandjbakhch, F., Pautas, E., Debré, P., Merle-Beral, H., and Vieillard, V. (2010). Human NK cells display major phenotypic and functional changes over the life span. Aging Cell *9*, 527–535. https://doi.org/10.1111/j.1474-9726.2010.00584.x.

44. van Weel, V., Toes, R.E.M., Seghers, L., Deckers, M.M.L., de Vries, M.R., Eilers, P.H., Sipkens, J., Schepers, A., Eefting, D., van Hinsbergh, V.W.M., et al. (2007). Natural killer cells and CD4+ T-cells modulate collateral artery development. Arterioscler. Thromb. Vasc. Biol. *27*, 2310–2318. https://doi.org/10.1161/ATVBAHA.107.151407.

45. Deliyanti, D., Suphapimol, V., Ang, P., Tang, X., Jayasimhan, A., and Wilkinson-Berka, J.L. (2023). Early Depletion of Neutrophils Reduces Retinal Inflammation and Neovascularization in Mice with Oxygen-Induced Retinopathy. Int. J. Mol. Sci. *24*, 15680. https://doi.org/10.3390/ijms242115680.

46. Tosello-Trampont, A., Surette, F.A., Ewald, S.E., and Hahn, Y.S. (2017). Immunoregulatory Role of NK Cells in Tissue Inflammation and Regeneration. Front. Immunol. *8*, 301. https://doi.org/10.3389/fimmu.2017.00301.

47. Morandi, B., Mortara, L., Chiossone, L., Accolla, R.S., Mingari, M.C., Moretta, L., Moretta, A., and Ferlazzo, G. (2012). Dendritic cell editing by activated natural killer cells results in a more protective cancer-specific immune response. PLoS One *7*, e39170. https://doi.org/10.1371/journal.pone.0039170.

48. Verbsky, J.W., and Grossman, W.J. (2006). Hemophagocytic lymphohistiocytosis: diagnosis, pathophysiology, treatment, and future perspectives. Ann. Med. *38*, 20–31. https://doi.org/10.1080/07853890500465189.

49. Tao, Y., Li, Y.H., Zhang, D., Xu, L., Chen, J.J., Sang, Y.F., Piao, H.L., Jing, X.L., Yu, M., Fu, Q., et al. (2021). Decidual CXCR4(+) CD56(bright) NK cells as a novel NK subset in maternal-foetal immune tolerance to alleviate early pregnancy failure. Clin. Transl. Med. *11*, e540. https://doi.org/10.1002/ctm2.540.

50. Ng, S.S., De Labastida Rivera, F., Yan, J., Corvino, D., Das, I., Zhang, P., Kuns, R., Chauhan, S.B., Hou, J., Li, X.Y., et al. (2020). The NK cell granule protein NKG7 regulates cytotoxic granule exocytosis and inflammation. Nat. Immunol. *21*, 1205–1218. https://doi.org/10.1038/s41590-020-0758-6.

51. Yan, C., Figueiredo, C.A., Pompös, I.M., Ugursu, B., Arribas-Lange, P., Skosyrski, S., Yang, S., Althoff, P., Kociok, N., Joussen, A.M., and Wolf, S.A. (2025). Sex differences on laser-induced choroidal neovascularization and short-chain fatty acid treatment in a mouse model. J. Neuroinflammation *22*, 188. https://doi.org/10.1186/s12974-025-03508-1.

52. Rebuffet, L., Melsen, J.E., Escalière, B., Basurto-Lozada, D., Bhandoola, A., Björkström, N.K., Bryceson, Y.T., Castriconi, R., Cichocki, F., Colonna, M., et al. (2024). High-dimensional single-cell analysis of human natural killer cell heterogeneity. Nat. Immunol. *25*, 1474–1488. https://doi.org/10.1038/s41590-024-01883-0.

53. Connolly, E., Rhatigan, M., O'Halloran, A.M., Muldrew, K.A., Chakravarthy, U., Cahill, M., Kenny, R.A., and Doyle, S.L. (2018). Prevalence of age-related macular degeneration associated genetic risk factors and 4-year progression data in the Irish population. Br. J. Ophthalmol. *102*, 1691–1695. https://doi.org/10.1136/bjophthalmol-2017-311673.

54. Bird, A.C., Bressler, N.M., Bressler, S.B., Chisholm, I.H., Coscas, G., Davis, M.D., de Jong, P.T., Klaver, C.C., Klein, B.E., Klein, R., et al. (1995). An international classification and grading system for age-related maculopathy and age-related macular degeneration. The International ARM Epidemiological Study Group. Surv. Ophthalmol. *39*, 367–374. https://doi.org/10.1016/s0039-6257(05)80092-x.

55. Wolf, F.A., Angerer, P., and Theis, F.J. (2018). SCANPY: large-scale single-cell gene expression data analysis. Genome Biol. *19*, 15. https://doi.org/10.1186/s13059-017-1382-0.

56. Wolock, S.L., Lopez, R., and Klein, A.M. (2019). Scrublet: Computational Identification of Cell Doublets in Single-Cell Transcriptomic Data. Cell Syst. *8*, 281–291.e9. https://doi.org/10.1016/j.cels.2018.11.005.

57. Tirosh, I., Izar, B., Prakadan, S.M., Wadsworth, M.H., 2nd, Treacy, D., Trombetta, J.J., Rotem, A., Rodman, C., Lian, C., Murphy, G., et al. (2016). Dissecting the multicellular ecosystem of metastatic melanoma by single-cell RNA-seq. Science *352*, 189–196. https://doi.org/10.1126/science.aad0501.

58. Hie, B., Bryson, B., and Berger, B. (2019). Efficient integration of heterogeneous single-cell transcriptomes using Scanorama. Nat. Biotechnol. *37*, 685–691. https://doi.org/10.1038/s41587-019-0113-3.

STAR★METHODS

KEY RESOURCES TABLE

REAGENT or RESOURCE	SOURCE	IDENTIFIER
Antibodies		
Goat Anti-Rat IgG H&L (Alexa Fluor® 594)	Abcam	Cat#ab150160; RRID: AB_2756445
Goat Anti-Rabbit IgG H&L (Alexa Fluor® 488)	Abcam	Cat# ab150077; RRID: AB_2630356
Goat Anti-Rabbit IgG H&L (Alexa Fluor® 647)	Abcam	Cat# ab150079; RRID:AB_2722623
Isolectin GS-IB4 From Griffonia simplicifolia, Alexa Fluor^{TM} 568 Conjugate	Invitrogen	Cat# I21413; RRID:AB_2314661
Hoechst stain (bisBenzimide H 33342 trihydrochloride)	Merck	Cat# B2261; RRID:AB_10626776
Anti-human/mouse Perforin	Proteintech	Cat#: 14580-1-AP; RRID:AB_10639524
Anti-human CD56 BV650, clone HCD56	BioLegend	Cat# 318344 LOT; B381674; RRID:AB_2563838
Anti-human CD3 PECy5.5, clone SK7	Invitrogen	Cat# 35-0036-42 LOT; 2689404; RRID:AB_11220085
Anti-human CD4-APCCy7, clone RPA-T4,	BioLegend	Cat#300518; LOT; B384817; RRID:AB_314086
Anti-human CD8-AF647, clone SK1	Biolegend	Cat#344726; LOT; B367004; RRID:AB_2563452
Anti-human CD56-PECy7, clone HCD56	BioLegend	Cat#318318; LOT; B438796; RRID:AB_604107
Anti-human CD3-FITC, clone UCHT1	BioLegend	Cat#300406; LOT;B390953; RRID:AB_314060
Anti-human CD107a-APCCy7 clone H4A3	BioLegend	Cat#328620; LOT;B377421; RRID:AB_1279055
Anti-human IFNy-PE, clone B27	BioLegend	Cat#506507 LOT;B426566; RRID:AB_315440
Anti-human CD57-PECy7, clone HNK-1	BioLegend	Cat#359624; LOT;B359173; RRID:AB_2632689
Anti-human yH2A.X Phospho (Ser139)-PE, clone 2F3	Biolegend	Cat#613412; LOT B40112; RRID:AB_2616871
Anti-human Ki67-AF488, BD, clone B56	BD	Cat#561165; LOT 2130145; RRID:AB_10611866
Anti- mouse CD45-BB515, clone 30-F11	BD	Cat# 564590; LOT 4185387/4057991; RRID:AB_2738857
Anti-mouse NK1.1-BB700, clone PK136	BD	Cat#556503; LOT 0310129; RRID:AB_2744491
Anti-mouse CD3-VioBlue, clone 17A2	Miltenyi	Cat# 130-118-849; LOT 5240808624, RRID:AB_2751568
Anti-mouse CD4-AF700, clone GK1.5	Biolegend	Cat# 100429, LOT B355896, RRID:AB_493698
Anti-mouse CD8-APCCy7, clone 53–6.7	Biolegend	Cat# 100713; LOT B332483, RRID:AB_312752
Anti-mouse Ki67-PECy7, clone SolA15	Invitrogen	Cat# 25-5698-80; LOT 2504588, RRID:AB_11217689
Anti-mouse CD69-BV650, clone H1.2F3	Biolegend,	Cat# 104541; LOT B317453, RRID:AB_2616934
Anti-mouse CD25-PE, clone PC61.5	Invitrogen	Cat# 12-0251-82; LOT 2506167, RRID:AB_465607
Anti-mouse IFNy-PECy7, clone XMG1.2	Biolegend	Cat# 505825, RRID:AB_1595591
Anti-mouse CD3-FITC, clone 17A2	Biolegend,	Cat# 100204, RRID:AB_312661
Anti-mouse CD69-PECy5, clone H1.2F3	Biolegend	Cat# 104509, RRID:AB_313112
Anti-mouse NK1.1-e450, clone PK136	Invitrogen	Cat# 48-5941-82, RRID:AB_2043877
Biological samples		
Human Primary Blood Mononuclear Cells	Isolated in-house	NA
Human Donor Eyes	Iowa Lions Eye Bank	NA
Mouse Spleen-derived NK cells	Isolated in-house	NA
Chemicals, peptides, and recombinant proteins		
bisBenzimide H 33342 trihydrochloride (Hoechst)	Sigma Aldrich	Cat#B2261
Pierce^{TM} 16% Formaldehyde (w/v), Methanol-free	Fisher Scientific	Cat# 28908
PBS	Merck	Cat# D8662-500ML
Goat serum	Sigma Aldrich	Cat# G9023
Triton^{TM} X-100	Merck	Cat# X100
CellTiter 96® Aqueous MTS Reagent Powder	Promega	Cat# G1112
Trypsin-EDTA 0.25%	Gibco	Cat# 25200056

(Continued on next page)

CellPress
OPEN ACCESS

Continued

REAGENT or RESOURCE	SOURCE	IDENTIFIER
Fetal Bovine Serum (FBS)	Merck	Cat# F9665-500ML
Pen-Strep	Merck	Cat# P4333-100ML
OCT mounting media	VWR	Cat# 361603E
MMLV reverse transcriptase	Promega	Cat# PAM5313
10nm Random Hexamers	IDT	Cat# 51-01-18-25
RNaseOUT™ Recombinant Ribonuclease Inhibitor	Invitrogen	Cat# 10777019
RPMI	Merck	Cat# R8758-500ML
Myo-Inositol	Sigma	Cat# I7508-50G
L-Glutamine	Sigma	Cat# G8540-10MG
Folic Acid	Sigma	Cat# F7876-1G
Horse Serum	Sigma	Cat# H1270-500ML
LIVE/DEAD™ Fixable Aqua Dead Cell Stain Kit, for 405 nm excitation	Thermo Fisher	Cat# L34957
Proteinase K	Thermo Fisher	Cat# EO0491
Ethidium Bromide solution	Thermo Fisher	Cat# 17898
HBSS	BioSciences	Cat# 14025050
HEPES Buffer	Merck	Cat# C-40010
DNAse I	Merck	Cat# 10104159001
Bovine Serum Albumin	Merck	Cat# 05470
Protease Inhibitor Cocktail	Merck	Cat# P8340
Triton X-100	Merck	Cat# 93422
Endothelial Cell Growth Medium MV2 Kit	Merck	Cat# C-22121
Lymphoprep	Stemcell Technologies	Cat# 18061
Hyclone Trypan Blue	Thermo Fischer	Cat# SV3008401
Murine Recombinant IL-18	GSK/Biotechne	Cat# 9139-IL *
Human Recombinant IL-18	Biotechne	Cat# 9124-IL
Human IL-12	Miltenyi Biotec	Cat# 130-096-705
Recombinant Mouse IL-12	Biotechne	Cat# 419-ML
Human IL-15	Miltenyi Biotec	Cat# 130-095-760
Murine Recombinant IL-15	Peprotech®	Cat# 210-15-10UG
BD Cytofix/CytoPerm	BD Biosciences	Cat# 554722; RRID: AB_2869010
BD GolgiPlug	BD Biosciences	Cat# 15847968
Incucyte® Caspase-3/7 Dye for Apoptosis	Sartorius	Cat# 4440
Etoposide	Thermo Fisher	Cat# J63651.MB
Oligomycin	Sigma	Cat# O4876
Rotenone	Sigma	Cat# 557368
Antimycin	Sigma	Cat# A8674
2-deoxyglucose	Sigma	Cat# D8375
**Critical commercial assays**		
E.Z.N.A.® Total RNA Kit I	Omega-BIO-TEK	Cat# R6834-02
SensiFAST™ SYBR® No-ROX Kit	Bioline/Meridian Bioscience	Cat# BIO-98020
Mouse NK Cell Isolation Kit	Miltenyi	Cat# 130-115-818
CyQuant LDH Cytotoxicity Assay	Life Technologies	Cat# C20301
**Deposited data**		
Mouse liCNV scRNA-seq	This paper	GEO: GSE318755
Mouse IL-18 scRNA-seq datset	This paper	GEO: GSE317763

(*Continued on next page*)

Continued		
REAGENT or RESOURCE	SOURCE	IDENTIFIER
Single-cell transcriptomics of the human retinal pigment epithelium and choroid in health and macular degeneration (human)	Voigt AP et al., 2019	GEO: GSE135922
Experimental models: Cell lines		
Human: Human Retinal Microvascular Endothelial Cells	Cell Systems	Cat# ACBRI 181
Human: NK-92MI	ATCC	Cat# CRL-2408; RRID:CVCL_3755
Human: LCL 721.221	Merck	RRID: CVCL_6263
Experimental models: Organisms/strains		
C57Bl/6J	The Jackson Laboratory	RRID:IMSR_JAX:000664
NCR1-GFP Mice	Eckelhart et al., 2011	
JR5558 Mice	The Jackson Laboratory	RRID:IMSR_JAX:005558
Oligonucleotides		
For Primer Sequences for qPCR Analysis, See Methods & Figure S7A		
Software and algorithms		
ImageJ/Fiji	NIH	https://imagej.nih.gov/ij
GraphPad Prism	GraphPad	https://www.graphpad.com
LasX microscope analysis software	Leica	https://www.leica-microsystems.com/products/microscope-software/p/leica-las-x-ls/downloads/
Incucyte analysis 2024B	Sartorius	https://downloads.essenbioscience.com/get/incucyte-2022b-rev2-gui
FlowJo^{TM} v10.8–10 Software	BD	https://www.flowjo.com/flowjo10/download
Cell Ranger Single-Cell Software Suite v5.0	10x Genomics	https://www.10xgenomics.com/support/software/cell-ranger/downloads
Scanpy – Single-Cell Analysis in Python	Github	https://github.com/scverse/scanpy/tree/main
Scrublet	Github	https://github.com/swolock/scrublet
Scanorama	Github	https://github.com/brianhie/scanorama
R Statistical Software v4.3.1	R Core Team	https://www.r-project.org/

EXPERIMENTAL MODEL AND STUDY PARTICIPANT DETAILS

Human cohorts

The Irish Longitudinal Study on Aging (TILDA) cohort

The Irish Longitudinal Study on Aging (TILDA) is a nationally representative, longitudinal cohort study of >8,500 community-dwelling adults aged 50 and older living in the Republic of Ireland. Initiated in 2009, TILDA follows participants longitudinally to assess changes in health, economic, and social circumstances, providing comprehensive data to inform policy and research on aging. The study encompasses multiple waves of data collection through computer assisted personal interviews (CAPI), conducted by trained interviewers in the participants own home every two years, and a health assessment collecting biomedical data including anthropological measures, blood samples and retinal photography, occurring every second wave. Participants selected for this study have been described elsewhere.[53] In brief, participants were selected from the TILDA cohort who had provided a blood sample and had retinal images taken to detect the presence of AMD. 4,473 participants were selected in total, of this 4,173 (60 ± 9 years) had no AMD and 300 (64 ± 9 years) people had any AMD. A subset of 194 participants had data on AMD severity, an overview of this sample is given in Figure S1A. Ethical approval was granted by the Faculty of Health Sciences Research Ethics Committee at Trinity College Dublin, and all participants provided written informed consent. All experimental procedures were in accordance with the tenets of the Declaration of Helsinki.

Lymphocyte characterisation cohort

For characterization of healthy versus control peripheral blood, 30 participants diagnosed with AMD (75.87 ± 10.77 years), and 33 non-AMD controls (70.97 ± 8.64 years) were recruited from Progressive Vision Research in Dublin (Figure S2A). Ethical approval was granted by the Faculty of Health Sciences Research Ethics Committee at Trinity College Dublin, and all participants provided written informed consent. All experimental procedures were in accordance with the tenets of the Declaration of Helsinki.

CellPress
OPEN ACCESS

NK cell function cohort

For NK cell functional assays, 16 nAMD donors (80.06 ± 8.18 years) and 10 non-AMD donors (73.7 ± 6.27 years) were recruited. Ethical approval was granted by the Faculty of Health Sciences Research Ethics Committee at Trinity College Dublin, and all participants provided written informed consent. All experimental procedures were in accordance with the tenets of the Declaration of Helsinki.

Human donor eye tissue

Human donor eyes were obtained by the Iowa Lions Eye Bank (Iowa City, IA, USA). All experiments were conducted in accordance with the Declaration of Helsinki and with full consent of the donors' next of kin for the evaluation of medical records and the use of these tissues for biomedical research.

Primary cells

Human peripheral blood mononuclear cells

Peripheral blood mononuclear cells (PBMCs) were isolated from venous blood of volunteers from whom written and informed consent had been obtained and ethics approval granted by Trinity College Dublin Research Ethics Committee. Bloods were collected into EDTA-coated vacutubes and 15 mL of lymphoprep (BioSciences) was added to a 50 mL SepMate tube (Stem Cell Technologies) by pipetting through the central hole of the SepMate insert. Blood samples were then diluted with an equal volume of PBS. These diluted blood samples were then added to the SepMate tube by pipetting down the side of the tube. These tubes were then centrifuged at 1200 x *g* for 10 min with no brake applied, or if the samples were older than 24 h the spin was set to 20 min long. The top layer containing the PBMCs was poured off into a fresh tube and these were washed with PBS twice by spinning at 300 x *g* for 8 min at room temperature. The cell pellet was resuspended in red-cell lysis buffer and centrifuged again. Finally, cells were resuspended in RPMI 1640 (Sigma) supplemented with 10% fetal bovine serum (FBS) and 1% Penicillin/Streptomycin (P/S), counted, and plated in U-bottom 96 well plates. They were maintained at 37°C and 5% CO_2.

Human retinal microvascular endothelial cells

Primary Human Retinal Microvascular Endothelial cells (HRMECs) were purchased from Cell Systems (ACBRI 181, lot #181.03.03.02.02) and were from an adult male donor. HRMECs were cultured in basal Endothelial Media MV2 (Promocell) supplemented with 5% FBS, 1% P/S and growth factors including; recombinant human epidermal growth factor (5 ng/mL), recombinant human basic fibroblast growth factor (10 ng/mL), Insulin-like growth factor (20 ng/mL), recombinant human Vascular Endothelial Growth Factor (0.5 ng/mL), ascorbic acid (1 mg/mL) and hydrocortisone (0.2 mg/mL).

To induce senescence, HRMECs were grown in T175 flasks before being treated with 20 μM of etoposide made in basal endothelial media MV2 for 24 h. After 24 h, the etoposide-containing media was replaced with normal media and cells were allowed to recover for 4 days before use.

Cell lines

NK-92MI

This cell line was derived from peripheral blood mononuclear cells from a 50-year-old, White male with rapidly progressive non-Hodgkin's lymphoma. The parental cells were stably transfected with human IL-2 cDNA in the retroviral MFG-hIL-2 vector by particle-mediated gene transfer. NK-92MI cells were maintained in RPMI-1640 medium (Sigma Aldrich) supplemented with 1% P/S, 12.5% FBS, 12.5% horse serum, 0.02 mM Folic Acid (Sigma-Aldrich), 0.2 mM Myoinositol (Sigma-Aldrich), 2 mM Glutamine (Sigma-Aldrich) and 0.1 mM β-mercaptoethanol.

721.221

721.221 cells were a kind gift from Prof. Clair Gardiner (TCD) and were maintained in RPMI 1640 (Sigma-Aldrich) supplemented with 10% FBS and 1% P/S. The 721.221 cell line was derived from a human adult female 721 lymphoblastoid cell line exposed to gamma radiation, inducing null mutations in endogenous HLA-A, B, and C class I antigen genes.

Cell lines were not formally authenticated or tested for mycoplasma.

Mice

All mice used for this project were housed in a specific pathogen-free (SPF) environment that was monitored daily by animal welfare staff. All mouse lines were maintained on a C57Bl/6J background. Mice were allowed free access to food and water and were maintained on a 12-h light/dark cycle, with lights turned on at 8 a.m. and turned off at 8 p.m. All animal experiments were assessed and approved by an internal ethics committee at TCD and were all under project licenses approved by the Health Products Regulatory Authority (HPRA). All studies adhered to the Association for Research in Vision and Ophthalmology (ARVO) statement for the use of animals in ophthalmic and vision research. Littermates of the same sex were randomly assigned to experimental groups.

NCR1-GFP

The NCR1-GFP mice were provided as a gift from the Sexl laboratory.[19] Briefly, each mouse possesses an endogenous Green Fluorescent Protein (Gfp) transcript which is flanked by adjacent *LoxP* sites. NK specific Cre recombinase expression can then excise these *LoxP* sites allowing for the NK-specific expression of GFP. To ensure single copy number of Cre recombinase in each animal, the strain was maintained through the crossbreeding of Cre^+ and Cre^- animals all on the background of GFP reporter mice, (B6.Cg-Gt(ROSA)26Sortm1(rtTA, EGFP)Nagy/J). Both male and female mice aged 6–16 weeks were used for experiments. Mice were housed in a specific pathogen-free environment throughout the course of treatment. Numbers of animals used per experiment are provided in the figure legends.

JR5558

JR5558 mice that harbored the Rd8 mutation were purchased from Jackon laboratories for experimentation, RRID:IMSR_-JAX:005558. Mice were housed in a specific pathogen-free environment throughout the course of treatment and were used at 6 weeks of age.

METHOD DETAILS

Retinal photography and AMD grading – TILDA cohort

Retinal photography was collected from TILDA participants by trained research nurses during the first wave of health assessments. Images were acquired using a NIDEK AFC-210 non-mydriatic auto-fundus camera (NIDEK, Aichi, Japan) through a non-dilated pupil. Images were centered on the macula and included the optic disc (Early Treatment Diabetic Retinopathy Study [ETDRS] standard field 2). All participants were assessed for the presence of Early or Late AMD, with a subsample of longitudinal participants having AMD severity graded using a modified version of the International Classification and Grading System for AMD with the age-related maculopathy (ARM) category replaced by three categories of early AMD: Early Mild (>10 hard macular drusen <63 mm), early moderate (at least one soft drusen >125 mm), and early severe (soft drusen and hyperpigmentation). Participants with late AMD were defined as late neovascular (choroidal neovascularisation), late atrophic (geographic atrophy) or late mixed with signs of late neovascular and late atrophic. Grading of AMD was completed on both eyes with the final AMD grade determined by the worst eye.

Retinal photograph and AMD grading – Phenotyping cohort

All participants underwent full ophthalmic examinations and had full medical histories obtained, including smoking history. Color fundal photographs were taken of both eyes from participants during their first visit using a Topcon 50ex camera. These images were used to grade the severity of AMD for each participant. A modified version of the International Classification and Grading system for AMD was used.[54] Three categories of early AMD replaced the ARM category in this classification.[53] This study adhered to the tenets of the Declaration of Helsinki and was approved by Trinity College ethics committee.

TILDA biomarker assessment

Non-fasting whole blood samples were collected via venipuncture into 10 mL K2EDTA tubes (BD, Becton, Dickinson Limited, Oxford, UK) during health assessments (09:30–16:30) and transported at 2°C–8°C. Plasma and buffy coats were separated within 48 h and stored at −80°C until analysis. C-reactive protein (CRP, mg/l) was measured using a high-sensitivity immunoturbidimetric assay on Roche/Hitachi cobas c systems in an ISO-accredited clinical pathology laboratory at St James's Hospital, Dublin. All other biomarkers were analyzed using electrochemiluminescence immunoassays (MSD SECTOR Imager 6000, Meso Scale Discovery, Rockville, MD). MSD assays were performed at Central Biotechnology Services, Cardiff University, and biomarker concentrations were reported in pg/ml. Biomarker levels were log10-transformed and Z-scored, to facilitate comparisons across biomarkers.

Human tissue preparation

Human donor eyes obtained from the Iowa Lions Eye Bank (Iowa City, IA, USA) eyes were processed within 8 h of death. Briefly, anterior segments were removed and 4 full thickness incisions at 90° were made from anterior to posterior such that the posterior poles were flattened for photomicrography. Trephine punches of 8 mm diameter were collected from the macula, centered on the fovea centralis, and were either fixed *in toto* or were hemisected with half of the sample preserved for biochemical experiments (retina and RPE/Choroid flash frozen separately) and the other half was preserved in freshly generated 4% paraformaldehyde. RPE/Choroid samples used in this experiment were stored at −70°C.

Immunohistochemistry human tissue

Immunohistochemistry was performed on sucrose-cryoprotected frozen sections of paraformaldehyde-fixed human donor tissue using antibodies directed against NCR1/NKp46 and the endothelial cell binding lectin UEA-I. Each tissue section was blocked with 0.1% bovine serum albumin for 15 min, followed by a 1-h incubation with a 1:200 dilution of anti-NCR1/NKp46 antibody (Novus Biological, MAB1850-100) followed by 1:200 dilution of donkey anti-mouse conjugated to Alexa Fluor 546 (Invitrogen, Catalog number A10036) and a 1:50 dilution of fluorescein-conjugated UEA-I (Vector Labs, Catalog number FL-1061). Sections were subsequently washed, and nuclei were counter stained with 4′-6-diamidino-2-phenylindole (DAPI). After 3x additional washes, the sections were mounted and coverslipped with Aquamount and were imaged on an Olympus BX41 microscope.

Cell counting

Cells were counted using an automated cell counter. The cell counter used was the Countess 3FL Automated Cell Counter (Thermo Fisher). 10 μL of cell suspension was combined with 10 μL of HyClone Trypan Blue solution (Thermo Fisher) to allow for the identification of live versus dead cells. This solution was loaded onto the Countess Reusable Slides and slide was inserted into instrument. Cells were confirmed to be in focus using the image on screen, cells were then counted and dilution calculator was used to determine plating density.

Cell harvesting

For adherent cells (e.g., HRMECs), when cells had reached confluency and were due to be split or used for experimentation, the culture medium was aspirated off and the cells were washed with PBS (Sigma Aldrich) to remove any traces of cell media. PBS was removed and Trypsin-EDTA (Gibco) was added to the cells. They were placed back into incubator for 5 min in order to dissociate them from the plate they were adhered to. A light tapping of the plate/flask containing the cells encouraged them to lift. When all cells were lifted, the trypsin was deactivated by the addition of cell media that contained 10% fetal bovine serum (FBS) (Sigma). The cell solution was collected and spun in a centrifuge at 300 x *g* for 5 min so that the cells would pellet at the bottom of tube. The cell media was poured off and the pellet was resuspended in 1 mL of culture media.

Human lymphocyte characterisation & NK cell phenotyping - Flow cytometry

Human PBMCs were isolated as described above. PBMCs were blocked for 20 min at 4°C in PBS with 1% FCS and 5% human AB serum. Cell were washed x 3 in PBS and incubated with 1/1000 dilution of LIVE/DEAD Fixable Aqua (Thermofisher Scientific) for 10 min at room temperature. Cells were washed with PBS x 3 and surface stained for 20 min at 4°C with saturating concentrations of antibodies against CD56 (HCD56), CD3 (SK7/UCHT1), CD57 (HNK-1), CD4 (RPA-T4) and CD8 (SK1). Cells were subsequently washed, fixed and permeabilised using Fixation/Permeabilization Kit (eBiosciences) before being stained intracellularly with Ki67 (B56) and γH2Ax (2F3). Samples were acquired on a BD Fortessa flow cytometer and analyzed using FlowJo v10.8–10 Software. The gating strategy is shown in Figure S2B.

liCNV model

Wild-type C57Bl/6J mouse pupils were dilated with 1% tropicamide and 2.5% phenylephrine and anesthetized with ketamine/medetomidine (100/0.25 mg/kg). For experiments with IL-18 administration, CNV was induced with a green 532 nm Iridex Iris laser (532 nm, 140 mW, 100 ms, 50-mm spot size, seven spots per eye) incorporating a microscopic delivery system. PBS or 100 mg/kg murine IL-18 (GSK) was injected daily by intraperitoneal injection, with injections starting the day before laser induction. Mice were sacrificed 3 days post laser induction for scRNA-seq experiments or 7 days post laser for CNV volume analysis. For all other experiments, liCNV was carried out using the Micron IV platform (532 nm, 300 mW, 100 ms, 50 mm spot size, 3–4 spots per eye). Males were used in the scRNA-seq analysis, while male and female mice were used for immunohistochemistry and flow cytometry analysis.

Immunohistochemistry mouse tissue

For RPE/choroid flatmounts, mouse eyes were harvested, and the lens and cornea were carefully removed. The neural retina was gently removed from the RPE/choroid. Four evenly spaced incisions were made into the RPE/choroid to flatten the tissue. RPE/choroid flatmounts were fixed in 4% paraformaldehyde for 15 min at room temperature. After three PBS washes flatmounts were permeabilized and blocked with 5% NGS and 0.05% Triton X-100 in PBS for 1 h at room temperature. RPE/choroid flatmounts were incubated overnight at 4°C with anti-perforin antibody (1:250, Proteintech, 14580-1-AP) in 5% NGS and 0.05% Triton X-100 in PBS. After three PBS washes, RPE/choroid flatmounts were incubated with goat anti-rabbit Alexa Fluor 647 (1:500, Abcam, ab150079) and Isolectin B4-AlexaFluor 568 (1:300, Invitrogen, I21412) in 5% NGS and 0.05% Triton X-100 in PBS for 2 h at room temperature. RPE/choroid flatmounts were counterstained with Hoechst 33342 (1:10,000) and washed three times with PBS. RPE/choroid flatmounts were mounted onto slides with Hydromount (VWR) mounting medium and analyzed using a Leica SP8 confocal microscope.

Digestion of RPE/choroid/sclera, retina & spleen

Mouse eyes were enucleated, and the cornea and lens were carefully removed. The retina and RPE/choroid/sclera were separated by detaching the optic nerve and gently removing the retina from the RPE/choroid/sclera. Retina and RPE/choroid/sclera samples were digested by incubation in digestion buffer (1 mg/mL collagenase A, 0.1 mg/mL DNAse I, 10 mM HEPES, 5% FBS in Hank's Balanced Salt Solution) for 40 min at 37°C and were vortexed briefly every 10 min. Cell suspensions were pipetted gently to disrupt any remaining clumps. Cell suspensions were passed through a 70 mm cell strainer, centrifuged for 5 min at 200 x *g* and resuspended in 0.04% bovine serum albumin (BSA) in PBS. Splenocytes were isolated by passing spleens through a 70 mm cell strainer and rinsing with 5 mL RPMI (Sigma). Cells were centrifuged at 300 x *g* for 5 min. Pellets were resuspended in 5 mL of red blood cell lysis buffer and incubated at room temperature for 5 min. Cells were centrifuged at 300 x *g* for 5 min and supernatants were discarded.

Single cell RNA-sequencing

For scRNA-seq analysis eight mouse eyes were pooled and cells were stained with PI and DRAQ5 and gated for PI negative and DRAQ5 positive and sorted on the BD FACSAria Fusion flow cytometer. scRNA-seq was performed using the 10X Genomics Chromium Single Cell 3' v3 platform. $PI^{-}DRAQ5^{+}$ sorted from RPE/choroid tissue were resuspended in 0.04% BSA in PBS and loaded onto the Chromium controller. Chromium Single Cell 3′ v 3 reagents were used for library preparation according to the manufacturer's protocol. Fastq files of the Voigt et al.[12] human scRNA-seq dataset analyzed in this study were downloaded from the Sequence Read Archive, accession number PRJNA560646.

Analysis of scRNA-seq data

Single-cell sequence data from 10X Genomics were aligned and quantified using the Cell Ranger Single-Cell Software Suite (Version 5.0.0) against either the GRCm38 mouse or GRCh38 human reference genome. Cell Ranger was used to carry out initial quality control, followed by cell filtering, normalization and unsupervised analysis using Scanpy (Version 1.10.2).[55]

In the mouse dataset a cell was included in downstream analyses if it met the following criteria: (1) detection of more than 1,000 genes, (2) less than 10% of transcript counts originating from mitochondrial genes and (3) a total UMI count between 1,500 and 50,000. This resulted in a dataset of 14,566 genes across 1,398 cells (Conditions - PBS: 534; IL-18:864), which were used for further analysis.

In the human dataset, a cell was included in downstream analyses if it met the following criteria: (1) detection of more than 300 genes, (2) less than 20% of transcript counts originating from mitochondrial genes, and (3) a total UMI count between 500 and 50,000. This resulted in a dataset of 23,274 genes across 3,744 cells (Conditions - Donor 1: 1,153; Donor 2: 1,488; Donor 3: 1,103), which were used for further analysis. The healthy vs. disease analysis focused on peripheral RPE/choroid tissue only derived from Donor 1 (healthy) and Donor 2 (nAMD) resulting in a dataset of 16,687 genes across 1,237 cells.

Potential doublets were detected and removed using Scrublet.[56] The function scanpy.pp.normalize_total was used to normalize genes based on library sequencing depth followed by log transformation. To score the cell cycle phase of each cell we used the scanpy function sc.tl.score_genes_cell_cycle, which calculated a cell cycle phase score based on previously published marker genes.[57] Single cells with high expression of G2M- or S-phase markers were classified as G2M- or S-phase cells, respectively, while those lacking expression of both marker categories were assigned to the G1 phase. Scanorama (Version 1.7.4)[58] was used to integrate across conditions. Cells were clustered using the unsupervised graph-based clustering algorithm Leiden. The cluster-specific marker genes as well as DEGs across conditions were identified by applying the scanpy.tl.rank_genes_groups function using the Wilcoxon method and default parameters.

OCR and ECAR measurement

An XF-24 Extracellular Flux Analyzer (Seahorse Bioscience) was used for real-time analysis of the extracellular acidification rate (ECAR) and oxygen consumption rate (OCR) of NK cells cultured under various conditions. In brief, purified NK cells were adhered to CellTaq (BD Pharmingen) coated XF 24-well microplate (Seahorse Bioscience) at 750,000 cells per well, 10^7 cells/ml. Sequential measurements of ECAR and OCR following addition of the inhibitors (Sigma-Aldrich) oligomycin (2 mM), rotenone (100 nM) plus antimycin (4 mM) and 2-deoxyglucose (2DG) (30 mM) allowed for the accurate calculation of oxygen consumption due to OxPhos and acidification due to glycolysis.

Stimulation of cells

For cell stimulation, PBMCs or splenocytes were stimulated for 18–24 h with 25–100 ng/mL human or murine IL-18 (BioTechne, GSK), 30 ng/mL human IL-12 (Miltenyi) or 10–20 ng/mL murine IL-12 (BioTechne), 20–100 ng/mL IL-15 (Miltenyi). For experiments to measure NK cell cytotoxicity versus targets, NK-92MI or PBMCs were seeded in u-bottom 96 well plates at desired concentrations and either remained unstimulated, or were stimulated with combinations of IL-12 (10 ng/mL), IL-15 (100 ng/mL) and IL-18 (100 ng/mL) overnight (12–18 h).

CD107a & IFN-γ assay (human)

To assess NK cell cytotoxicity against targets, NK-92MI/PBMCs were stimulated as detailed above and incubated overnight. Prior to co-culture of NK-92MI/PBMCs and targets, HRMECs (normal or senescent) were passaged and seeded into a flat bottom 96 well plate at a density of 0.5×10^6 cells/ml and left overnight. NK-92MI/PBMCs were added to target cells at either 1:1 (HRMECs) or 1:10 (721.221s) effector to target ratios. Anti-human CD107a APC-conjugated antibody was added to each well before centrifugation at 150 x *g* for 5 min and incubation for 1 h, after which Monensin (MSC) and Brefeldin (eBiosciences) were added to each well. Cells were incubated for a further 3 h before being transferred to a fresh round-bottom 96 well plate for flow staining. Cells were blocked in PBS/1% FBS with 10% normal human serum and washed in PBS. Cells were stained extracellularly with Live/Dead aqua (Biosciences), anti-human CD3 (UCHT1, FITC conjugated) and anti-human CD56 (HCD56, Pe-cy7 conjugated). Cells were fixed and permeabilised using Fixation/Permeabilization kit (eBiosciences), before being stained intracellularly with anti-human IFN-γ (B27, PE-conjugated), antibody list in Figure S7C. Samples were acquired on a BD FACSCanto flow cytometer and analyzed in FlowJo v10.8.10 software.

Mouse lymphocyte characterisation and phenotyping

Anti-CD45 PE (30-F11) was administered by intravenous injection 5 min prior to sacrificing animals to assess circulating versus tissue-resident lymphocyte subsets. Cells were isolated from the spleen and RPE/choroid of the eye as described above and surface stained using the following antibodies: CD45 (30-F11), NK1.1 (PK136), CD3 (17A2), CD4 (GK1.5), CD8 (53–6.7), CD69 (H1.2F3), and CD25 (PC61.5). Cell were fixed and permeabilized and stained for the intracellular marker Ki67 (SolA15). A full antibody list can be found in Figure S3A. Samples were acquired on a BD Fortessa flow cytometer and analyzed using FlowJo v10.8–10 Software.

IFN-γ assay (mouse)

Splenocytes were isolated from 6 to 10-week-old C57Bl/6J mice and stimulated for 18 h as described above. In the last 4 h of stimulation, Golgi-Plug (BD Pharmingen) was added to the cells. Cells were stained extracellularly with anti-mouse CD3 (17A2),

CD69 PE-Cy5 (H1.2F3) and NK1.1 (PK136, Invitrogen). Cells were permeabilised and fixed using BD Cytofix/Cytoperm kit as per manufacturer's instructions and stained intracellularly with anti-mouse IFN-γ (XMG1.2, Biolegend). Samples were acquired on a BD Cyan ADP flow cytometer and analyzed using FlowJo v10.8–10 Software.

RNA extraction

RNA was isolated from cells using the E.Z.N.A Total RNA Kit I (Omega) according to the manufacturer's instructions. 350 μL TRK Lysis buffer (Bioline) was applied to samples (either cells or retinas) and the lysate was collected and deposited into a HiBind RNA Mini Column in a collection tube. An equal volume of 70% ethanol was also added to the tube. All tubes were spun at 10,000 x *g* for 1 min. The filtrate in the collection tube was poured off and 500 μL of the RNA Wash Buffer I was added to each column and all tubes were spun at 10,000 *x g* for 30 s. The filtrate in the collection tube was poured off and 500 μL RNA Wash Buffer II was added to each column and the tubes were spun at 10,000 x *g* for 1 min. This process was repeated twice. After this, the empty column and collection tubes were spun at maximum speed for 2 min. The column was then put onto a 1.5 mL Eppendorf tube and 31 μL NFW was added to elute the isolated RNA from the column. These tubes were spun at maximum speed for 2 min. The eluted RNA was transferred back into the column and spun for a further 2 min. After this the RNA was kept on ice and the yield (ng/μL) and the quality (A260/A280) was measured by using a Nanodrop 1000 spectrophotometer (Thermo Fisher). RNA was stored at −80°C until future use.

cDNA preparation

The isolated RNA was diluted so all samples were at the same concentration. The master mix for the cDNA synthesis contained 0.25 μL MMLV Reverse Transcriptase (Promega), 2 μL 5X MMVL Buffer (Promega), 2 μL 10 nM dNTPs (New England Biolabs), 0.5 μL Random Hexamers (IDT) and 0.25 μL RNase Out (Invitrogen). Equal volumes of master mix and RNA were added to each tube (5 μL) and run on a thermocycler. Once synthesised the cDNA was diluted to 10 ng using NFW and stored at 4°C. cDNA synthesis thermocycling conditions were as follows: Annealing at 20°C for 10 min; Elongation at 42°C for 40 min and Enzyme inactivation at 95°C for 3 min.

Real time reverse-transcription polymerase chain reaction (RT-qPCR)

RT–qPCR was carried out used the SensiFAST SYBER HiRox Kit (Bioline) and all samples and all controls were run in triplicate. The master mix was comprised of SYBR and the forward and reverse primers for the target genes. RPLP0 was used as the house keeping gene for all experiments. 8 μL of the master mix was added to each well of a 0.1 mL MicroAMP Fast 96 well reaction plate (Applied Biosystems). Then 2 μL of the cDNA of each sample was added and the whole plate was sealed with optical adhesive film (Applied Biosystems). 10 ng of cDNA was loaded into each well. This plate was then centrifuged at 300 x *g* for 1 min and ran on a QuantStudio 5 real-time PCR machine (Applied Biosystems). A melt curve step was added to check the stability and specificity of the primers and the comparative Ct (ΔΔCt) method was used. All the samples were normalised to the house keeping gene, then the sample ΔCtwas normalised to the ΔCtof the untreated housekeeping samples. The relative fold change of the samples divided by the housekeeping sample control was calculated using the formula 2^{-} ΔΔCt.

Primers used for RT-qPCR found in Figure S7A). Thermocycling conditions for RT-qPCR are as follows: Holding at 50° for 2 min followed by 95° for 10 s; Cycling stages (40 cycles) of 95° for 15 s and 60° for 1 min. This was followed by melt curve generation at 95° for 1 s, 60° for 20 s and 95° for 1 s.

Caspase 3/7 incucyte assay

HRMECs were seeded in a 96 well plate at 1×10^5 cells/ml and allowed form a monolayer overnight. NK-92MI were added in a 1:1 Effector to Target Ratio. As a positive control for apoptosis, staurosporine was added to HRMECs at a final concentration of 250 nM. Incucyte Caspase 3/7 Green Dye (Item No. 4440) was added to each well at a final concentration of 2.5 μM. The 96 well plate was centrifuged at 150 x *g* for 3 min to bring the cells to the same focal plane. The plate was added to the Incucyte S3, and images were taken at 10x using the Phase and Green channels every 30 min over the course of six hours. At each timepoint, the number of caspase 3/7 objects and total integrated intensities of the green signal from each well was reported using Incucyte 2024b Software.

MTS/PMS assay

Media was removed from cells, and 20 μL of the CellTiter Aqueous One Solution MTS/PMS assay (Promega) and 100 μL of cell media was reverse pipetted into each well. The plate was covered with tinfoil and incubated at 37°C with 5% CO2 for 1 h. The plate's absorbance was then read at 490 nm and 680 nm on a SynergyMX plate reader (BioTek, Gen5). Background absorbance (680 nm readings) was subtracted from the 490 nm absorbance readings to get the true absorbance values. The average of the untreated samples was calculated, and all other samples were normalised to this average of the untreated samples in order to determine their viability expressed as a percentage.

Lactate dehydrogenase (LDH) assay

Cellular cytotoxicity of HRMECs, following co-culture with NK-92MI, was assessed using the CyQuant LDH Cytotoxicity assay as per manufacturer instructions. Briefly, 10 μL of Cyquant 10x Lysis buffer was added to lysis wells of HRMECs for 45 min. After 45 min, 25 μL of the supernatant of each well was transferred to a second 96 well plate, with 50 μL of Cyquant LDH substrate mix, and was

placed on a plate shaker, covered. After 30 min, the absorbance of each well was measured at 490 nm using a SynergyMX plate reader (Biotek). Relative lytic cell death, compared to the cell lysis control, was calculated as follows:

$$\% \; Cell \; Death = \frac{A_{sample} - A_{unstimulated}}{A_{lysis} - A_{unstimulated}}$$

Where: A is the corrected absorbance of each well, calculated by subtracting 680 nm readings from 490 nm readings.

Adoptive transfer models

NK cells were isolated from spleens of adult CD57Bl/6J mice (aged 8–10 weeks) using an NK Cell Isolation kit (Miltenyi) as per manufacturer's instructions. NK cells were activated with IL-12 (20 ng/mL), IL-15 (20–100 ng/mL, Peprotech) and IL-18 (100 ng/mL, GSK) overnight (for 12–18 h). Activated NK cells (1×10^5 cells/mouse) were transferred IV via tail vein injection directly into C57Bl/6J mice (aged 8–10 weeks) immediately prior to liCNV induction or alternatively into JR5558 mice (aged 6 weeks), a model of aberrant retinal angiogenesis.

Cryosectioning

After sacrifice, the mice eyes were removed using a forceps. They were placed in an Eppendorf of Pierce 16% Formaldehyde (w/v), Methanol-free (PFA) that was diluted to 4% in PBS. Eyes were left to fix at room temperature with gentle agitation for an hour and a half. Following fixation, eyes were washed in PBS three times with 5 min between each wash. Using a microdissection microscope, the cornea and lens were removed using a fine forceps and dissection scissors (World Precision Instruments). To prepare the tissues for cryosectioning, the eyes were cryoprotected by immersion in a 20% sucrose solution for 1 h, followed by incubation in a 30% sucrose solution overnight at 4°C. After cryoprotection, the eyes were embedded in optimal cutting temperature (OCT) compound (Fisher Scientific) within plastic molds. The molds containing the embedded eyes were frozen using a liquid nitrogen and isopropanol bath to ensure slow and controlled freezing. Cryosections were cut at a thickness of 12 μm using a Leica CM1900 cryostat. The sections were collected onto poly-L-lysine-coated slides and allowed to dry. Once dried, the slides were stored at −20°C for subsequent analysis.

RPE flatmount dissection and staining

Mice were sacrificed and their eyes were removed using a forceps. The eyes were placed in ice-cold PBS and dissected as quick as possible. Dissection was performed under a microdissection microscope using a fine forceps and dissection scissors to isolate the posterior eye cup. Excess muscle tissue was carefully removed from the exterior of the eyeball, and the optic nerve was severed. A small incision was made at the ora serrata, followed by the removal of the anterior segment of the eye, including the cornea, iris, and lens, by dissecting along the circumference around the pars plana. The retina was carefully separated from the posterior eye cup and this was transferred to an Eppendorf and snap frozen in liquid nitrogen. This was stored in the −80°C freezer for future analysis. Removing the retina exposed the RPE, choroid, and sclera complex. The RPE/choroid/sclera complex was radially cut in four places at 90°C intervals to flatten the tissue. The flat-mounted tissues were fixed in ice-cold methanol for 15 min over ice and subsequently washed three times in PBS. Following fixation, the RPE flatmounts were blocked and permeabilized in a solution of 5% NGS and 0.05% Triton X-100 for 1 h at room temperature. The tissues were then incubated overnight at 4°C with primary antibodies. After primary antibody incubation, the flatmounts were washed three times in PBS and then incubated for 2 h at room temperature protected from light with secondary antibodies diluted in 5% NGS and 0.05% Triton X-100 in PBS. The flatmounts were washed again 3 times (15 min each) in PBS and counterstained with the nuclear stain DAPI (1:10000 dilution in PBS) for 5 min at room temperature away from direct light. Following this, the flatmounts were washed in PBS three times and mounted onto slides with the RPE facing up. Care was taken to make sure all sides of the flatmount were lying as flat as possible without touching the RPE itself. Hydromount was applied to coverslips and they were lowered onto the flatmount at a 45° angle to prevent bubble formation. Stained slides were stored at 4°C until they were imaged using confocal microscopy. Secondary-only controls were performed for all immunostaining experiments to confirm antibody specificity (Figure S4B).

Immunofluorescent staining of cryosections

Slides that had been stored at −20°C were selected for staining. A few sections on each slide were selected and drawn around with a hydrophobic barrier pen (PAP pen, Sigma). This prevented the waste of valuable reagents by keeping liquid pooled in a small droplet. Slides were rehydrated at room temperature for 10 min in PBS. To block nonspecific binding and permeabilize the tissue, sections were incubated in a blocking solution containing 5% NGS and 0.05% Triton X-100 (Sigma) in PBS for 1 h at room temperature. Following blocking, the sections were incubated overnight at 4°C in a humidity chamber with primary antibodies diluted in PBS containing 1% NGS and 0.1% Triton X-100. The next day, the sections were washed three times with PBS to remove unbound primary antibodies. The sections were then incubated with appropriate fluorescent secondary antibodies diluted in 5% NGS and 0.05% Triton X-100 in PBS. This was carried out at room temperature in the humidity chamber for two hours. After secondary antibody incubation, sections were washed again three times with PBS. Finally, the sections were counterstained with DAPI (1:10000 diluted in PBS). They were then washed with PBS and hydromount was added to coverslips which were lowered onto sections at an angle to

avoid bubbles. Slides were stored in the dark at 4°C until imaging. Secondary-only controls were performed for all immunostaining experiments to confirm antibody specificity.

Confocal microscopy

A three-laser confocal microscope (Leica SP8 scanning confocal) was used to image the cryosections and RPE flatmounts used in this project. This microscope was used to perform both tile-scans of the whole sections and Z-stacks for a more in-depth analysis of certain regions. The retinal sections were placed on the microscope stage, and the appropriate laser were selected based on the fluorophores used (405 nm for DAPI, 488 nm for Alexa Fluor 488, 594 nm for Alexa Fluor 594). Imaging was performed with 20x objective for tile-scans and 40x oil immersion for Z-stacks. The pinhole was set to 1 Airy unit to ensure optimal optical sectioning. For tile-scans, the zoom was set to 0.75 and for Z-stacks, the zoom was set to 1. Bidirectional scanning was selected to allow for faster image acquisition. To image the entire retinal section, the tile-scan function in the Leica LAS X software was used. A grid of adjacent fields was defined to cover the region of interest, ensuring overlap between adjacent tiles for accurate stitching during image processing. The z stack function was enabled to capture images at multiple focal planes if necessary. After scanning, the individual tiles were stitched together automatically using the LAS X software to generate a composite image of the entire retinal section. All images were saved in high-resolution formats for subsequent analysis. To obtain Z-stacks, the Leica LAS X software was used to define the upper and lower boundaries of the z axis (optical depth) within the tissue section. The microscope automatically acquired images at regular intervals along the z axis to capture a series of optical sections through the entire thickness of the tissue. The step size between each Z-plane was set to 1 step size for each section. Once all Z-planes were captured, the individual optical sections were combined to create a three-dimensional reconstruction of the retinal layers using the LAS X software. Images were saved in high-resolution formats for further analysis. Secondary-only controls were performed for all immunostaining experiments to confirm antibody specificity.

QUANTIFICATION AND STATISTICAL ANALYSES

To assess the association between circulating inflammatory biomarkers and the presence of any AMD, separate binomial logistic regression models were fitted for each biomarker. The outcome variable was binary (AMD vs. No AMD), with independent variables being the standardized biomarker level (log10, *Z* score), adjusted for age, sex, and smoking status.

To investigate the association between AMD severity and inflammatory biomarker levels, separate linear regression models were fitted for each biomarker. The independent variable was AMD status (multi-level categorical variable: No AMD [reference value], Early Mild, Early Moderate, Early Severe, and Late AMD), with the dependent variable being the standardized biomarker level. Models were adjusted for age, sex, and smoking status. All statistical analyses involving TILDA data were conducted using R statistical software (version 4.3.1).

All other data was analyzed with GraphPad Prism software. Normality testing was carried out using Shapiro-Wilk, Kolmogorov-Smirnov, and D'Agostino & Pearson omnibus normality testing. When datasets were found to follow a nonnormal distribution a Kruskal-Wallis with Dunn's multiple comparison test or Mann-Whitney U test was carried out. Statistical analysis on normally distributed datasets was using one-way ANOVA with Dunnett's post-test or Student's *t* test. The statistical approaches were all deemed to be valid for each individual experiment. Outlier testing was carried out using ROUT (Q = 1%) and removal of outliers would not have changed statistical significance, so all samples were included.

Cell Reports Medicine, Volume *7*

Supplemental information

Circulating natural killer cells
are phenotypically and functionally altered
in age-related macular degeneration

Kiva Brennan, Ema Ozaki, Eleanor Noone, Sarah Palko, Kieran P. Byrne, Fiona Roche, Matt McElheron, Kieva Byrne, Luke Gibbons, Katie Robb, Said Aktas, Emma Connolly, Natalie Hudson, Matthew M. O'Riordan, Dara O'Boyle, Rachel Dalton, Aline Zoller, Erin Fahey, Karsten Hokamp, Derrick Feenstra, Nollaig Bourke, Matthew Campbell, David Finlay, Kelly Mulfaul, Robert F. Mullins, Rose Anne Kenny, Mark T. Cahill, and Sarah L. Doyle

A

Supplementary Table 1: Demographics of TILDA cohort with AMD severity

	AMD Severity						
	**No Disease**	**Early Mild**	**Early Moderate**	**Early Severe**	**Late AMD**	**Total**	**P Value**
n (%)	4,169 (95.6%)	78 (1.8%)	81 (1.9%)	27 (0.6%)	8 (0.2%)	4,363 (100.0%)	
***Age,** mean(SD)*	60.5 (8.5)	60.4 (7.6)	65.1 (8.7)	65.3 (9.1)	67.0 (11.2)	60.6 (8.5)	<0.001
***Sex,** n (%)*							
Male	1,841 (44.2%)	31 (39.7%)	34 (42.0%)	12 (44.4%)	4 (50.0%)	1,922 (44.1%)	0.929
Female	2,327 (55.8%)	47 (60.3%)	47 (58.0%)	15 (55.6%)	4 (50.0%)	2,440 (55.9%)	
***Smoking,** n(%)*							
Never/Past	3,533 (84.8%)	65 (83.3%)	77 (95.1%)	24 (88.9%)	8 (100.0%)	3,707 (85.0%)	0.075
Current	635 (15.2%)	13 (16.7%)	4 (4.9%)	3 (11.1%)	0 (0.0%)	655 (15.0%)	
***BMI,** mean(SD)*	28.5 (4.9)	28.9 (5.1)	28.1 (4.8)	27.5 (3.9)	27.7 (6.1)	28.5 (4.9)	0.909
***Education,** n(%)*							
Primary/none	820 (19.7%)	14 (17.9%)	15 (18.5%)	5 (18.5%)	3 (37.5%)	857 (19.7%)	0.859
Secondary	1,791 (43.0%)	35 (44.9%)	30 (37.0%)	11 (40.7%)	2 (25.0%)	1,869 (42.9%)	
Third/higher	1,556 (37.3%)	29 (37.2%)	36 (44.4%)	11 (40.7%)	3 (37.5%)	1,635 (37.5%)	

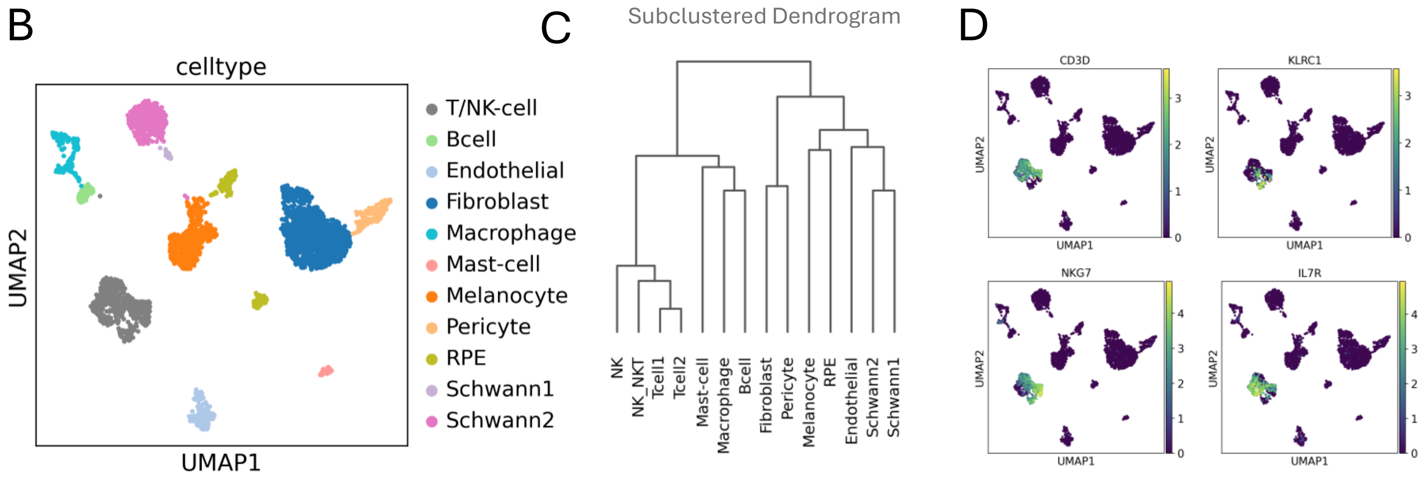

Supplementary Figure 1: Demographics of TILDA cohort, and further analysis of publicly available human donor eye dataset. Related to Figure 1.

(A) Demographics table of TILDA cohort, including disease severity. Following normality testing, Pearson's X^2 was used to compare categorical variables and Kruskal-Wallis was used to compare continuous variables between groups. **(B-D)** Reanalysis of Voigt et al. dataset [12] **(B)** UMAP plot of 3,744 cells derived from peripheral and macular ocular tissue from three human donors showing the presence of 11 cell types based on the expression of specific markers. **(C)** Dendrogram showing the relationship between annotated cell types. **(D)** UMAP plots showing expression distributions, in log scale, of four marker genes across annotated cell types.

A

Supplementary Table 2: Demographics of clinical cohort with AMD severity					
	**No Disease**	**Early AMD**	**Late AMD**	**Total**	**P Value**
***n** (%)*	33 (52.4)	17 (27.98)	13 (20.6)	63 (100)	
**Age,** *mean (SD)*	70.97 (8.64)	71.4 (9.4)	81.69 (9.89)	73.3(9.95)	0.0013
**Sex,** *n (%)*					
Male	14 (42.4%)	6 (35.3%)	6 (46.2%)	26 (36.6%)	0.8201
Female	19 (57.6%)	11 (64.7%)	7 (53.8%)	37 (63.4%)	

B

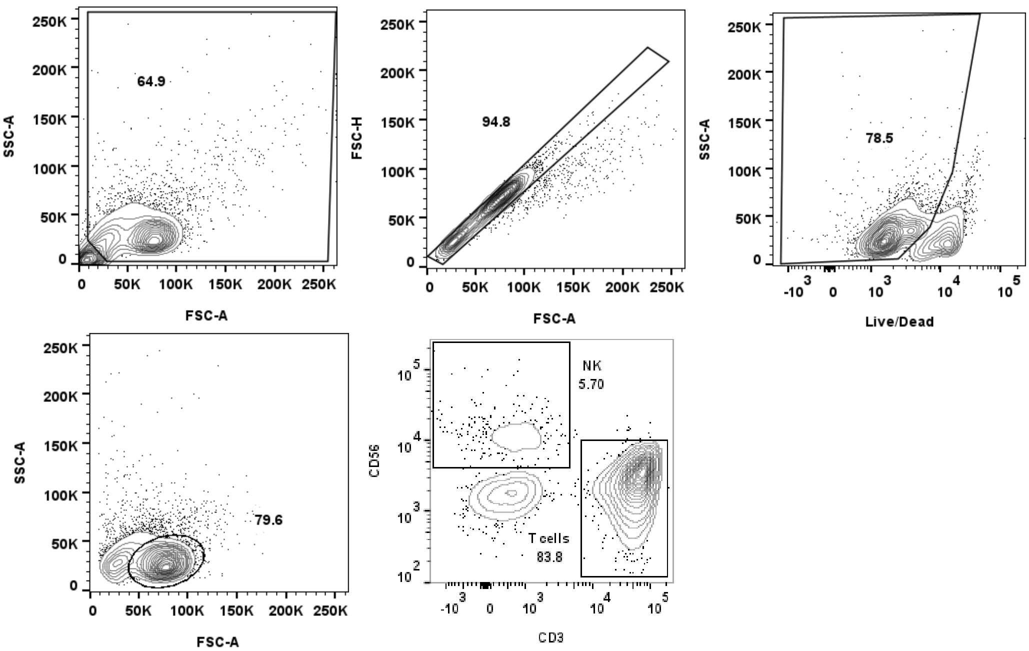

C

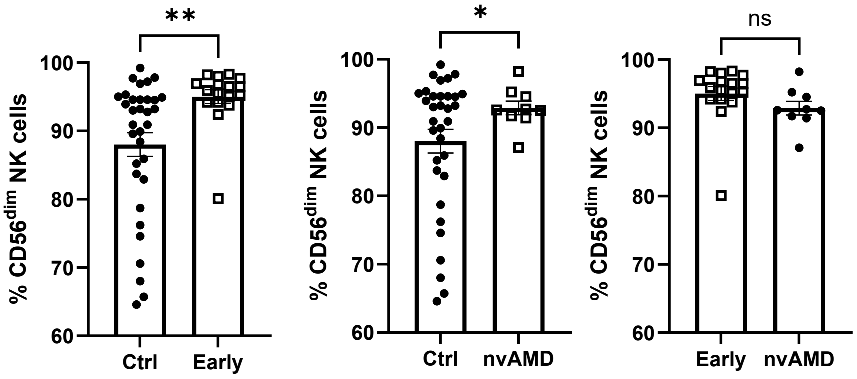

Supplementary Figure 2: Demographics and gating strategy for flow cytometric analysis of NK and T cell subsets in human PBMCs. Related to Figure 1.

(A) Demographics table of clinical sample cohort, including disease severity. Following normality testing, Pearson's X^2 was used to compare categorical variables and Kruskal-Wallis was used to compare continuous variables between groups. **(B)** Representative gating strategy for NK cells and T cells based on expression of CD56 and CD3 **(C)** CD56^{dim} NK cells expressed as a percentage of total NK cells divided according to AMD stage (n=26 Ctrl, n= 17 early AMD, n=9 nvAMD). Graphical data are presented as mean ±SEM. Following normality testing, unpaired t-tests or one-way ANOVA with Dunnett's multiple comparison tests were used to compare groups.

A

Antigen	Fluorophore	Clone	Cat no.	Supplier
CD45	BB515	30-F11	564590	BD
NK1.1	BB700	PK136	556503	BD
CD3	VioBlue	17A2	130-118-849	Miltenyi
CD4	AF700	GK1.5	100429	Biolegend
CD8	APCCy7	53-6.7	100713	Biolegend
Ki67	PECy7	SolA15	25-5698-80	Invitrogen
CD69	BV650	H1.2F3	104541	Biolegend
CD25	PE	PC61.5	12-0251-81	Invitrogen

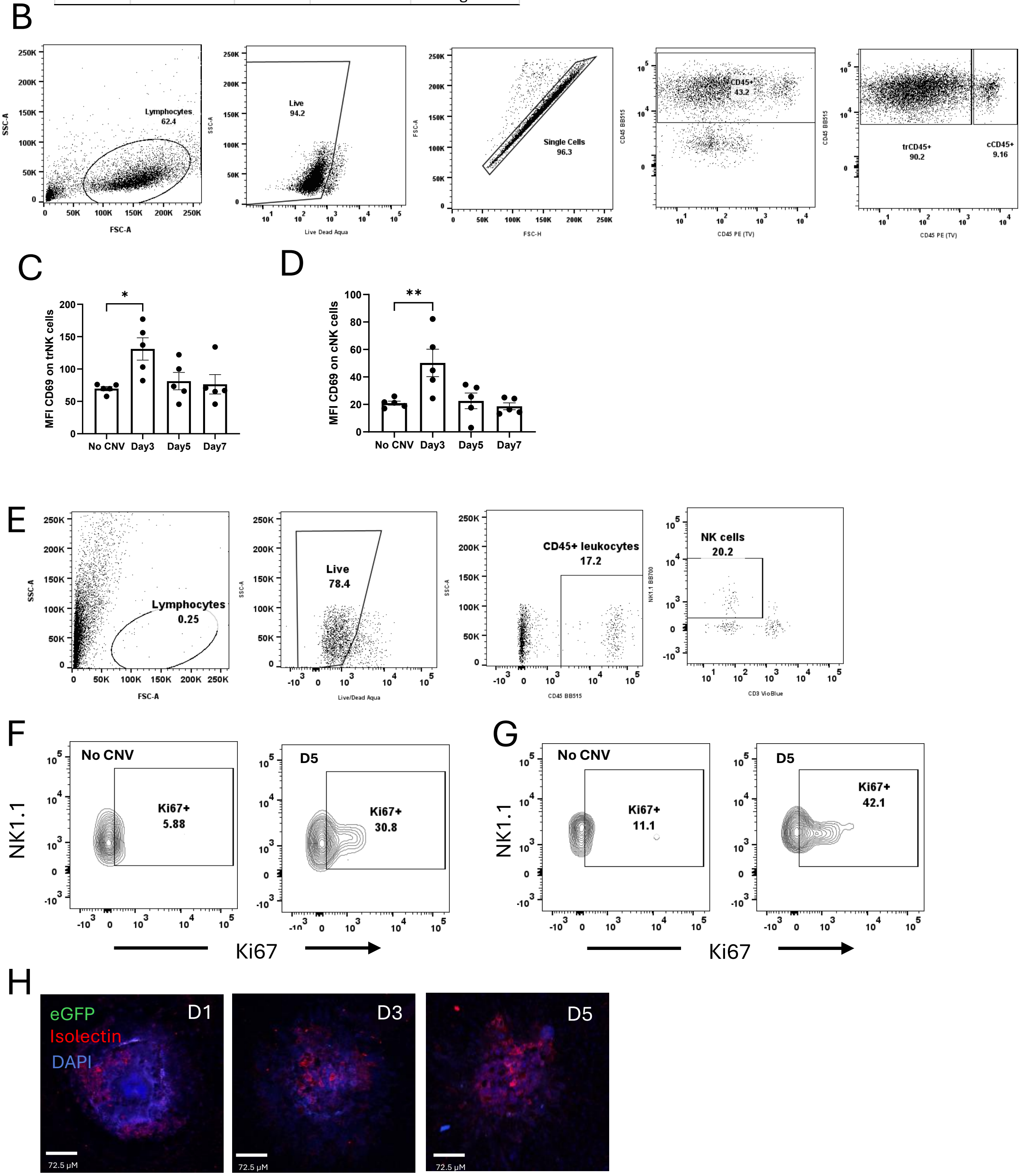

Supplementary Figure 3: Splenocyte & RPE/choroid flow cytometry staining and gating. Related to Figures 2 and 3.

(A) Flow cytometry antibodies, including fluorophore, clone, catalogue number and supplier used in Figures 2 & 3. **(B)** Gating strategy for identification of lymphocyte populations from spleen. **(C)** MFI of CD69 on tissue resident and **(D)** circulating splenic NK cells following liCNV. **(E)** Gating strategy for identification of lymphocyte populations from RPE/choroid. **(F & G)** Representative Ki67 gating for total RPE/choroid NK cells & tissue resident RPE/choroid NK cells. **(H)** Representative RPE/choroid flatmounts from NCR1-GFP^{-} mice at 1, 3 and 5 days post liCNV (2 eyes/timepoint, 2 liCNV/eye).

A

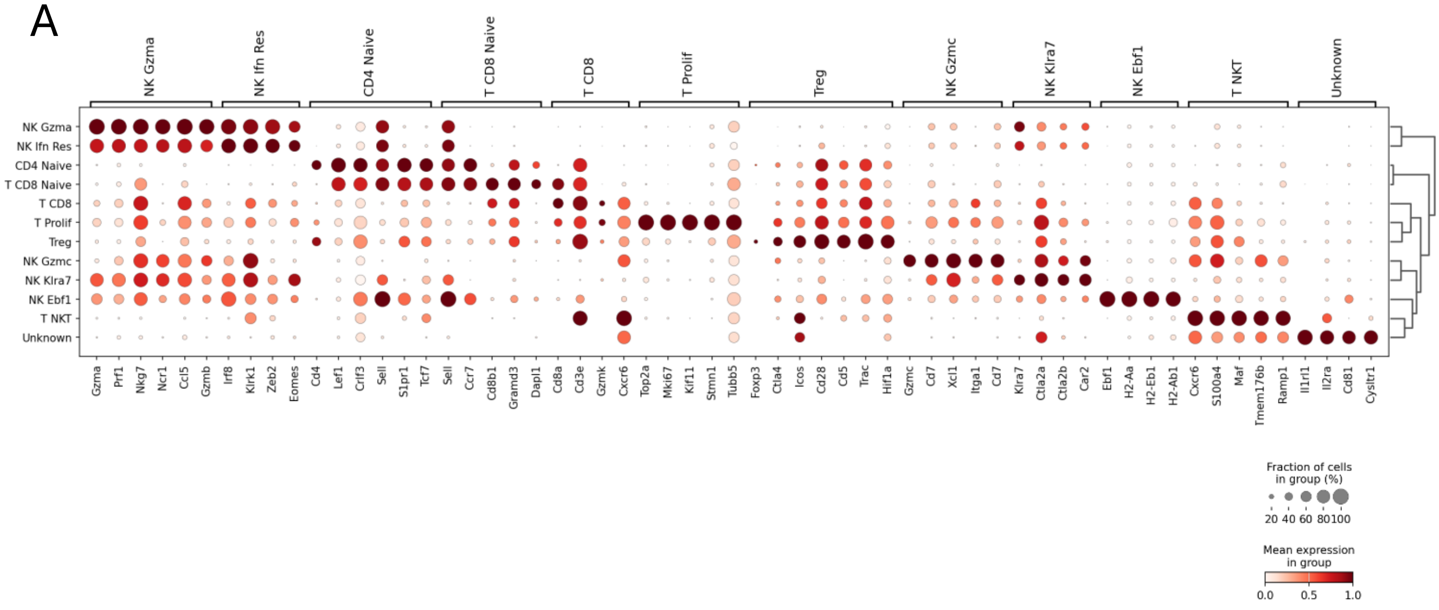

B

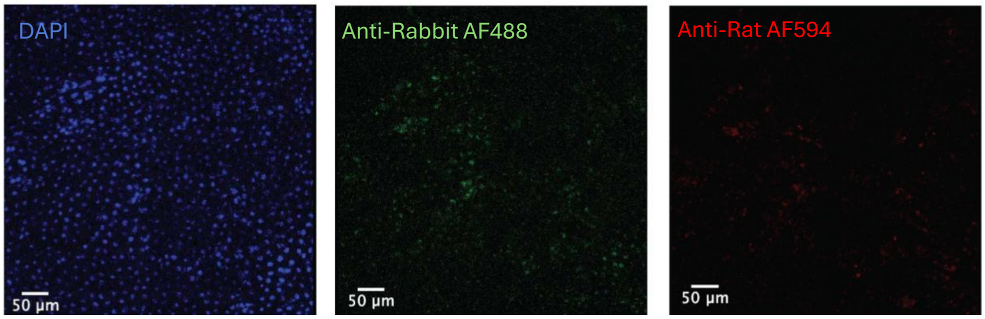

Supplementary Figure 4: scRNA sequencing analysis from healthy vs liCNV cohort and control staining for RPE/choroid flatmounts. Related to Figure 3.

(A) Dotplot showing the expression pattern of known marker genes across cell types from scRNA sequencing experiment comparing liCNV versus control RPE/choroid tissue. Dot colour indicates the average expression level within each donor. Dot size indicates the fraction of cells in each group that express a particular gene. **(B)** Staining controls for RPE/choroid flatmounts. Secondary only controls for DAPI, anti-rabbit AF488 and anti-rat AF594 on RPE/choroid flatmounts.

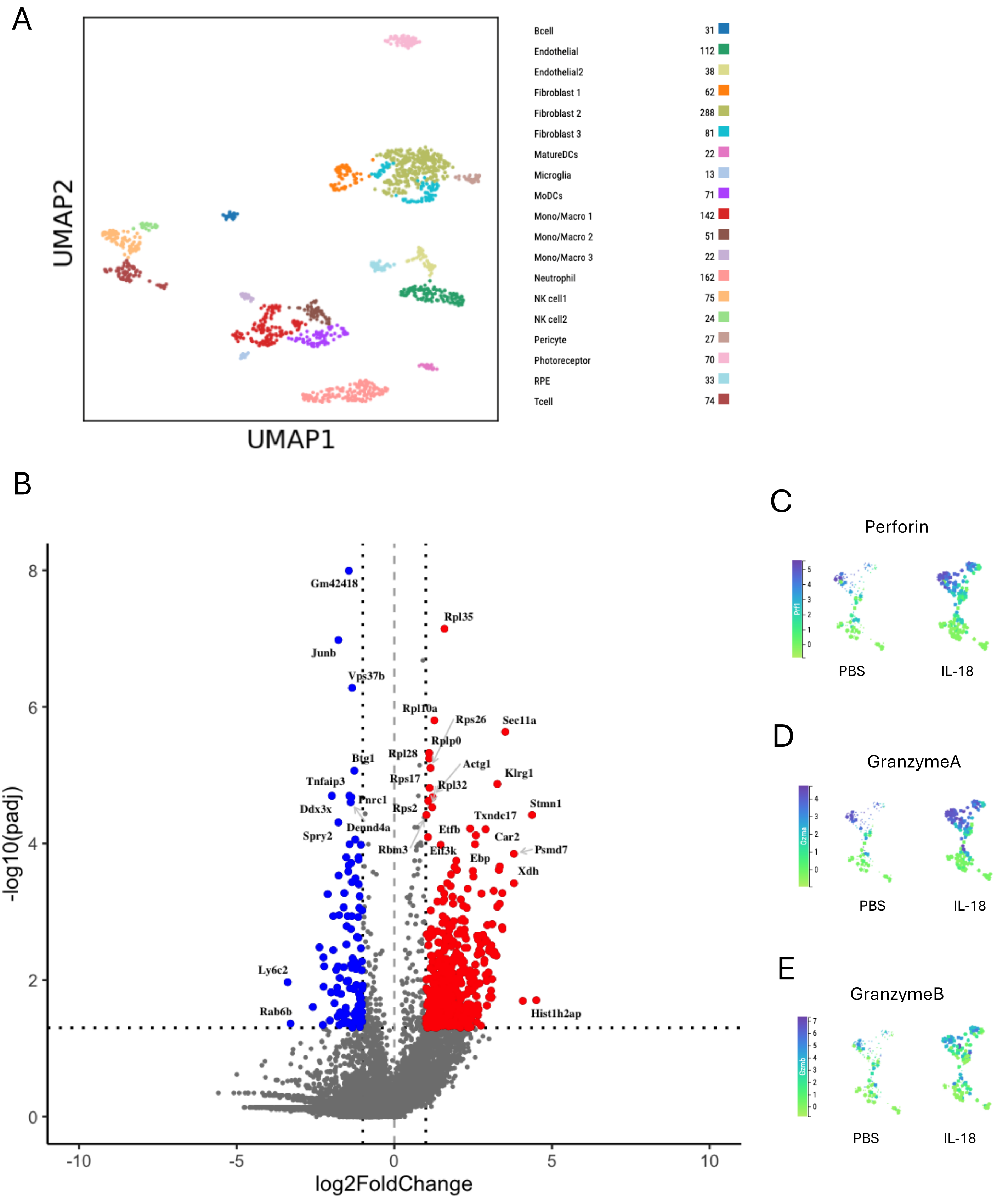

Supplementary Figure 5: Cell clustering analysis of scRNA sequencing data from RPE/choroid of PBS and IL-18. Related to Figure 4.

(A) UMAP of clusters (Scanpy analysis) of CNV tissue from vehicle and IL-18 treated mice (n=8 mice) **(B)** Volcano plot showing the genes that are differentially expressed in the NK cell group (NK1 & NK2) between IL-18 vs PBS treatment. **(C-E)** Expression of cytolytic genes *Perforin*, *GranzymeA* and *GranzymeB* in RPE/choroid of mice administered with a liCNV and treated with PBS or IL-18.

A

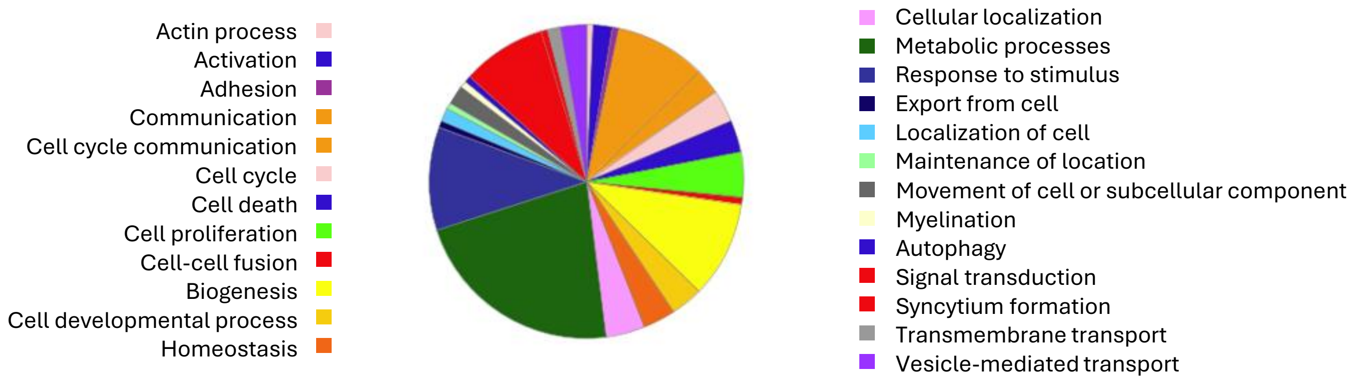

B

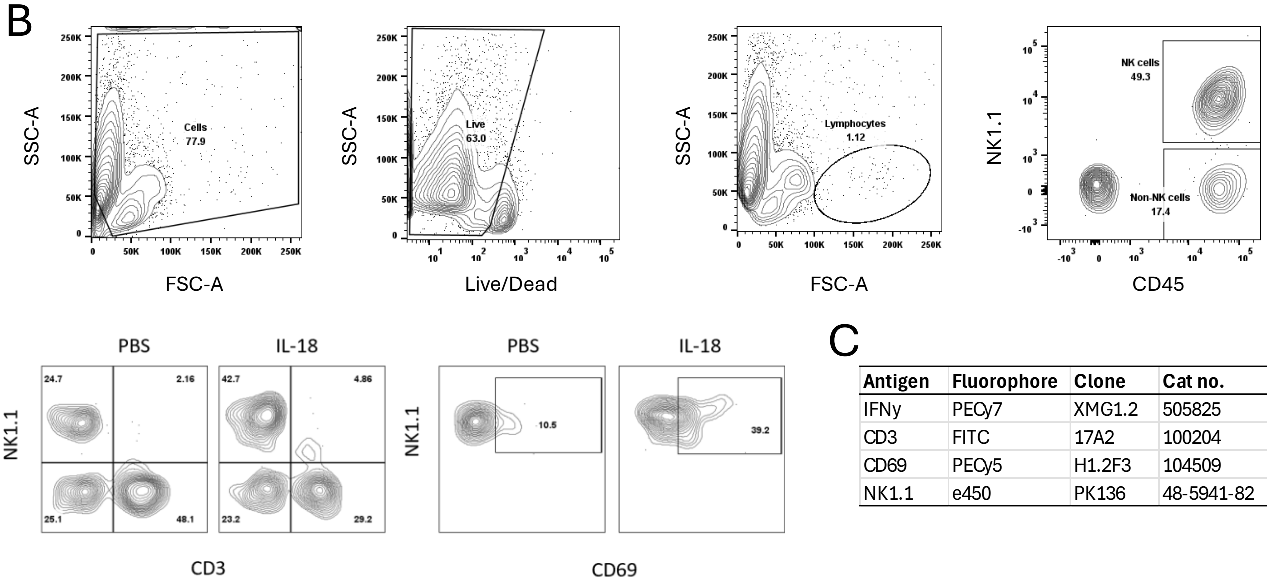

C

Antigen	Fluorophore	Clone	Cat no.	Supplier
IFNy	PECy7	XMG1.2	505825	Biolegend
CD3	FITC	17A2	100204	Biolegend
CD69	PECy5	H1.2F3	104509	Biolegend
NK1.1	e450	PK136	48-5941-82	Invitrogen

Supplementary Figure 6: Functional enrichment analysis of scRNA sequencing data from RPE/choroid of PBS and IL-18, and flow cytometry gating on RPE/choroid. Related to Figure 4.

(A) Full list of gene processes upregulated in NK2 vs NK1 cells in PBS vs IL-18 treated ocular tissue (n=8 eyes/group). **(B)** Flow cytometry gating strategy for NK cells from RPE/choroid **(C)** Flow cytometry antibodies, including fluorophore, clone, catalogue number and supplier.

A

Gene	Primer	Sequence (5'-3')
p21	For	GGCAGACCAGCATGACAGATTTC
	Rev	AACCTCTCATTTCAACCGCCT
Serpine1	For	GGTTCTGCCCAAGTTCTCCC
	Rev	CACCGTGCCACTCTCGTTCA
MicA	For	GAATCCGGCGTAGTCCTGAG
	Rev	TCCGGGGATAGAAGCTGGAA
MicB	For	CTGCCCAGACAAGACGTAGG
	Rev	CGCCTCTCCTTGGATTCCTC
ULBP2	For	TGTGCCTGAGGACATGGCGA
	Rev	CATTACTTCTCAATGGGAGACTGT
ULBP5	For	TGGACAACGGTTCATCCTGG
	Rev	ACCCCACGAGGAGGTCATT
ULBP6	For	TCCAGGATGACCTAGGGTGG
	Rev	AGGATGAAGCAGGGGAGGAT
UBC	For	ATTTGGGTCGCGGTTCTTG
	Rev	TGCCTTGACATTCTCGATGGT
RPL0	For	TGGCGCAGCCAATAGACAG
	Rev	CGCATCATGGTGTTCTTGCC

B

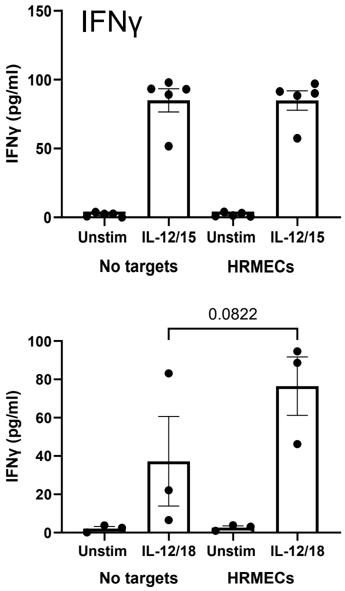

C

Antigen	Fluorophore	Clone	Cat no.	Supplier
CD56	PECy7	HCD56	318318	Biolegend
CD3	FITC	UCHT1	300406	Biolegend
CD107a	APCCy7	H4A3	328620	Biolegend
IFNy	PE	B27	506507	Biolegend

D

Disease Status	Age	Sex
Control	79	Male
Control	87	Male
Control	88	Male
CNV	79	Male
CNV	87	Male
CNV	88	Male

Supplementary Figure 7: Primer sequences for HRMEC investigations; IFNγ production from NK cells in a PBMC population in the presence of HRMECs. Related to Figure 5.

(A) Forward and reverse primer sequences for genes involved in hypoxia and senescence, and NKG2DL genes **(B)** Flow cytometry analysis of IFNγ production from PBMC NK cells in response to cytokine stimulation and incubation with HRMECs **(C)** Flow cytometry antibodies, including fluorophore, clone, catalogue number and supplier. **(D)** Characteristics of donor tissue used for Immunohistochemistry. Graphical data are presented as mean ±SEM. Following normality testing, unpaired t-tests or one-way ANOVA with Dunnett's multiple comparison tests were used to compare groups.

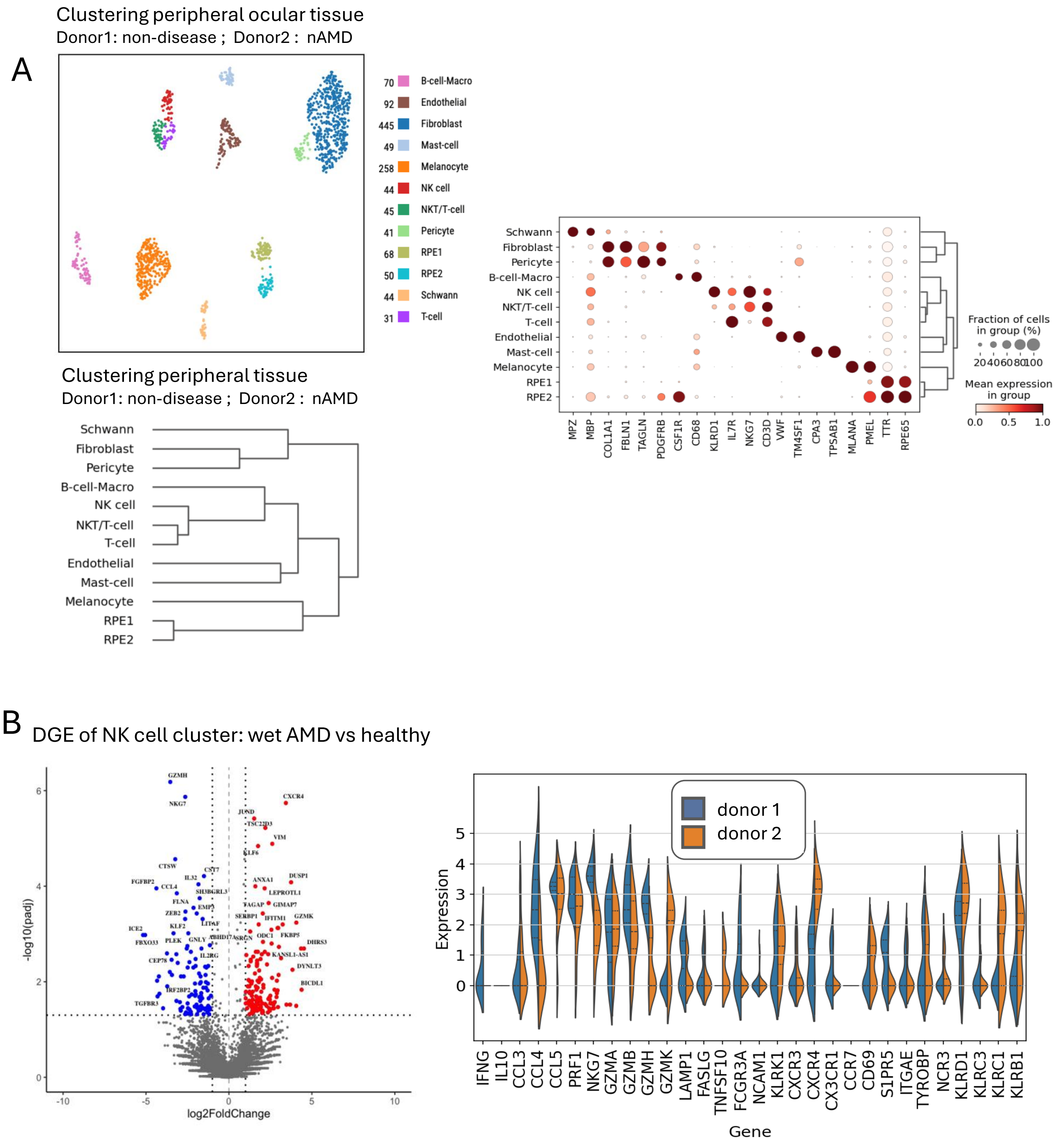

Supplementary Figure 8: Analysis of scRNA sequencing data from non-disease and nAMD donor ocular tissue. Related to Figure 6.

(A) UMAP plot, dendrogram and dot plot analysis of 1,237 cells derived from peripheral ocular tissue from two human donors (nAMD and non-disease) showing the presence of 12 cell types based on the expression of specific markers. **(B)** Volcano plot and violin plots of differentially expressed genes (DEGs) in RPE/choroid of nAMD donor vs healthy non-disease donor

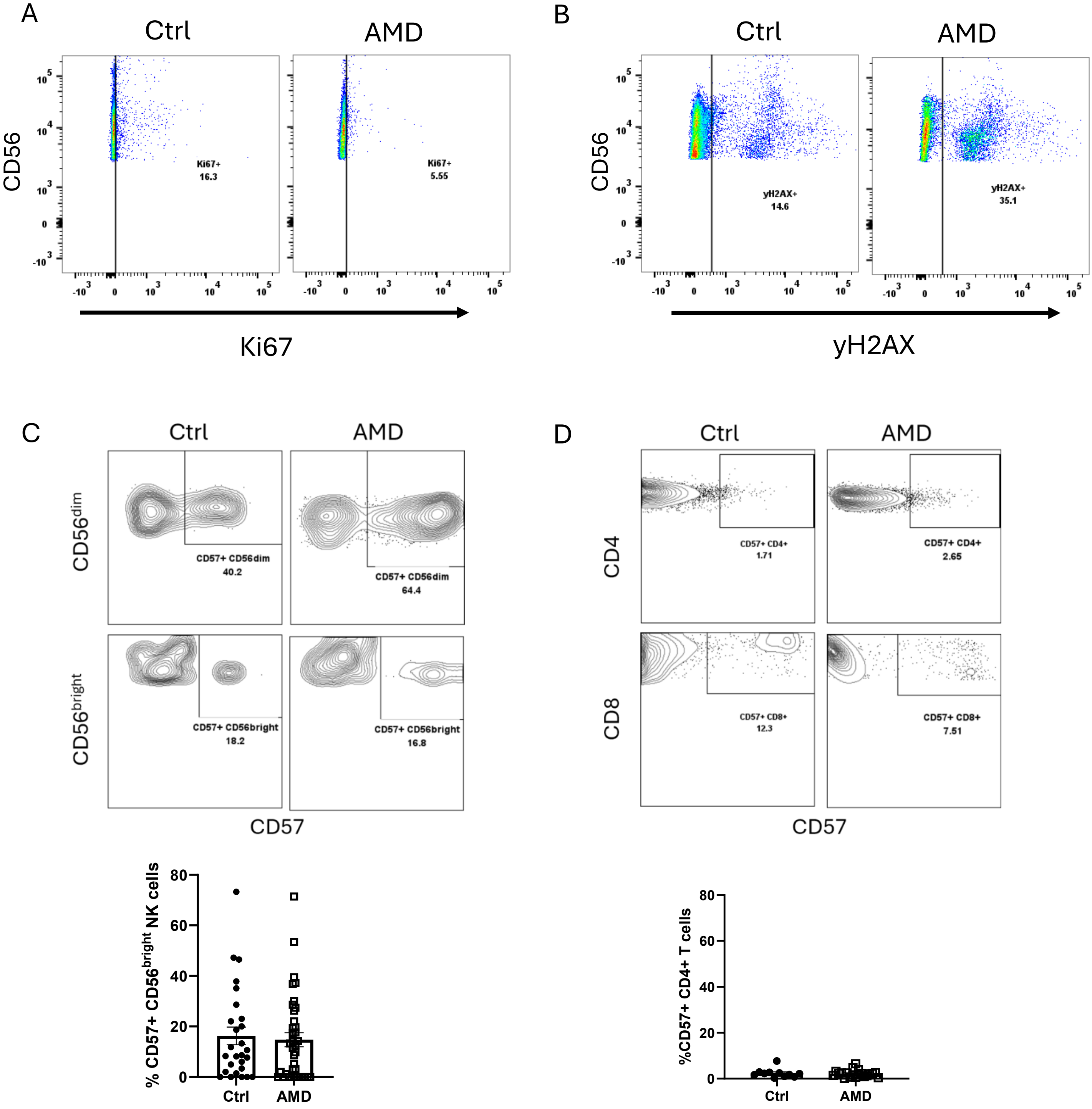

Supplementary Figure 9: Flow cytometric analysis of Ki67 and gH2AX on NK cells and CD57 expression on CD56^{bright} NK cells and CD4^{+} T cells. Related to Figure 6.

(A & B) Representative flow cytometry gating of Ki67 **(A)** and yH2AX **(B)** on CD3^{-}CD56^{+} NK cells in control or AMD donors. **(C)** Representative flow cytometry gating of CD57^{+} expression on CD56^{dim} NK cells (upper panel) and CD56^{bright} NK cells (lower panel)(n=29 Ctrl, n=37 AMD) **(D)** Representative flow cytometry gating of CD57^{+} cells on CD4^{+} (upper panel) and CD8^{+} (lower panel) T cells in control or AMD donors. CD57^{+} expression on CD4^{+} T cells (n=11 Ctrl, n=20 AMD). Graphical data are presented as mean ±SEM. $*P < 0.05$ $**P < 0.01$. Following normality testing, unpaired t-tests or one-way ANOVA with Dunnett's multiple comparison tests were used to compare groups.
